# Supplementary material for: Unravelling the sex-specific diversity and functions of adrenal gland macrophages
Source: Cell Rep. 2022 Jun 14;39(11):110949. doi: 10.1016/j.celrep.2022.110949 (PMC9210345; doi:10.1016/j.celrep.2022.110949)
Supplement: Document S1. Figures S1–S6 and Table S1 [file mmc1.pdf]

Cell Reports, Volume *39*

## Supplemental information

# Unravelling the sex-specific diversity

# and functions of adrenal gland macrophages

**Bastien Dolfi, Alexandre Gallerand, Maria M. Firulyova, Yingzheng Xu, Johanna Merlin, Adélie Dumont, Alexia Castiglione, Nathalie Vaillant, Sandrine Quemener, Heidi Gerke, Marion I. Stunault, Patricia R. Schrank, Seung-Hyeon Kim, Alisha Zhu, Jie Ding, Jerome Gilleron, Virginie Magnone, Pascal Barbry, David Dombrowicz, Christophe Duranton, Abdelilah Wakkach, Claudine Blin-Wakkach, Burkhard Becher, Sophie Pagnotta, Rafael J. Argüello, Pia Rantakari, Svetoslav Chakarov, Florent Ginhoux, Konstantin Zaitsev, Ki-Wook Kim, Laurent Yvan-Charvet, Rodolphe R. Guinamard, Jesse W. Williams, and Stoyan Ivanov**

## Sup. Table 1

| gene | p_val | avg_logFC (female over male) | pct.1 | pct.2 | p_val_adj |
|---|---|---|---|---|---|
| Xist | 0 | 1.54951258733711 | 0.828 | 0 | 0 |
| Rpl35 | 7.62774369759271e-40 | 0.332795131151709 | 0.975 | 0.916 | 1.1416443992187e-35 |
| Apoc2 | 3.69158130258448e-26 | 0.332692544859788 | 0.379 | 0.167 | 5.52518973557819e-22 |
| AA467197 | 6.22673290146039e-23 | 0.369648525877762 | 0.16 | 0.037 | 9.31955113361576e-19 |
| Lyz2 | 2.17567047442043e-22 | 0.386902156024932 | 0.999 | 0.999 | 3.25632599906506e-18 |
| Tsc22d3 | 6.49050153643698e-22 | 0.329842436397367 | 0.611 | 0.429 | 9.71433364958522e-18 |
| Fxyd5 | 8.32816037140203e-22 | 0.27680960279782 | 0.944 | 0.892 | 1.24647576278774e-17 |
| Ap1s2 | 1.73034399696515e-21 | 0.294384138791506 | 0.516 | 0.367 | 2.58980586025774e-17 |
| Sla | 3.91862476256913e-20 | 0.266959924479275 | 0.497 | 0.332 | 5.86500568213722e-16 |
| Ly6a | 1.0545988034916e-15 | 0.366701350118315 | 0.206 | 0.097 | 1.57841802918587e-11 |
| Cebpb | 8.00698976030681e-15 | 0.250943124974004 | 0.712 | 0.549 | 1.19840615742512e-10 |
| Msrb1 | 5.40275053801241e-14 | 0.275219903461956 | 0.558 | 0.398 | 8.08629673024318e-10 |
| Tmsb10 | 4.60848480745743e-12 | 0.380808925495099 | 0.801 | 0.725 | 6.89751921132154e-08 |
| Ms4a4c | 8.66898423345473e-11 | 0.25134064025716 | 0.4 | 0.286 | 1.29748687022117e-06 |
| Chil3 | 4.8237747978503e-10 | 0.394563532638321 | 0.186 | 0.087 | 7.21974373994254e-06 |
| Ifitm3 | 3.73254227181447e-08 | 0.266876571843615 | 0.88 | 0.835 | 0.000558649601822472 |
| Ly6c2 | 3.5363257330096e-07 | 0.29637517528339 | 0.18 | 0.097 | 0.00529281872459546 |
| Plac8 | 4.98692265535371e-07 | 0.460921076326878 | 0.294 | 0.193 | 0.0074639271382679 |
| Apoe | 7.50291402455461e-07 | 0.48830922785863 | 0.969 | 0.955 | 0.0112296114205509 |
| S100a6 | 1.08763499763158e-06 | 0.276562829675311 | 0.591 | 0.514 | 0.0162786330095518 |

**Table S1.**
List of genes differentially expressed among myeloid cells in female and male mice. This table is related to figure 2.

# Sup. Fig. 1

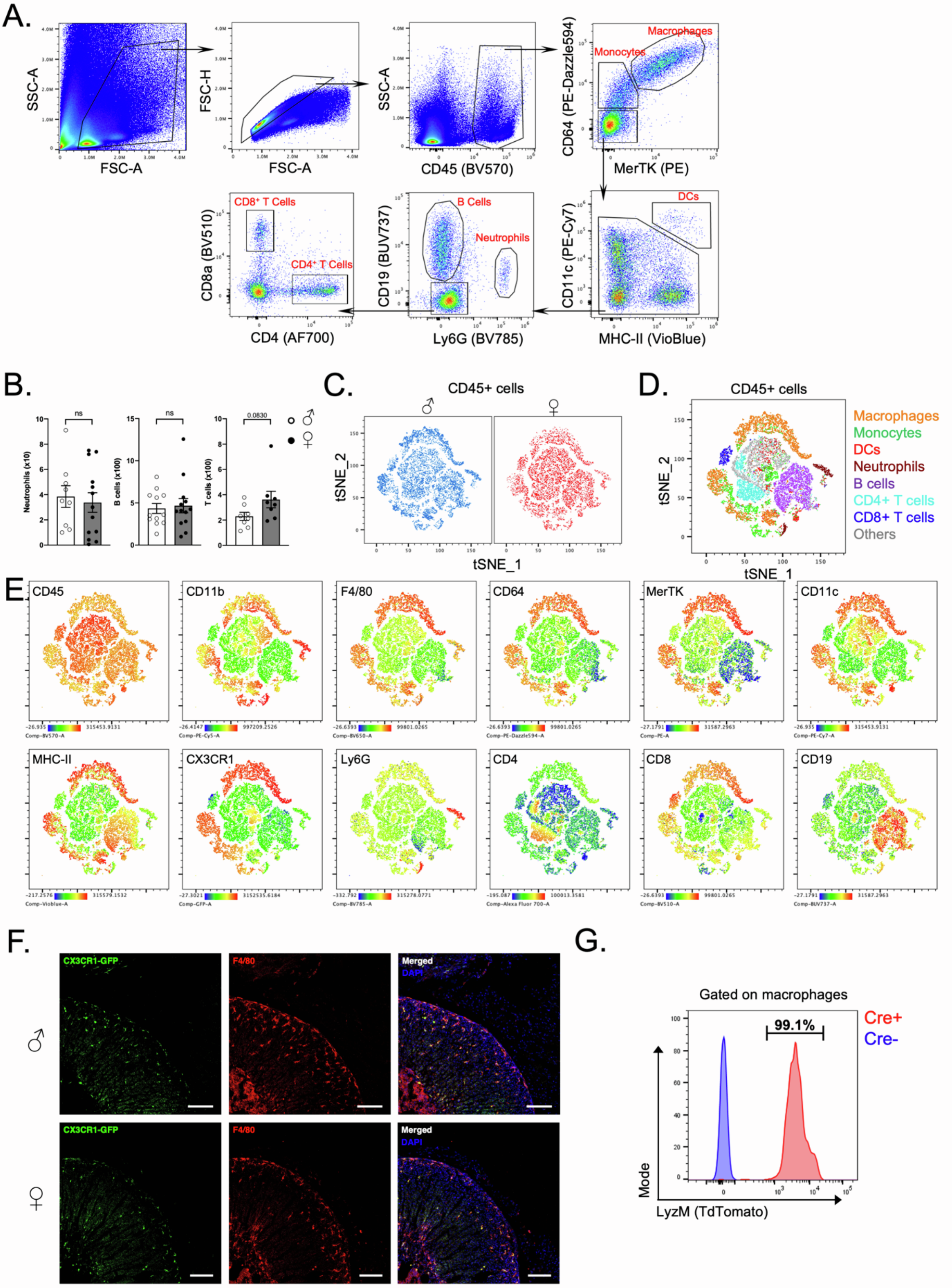

A.
SSC-A
FSC-A
FSC-H
SSC-A
CD45 (BV570)
CD64 (PE-Dazzle594)
MerTK (PE)
Macrophages
Monocytes
CD8a (BV510)
CD8+ T Cells
CD4+ T Cells
CD4 (AF700)
CD19 (BUV737)
B Cells
Neutrophils
Ly6G (BV785)
CD11c (PE-Cy7)
DCs
MHC-II (VioBlue)
B.
Neutrophils (x10)
B cells (x100)
T cells (x100)
ns
ns
0.0830
♂
♀
C.
CD45+ cells
♂
♀
tSNE_2
tSNE_1
D.
CD45+ cells
Macrophages
Monocytes
DCs
Neutrophils
B cells
CD4+ T cells
CD8+ T cells
Others
tSNE_2
tSNE_1
E.
CD45
CD11b
F4/80
CD64
MerTK
CD11c
MHC-II
CX3CR1
Ly6G
CD4
CD8
CD19
F.
CX3CR1-GFP
F4/80
Merged
DAPI
♂
♀
G.
Gated on macrophages
99.1%
Cre+
Cre-
Mode
LyzM (TdTomato)

**Figure S1. Flow cytometry analysis of immune cell diversity in male and female adrenal glands. This figure in complementary to figure 1.**

(**A**) Gating strategy used to identify adrenal gland immune cells. Concatenated data from 8 7-week-old female mice. (**B**) Quantification of neutrophils (♂ n=9 and ♀ n=13), B cells (♂ n=12 and ♀ n=13) and T cells (♂ n=8 and ♀ n=8) in male and female adrenal glands. Data pooled from one (T cells) or two (neutrophils and B cells) independent experiments. (**C**) Clustering of $CD45^+$ adrenal gland cells from male and female $CX3CR1^{GFP}$ mice using unsupervised t-distributed stochastic neighbor embedding (tSNE) analysis of flow cytometry data. Panel : CD40 (BUV395) ; CD24 (BUV496) ; CD80 (BUV615) ; CD19 (BUV737) ; F4/80 (BV650) ; Ly6G (BV785) ; CX3CR1 (GFP reporter) ; MerTK (PE) ; CD64 (PE-Dazzle594) ; CD11b (PE-Cy5) ; CD206 (PerCP-Cy5.5) ; Timd4 (PerCP-eFluor710) ; CD11c (PE-Cy7) ; Lyve1 (eFluor660) ; CD4 (AF700) ; CCR2 (APC-Fire750). (**D**) Identification of adrenal gland immune subsets. Cells were gated manually and overlaid on tSNE plot to identify clusters. Macrophages : $CD45^+$ $CD64^+$ $MerTK^+$. Monocytes : $CD45^+$ $MerTK^-$ $CD11b^+$ $CX3CR1^+$. Dendritic Cells (DCs) : $CD45^+$ $MerTK^-$ $CD11c^+$ $MHC\text{-}II^+$. Neutrophils : $CD45^+$ $CD11b^+$ $Ly6G^+$. B cells : $CD45^+$ $MerTK^-$ $CD11c^-$ $MHC\text{-}II^+$ $CD19^+$. CD4+ T cells : $CD45^+$ $MerTK^-$ $MHC\text{-}II^-$ $CD4^+$. CD8+ T cells : $CD45^+$ $MerTK^-$ $MHC\text{-}II^-$ $CD8^+$. (**E**) tSNE representation of the expression levels of CD45, CD11b, F4/80, CD64, MerTK, CD11c, MHC-II, CX3CR1, Ly6G, CD4, CD8 and CD19 among adrenal gland $CD45^+$ cells. (**F**) Microscopy analysis of F4/80 staining in adrenal glands of $CX3CR1^{GFP/+}$ mice. Scale bar : 100μm. (**G**) Flow cytometry analysis of TdTomato expression in adrenal gland macrophages from 8-week-old male $Lyz2^{cre}$ x $R26^{TdTomato}$ mice. Data from one experiment. All data are represented in means ± SEM. Statistical analysis was performed using two-tailed Mann-Whitney tests. ns p>0.05 ; * p<0.05 ; ** p<0.01 ; *** p<0.001 ; **** p<0.0001.

Sup. Fig. 2

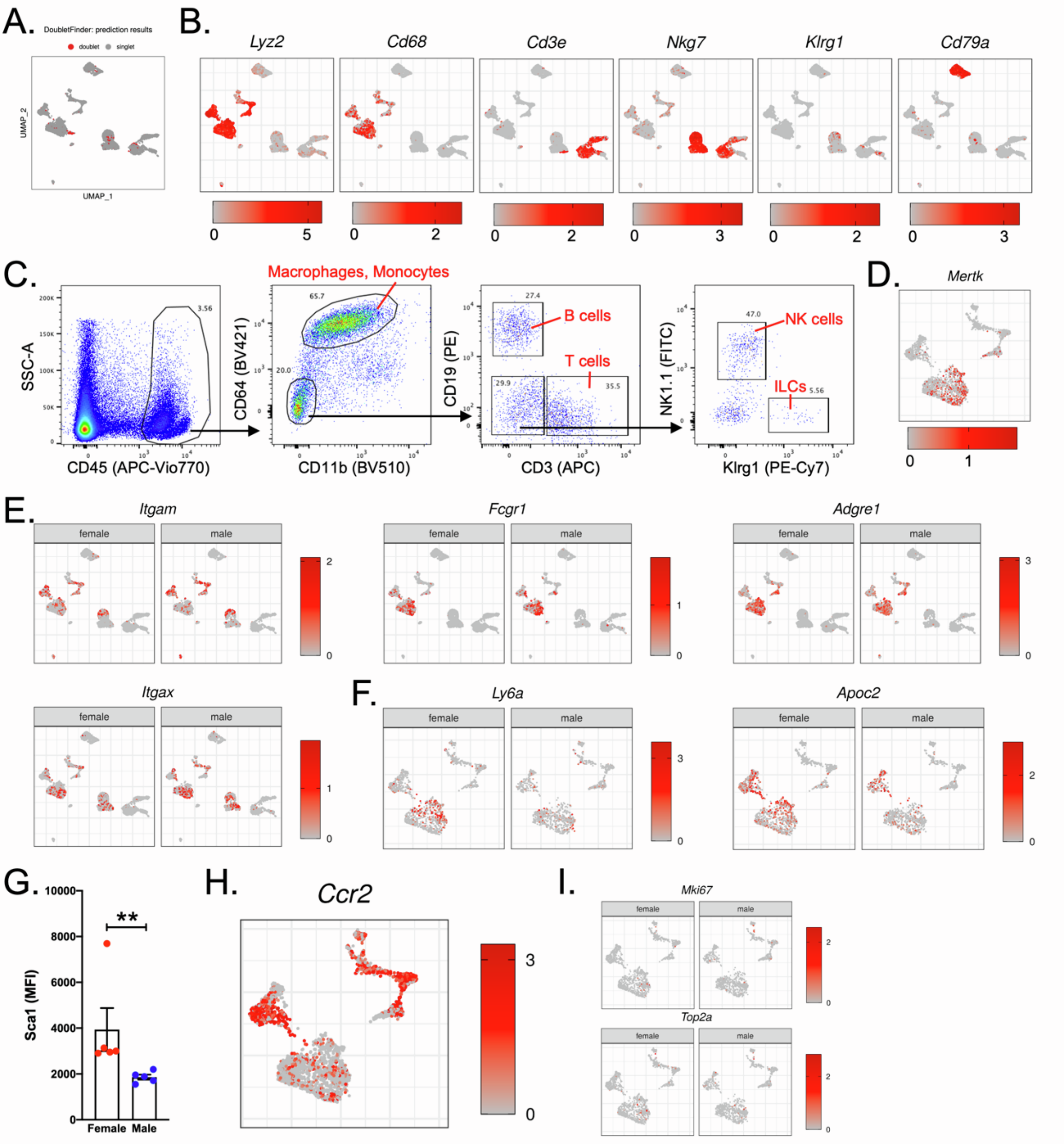
A.
DoubletFinder: prediction results
doublet
singlet
UMAP_2
UMAP_1
B.
Lyz2
Cd68
Cd3e
Nkg7
Klrg1
Cd79a
C.
SSC-A
CD45 (APC-Vio770)
3.56
Macrophages, Monocytes
65.7
20.0
CD64 (BV421)
CD11b (BV510)
27.4
B cells
T cells
29.9
35.5
CD19 (PE)
CD3 (APC)
47.0
NK cells
ILCs
5.56
NK1.1 (FITC)
Klrg1 (PE-Cy7)
D.
Mertk
E.
Itgam
Fcgr1
Adgre1
Itgax
female
male
F.
Ly6a
Apoc2
G.
**
Sca1 (MFI)
Female
Male
H.
Ccr2
I.
Mki67
Top2a

**Figure S2. Single cell RNA-seq analysis of adrenal gland immune cell diversity. This figure is complementary to figure 2.**

(**A**) Identification of doublets in scRNA-seq data using the DoubletFinder package. Doublets were removed from analysis. (**B**) scRNA-seq analysis of genes used to identify cell types (*Lyz2*, *CD68*, *CD3e*, *Nkg7*, *Klrg1* and *CD79a*). (**C**) Flow cytometry plots validating the presence of immune cell types corresponding to clusters identified in the scRNA-seq analysis. (**D**) scRNA-seq analysis of *Mertk* expression among adrenal gland myeloid cells. (**E**) scRNA-seq analysis of myeloid cell canonical markers by adrenal gland $CD45^+$ cells from 7-week-old male and female mice. (**F**) scRNA-seq analysis of sex-enriched genes in adrenal gland myeloid cells. (**G**) Flow cytometry analysis of Sca1 expression in female and male mice (n=5 mice per group). Data pooled from two independent experiments. (**H**) scRNA-seq analysis of *CCR2* expression among adrenal gland myeloid cells. (**I**) scRNA-seq analysis of *Mki67* and *Top2a* mRNA expression. All data are represented in means ± SEM. Statistical analysis was performed using two-tailed Mann-Whitney tests. ns $p>0.05$ ; * $p<0.05$ ; ** $p<0.01$ ; *** $p<0.001$ ; **** $p<0.0001$.

Sup. Fig. 3

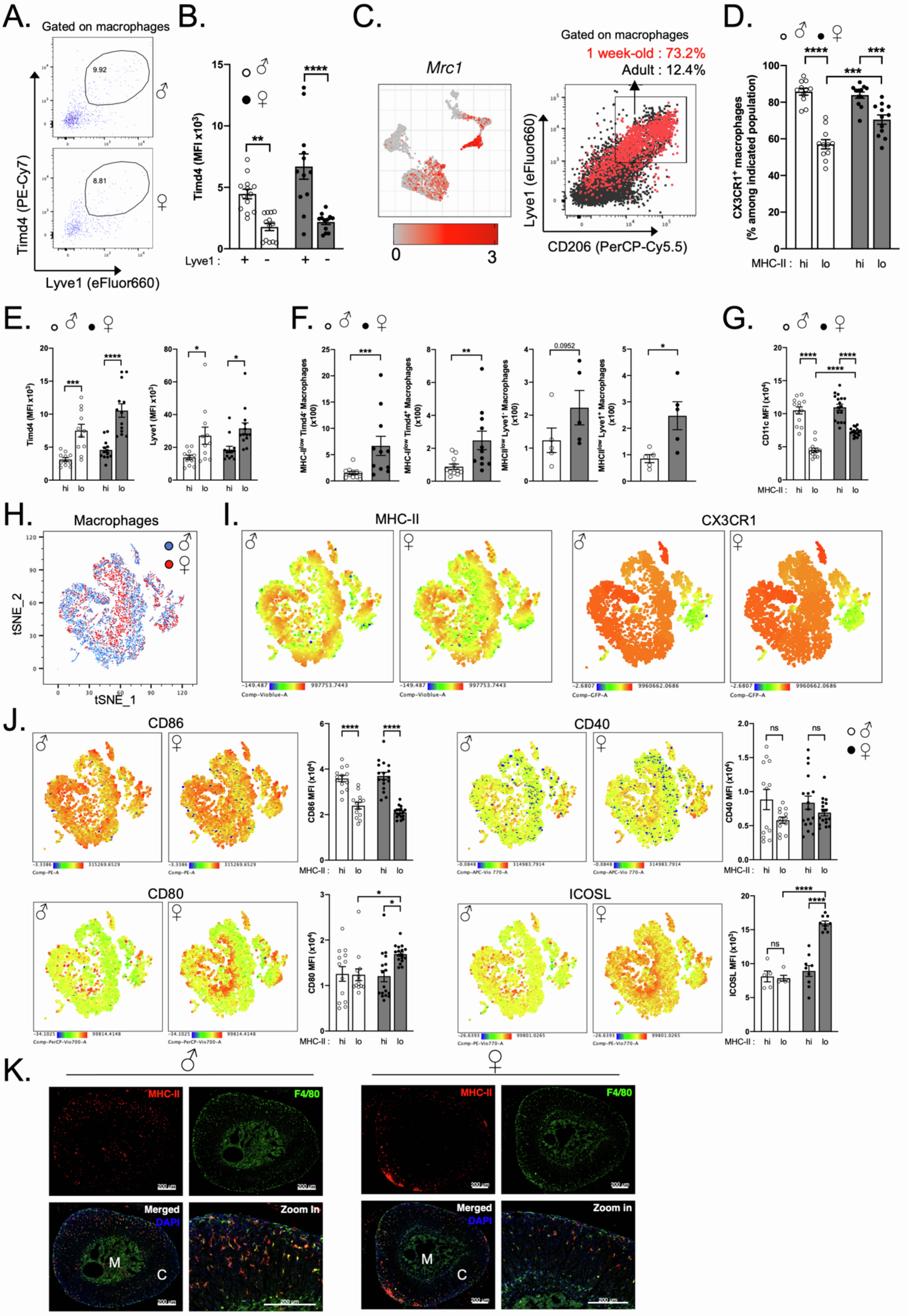
A.
Gated on macrophages
Timd4 (PE-Cy7)
Lyve1 (eFluor660)
9.92
8.81
B.
Timd4 (MFI x10³)
Lyve1 :
C.
Mrc1
Gated on macrophages
1 week-old : 73.2%
Adult : 12.4%
Lyve1 (eFluor660)
CD206 (PerCP-Cy5.5)
D.
CX3CR1⁺ macrophages (% among indicated population)
MHC-II :
hi
lo
E.
Timd4 (MFI x10³)
Lyve1 (MFI x10³)
F.
0.0952
G.
CD11c MFI (x10⁴)
H.
Macrophages
tSNE_2
tSNE_1
I.
MHC-II
CX3CR1
J.
CD86
CD40
CD80
ICOSL
CD86 MFI (x10⁴)
CD40 MFI (x10⁴)
CD80 MFI (x10⁴)
ICOSL MFI (x10³)
K.
MHC-II
F4/80
Merged
DAPI
Zoom In
M
C

**Figure S3. MHC-II expression identifies two macrophage populations with sex-specific abundance and distribution. This figure is complementary to figures 3 and 4.**

(**A**) Representative plots showing adrenal gland macrophage Timd4 and Lyve1 expression in male and female mice. Data representative of at least 3 independent experiments. (**B**) Mean Timd4 fluorescence intensity among Lyve1$^+$ and Lyve1$^-$ macrophage subsets in male and female mice. ♂ n=13 and ♀ n=12. Data pooled from 2 independent experiments. (**C**) (Left) scRNA-seq analysis of *Mrc1* mRNA expression and (Right) representative plot showing adrenal gland macrophage Lyve1 and CD206 expression in 1- and 9-week-old male mice. Data from one experiment. (**D**) Proportions of CX3CR1$^+$ cells among MHC-II$^{high}$ and MHC-II$^{low}$ adrenal gland macrophages from male and female CX3CR1$^{GFP}$ mice. ♂ n=11 and ♀ n=12. Data pooled from 2 independent experiments. (**E**) Mean Timd4 and Lyve1 fluorescence intensity among MHC-II$^{high}$ and MHC-II$^{low}$ adrenal gland macrophages from male and female mice. ♂ n=12 and ♀ n=13. Data pooled from 2 independent experiments. (**F**) Quantification of MHC-II$^{low}$ adrenal gland macrophage subsets according to Lyve1 and Timd4 expression in 7-week-old male and female mice. Timd4 subsets : ♂ n=11 and ♀ n=11. Data pooled from 2 independent experiments. Lyve1 subsets : ♂ n=5 and ♀ n=5. Data from one experiment. (**G**) Mean CD11c fluorescence intensity among MHC-II$^{high}$ and MHC-II$^{low}$ adrenal gland macrophages from male and female mice. ♂ n=13 and ♀ n=17. Data pooled from 3 independent experiments. (**H**) Clustering of adrenal gland macrophages from male and female CX3CR1$^{GFP}$ mice using unsupervised t-distributed stochastic neighbor embedding (tSNE) analysis of flow cytometry data. Panel : CD24 (BUV496) ; CD19 (BUV737) ; MHC-II (VioBlue) ; CD11b (BV510) ; CD45 (BV570) ; F4/80 (BV650) ; CX3CR1 (GFP reporter) ; CD86 (PE) ; CD64 (PE-Dazzle594) ; CD11c (PE-Cy5) ; CD80 (PerCP-Vio700) ; ICOSL (PE-Vio770) ; Ly6C (APC) ; CD40 (APC-Vio770). Data representative of 3 independent experiments. (**I**) tSNE representation of MHC-II and CX3CR1 expression levels in adrenal gland macrophages from male and female CX3CR1$^{GFP}$ mice. Data representative of 3 independent experiments. (**J**) tSNE representation and quantification of CD86, CD80, CD40 and ICOSL expression levels in adrenal gland macrophages from male and female CX3CR1$^{GFP}$ mice. ♂ n=13 and ♀ n=17. Pooled data from 2 (ICOSL) or 3 (CD86, CD80, CD40) independent experiments. (**K**) Fluorescence microscopy analysis of F4/80 and MHC-II expression in adrenal glands from 7-week-old male and female wild-type mice. Scale bar : 200μm. M (medulla); C (cortex). Data representative of at least 3 independent experiments. All data are represented in means ± SEM. Statistical analysis was performed using two-way ANOVA with Bonferroni's post-test (panels B, D, E, F, G and J) or two-tailed Mann-Whitney tests (panel G). ns p>0.05 ; * p<0.05 ; ** p<0.01 ; *** p<0.001 ; **** p<0.0001.

Sup. Fig. 4

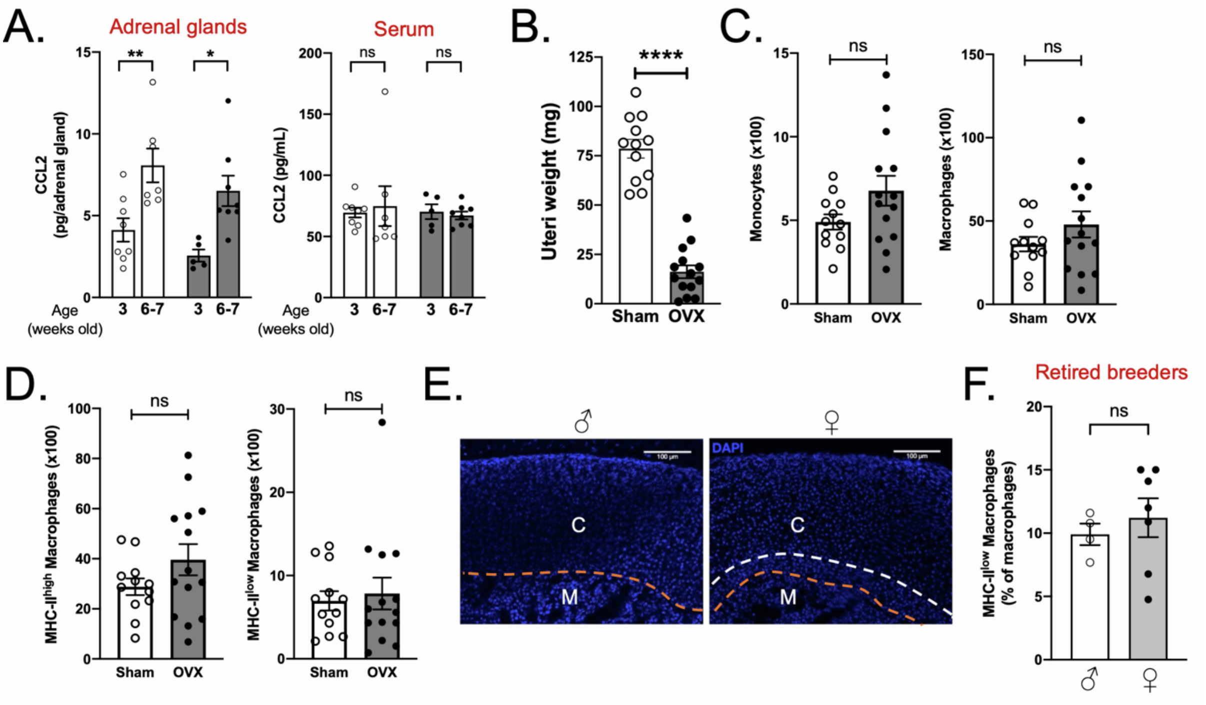


**Figure S4. Ovariectomy does not affect adrenal gland macrophage content. This figure is complementary to figure 5.**

(**A**) CCL2 levels in adrenal gland homogenates and serum from 3 (♂ n=8 and ♀ n=5) or 6 to 7 (♂ n=7 and ♀ n=8) week-old male and female wild-type mice. Data pooled from 4 independent experiments. (**B**) Weight of uteri from sham-operated (n=12) and OVX (n=14) mice. Data pooled from 2 independent experiments. (**C**) Quantification of adrenal gland monocytes and macrophages in ovariectomized (n=14) and sham-operated (n=12) wild-type mice. Data pooled from 2 independent experiments. (**D**) Quantification of adrenal gland MHC-II$^{low}$ and MHC-II$^{high}$ macrophages in ovariectomized (n=14) and sham-operated (n=12) wild-type mice. Data pooled from 2 independent experiments. (**E**) Confocal microscopy analysis of adrenal glands from male and female 4-week-old wild-type mice. Orange dots indicate the border between the cortex (C) and the medulla (M). The X-Zone is comprised between white and orange dots. Scale bar : 100um. (**F**) Proportions of MHC-II$^{low}$ adrenal gland macrophages in male and female retired breeders (♂ n=4 and ♀ n=7). Data pooled from 2 independent experiments. All data are represented in means ± SEM. Statistical analysis was performed using two-way ANOVA with Bonferroni's post-test (panel A) or two-tailed Mann-Whitney tests (panels B, C, D and F). ns p>0.05 ; * p<0.05 ; ** p<0.01 ; *** p<0.001 ; **** p<0.0001.

Sup. Fig. 5

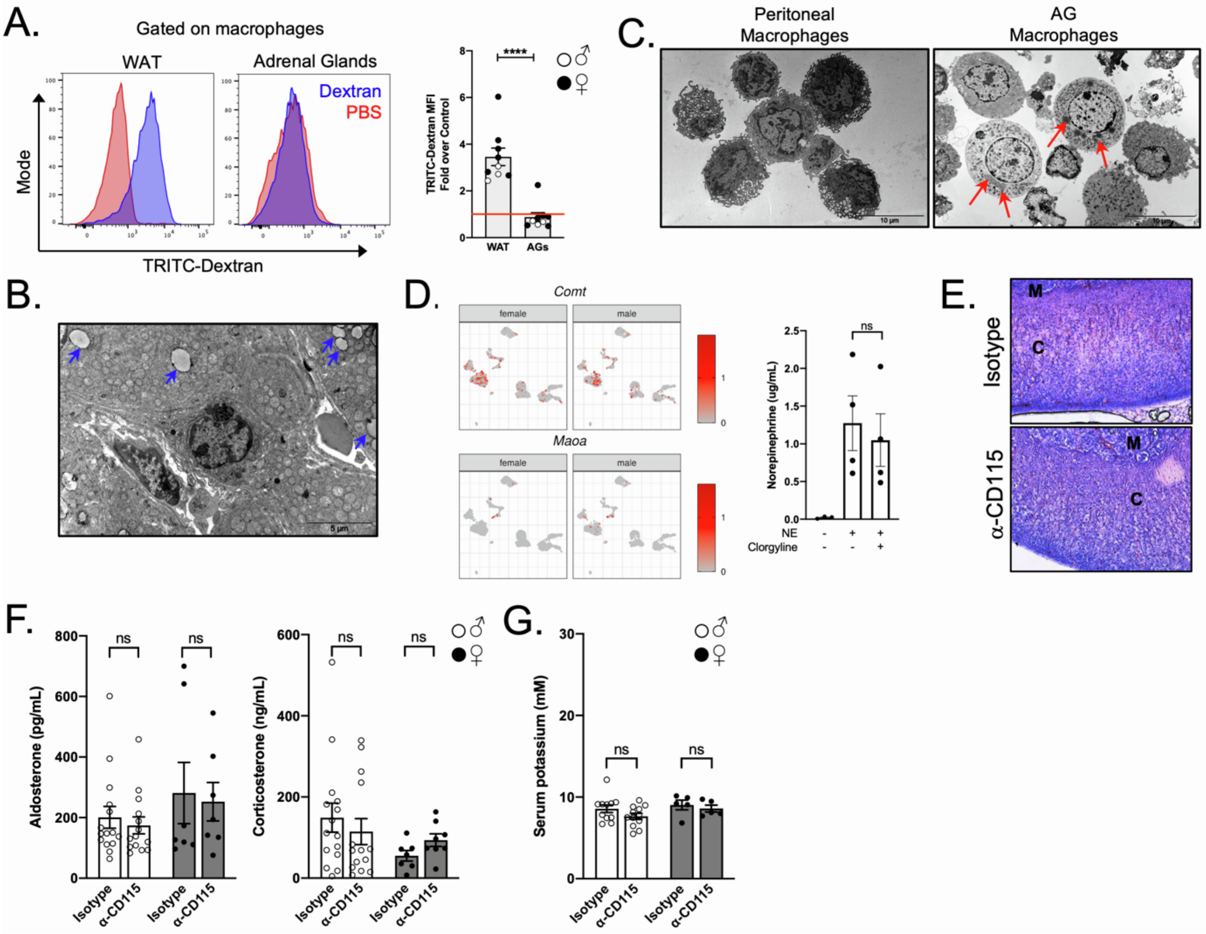

A.
Gated on macrophages
WAT
Adrenal Glands
Dextran
PBS
Mode
TRITC-Dextran
TRITC-Dextran MFI Fold over Control
****
WAT
AGs
B.
C.
Peritoneal Macrophages
AG Macrophages
D.
Comt
female
male
Maoa
Norepinephrine (ug/mL)
ns
NE
Clorgyline
E.
Isotype
α-CD115
M
C
F.
Aldosterone (pg/mL)
Corticosterone (ng/mL)
ns
Isotype
α-CD115
G.
Serum potassium (mM)

**Figure S5. Macrophage depletion does not alter adrenal gland architecture and hormone production at steady state. This figure is complementary to figure 6.**
(**A**) Representative flow cytometry plots (left) and quantification (right) of TRITC-Dextran uptake by adipose tissue and adrenal gland macrophages 20 minutes after intravenous injection of 1mg 65-85kDa TRITC-Dextran. (♂ n=3 and ♀ n=6). Data from one experiment. (**B**) Electron microscopy analysis of adrenal gland macrophages. Blue arrows indicate the presence of lipid droplets in stroma. Scale bar : 5μm. (**C**) Electron microscopy analysis of purified peritoneal and adrenal gland macrophages. Red arrows indicate the presence of lipid droplets in macrophages. Scale bar : 10μm. Data from one experiment. (**D**) (Left) scRNA-seq analysis of *Maoa* and *Comt* mRNA expression and (Right) norepinephrine levels after overnight culture of cell-sorted adrenal gland macrophages in the presence/absence of norepinephrine and/or clorgyline. n=3 (baseline) or 4 (+NE) biological replicates. Data from 3 pooled experiments. (**E**) Microscopy analysis of adrenal gland morphology in isotype control-treated and α–CD115-treated wild-type mice using H&E staining. Data from one experiment. (**F**) Serum aldosterone and corticosterone levels in isotype control-treated (♂ n=15 and ♀ n=7) and α–CD115-treated (♂ n=14 and ♀ n=7-8) male and female wild-type mice. (**G**) Serum $K^+$ levels in isotype control-treated (♂ n=11 and ♀ n=5) and α–CD115-treated (♂ n=12 and ♀ n=5) male and female wild-type mice. Pooled data from 3 independent experiments. Statistical analysis was performed using two-way ANOVA with Bonferroni's post-test (panels F and G), one-way ANOVA with Bonferroni's post-test (panel D) or two-tailed Mann-Whitney tests (panel A). ns p>0.05 ; * p<0.05 ; ** p<0.01 ; *** p<0.001 ; **** p<0.0001.

Sup. Fig. 6

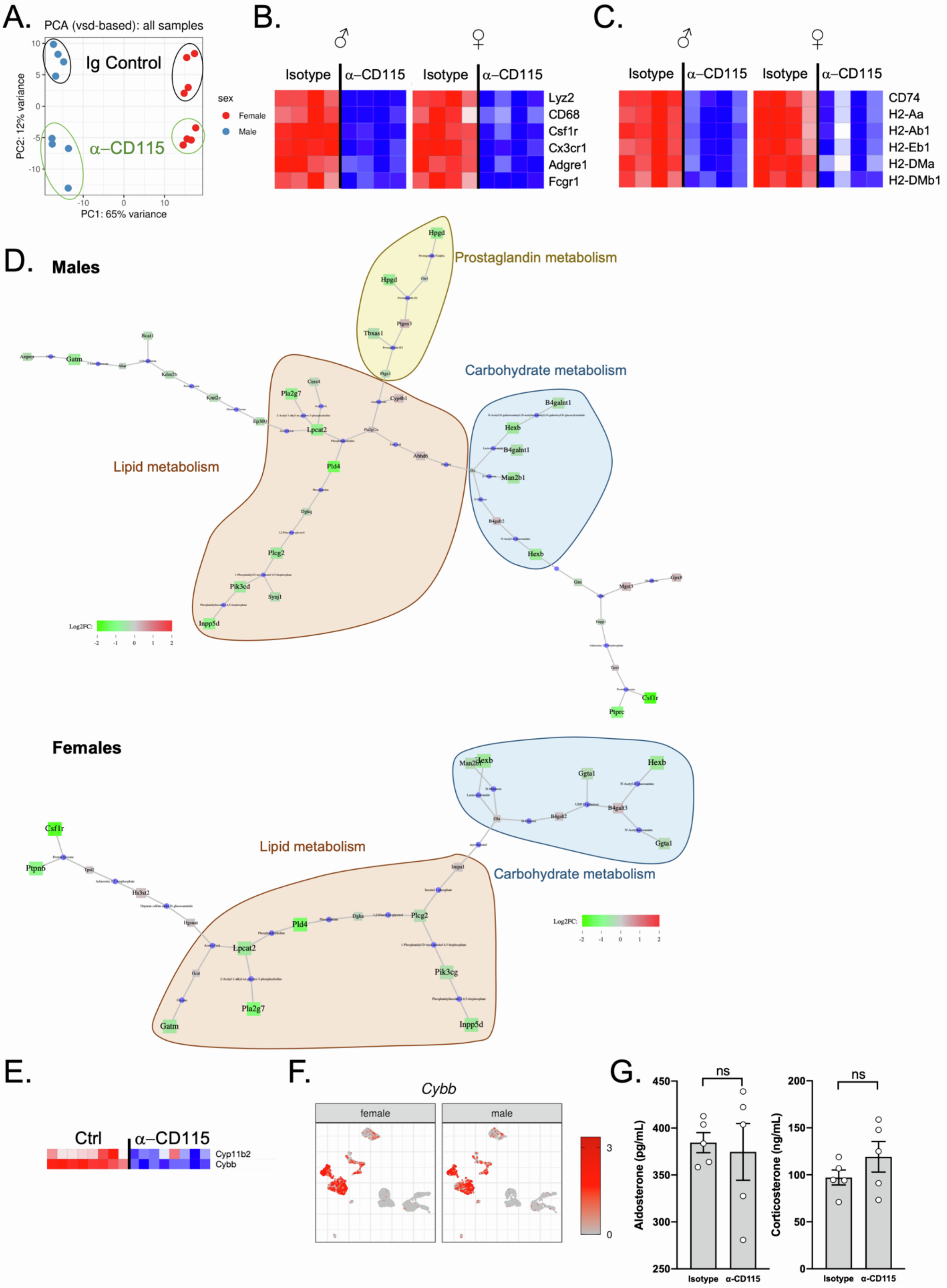

A.
PCA (vsd-based): all samples
Ig Control
α–CD115
sex
Female
Male
PC2: 12% variance
PC1: 65% variance
B.
♂
♀
Isotype
α–CD115
Lyz2
CD68
Csf1r
Cx3cr1
Adgre1
Fcgr1
C.
CD74
H2-Aa
H2-Ab1
H2-Eb1
H2-DMa
H2-DMb1
D.
Males
Prostaglandin metabolism
Carbohydrate metabolism
Lipid metabolism
Log2FC:
Females
E.
Ctrl
α–CD115
Cyp11b2
Cybb
F.
Cybb
female
male
G.
ns
Aldosterone (pg/mL)
Corticosterone (ng/mL)
Isotype
α-CD115

**Figure S6. Macrophage depletion affects adrenal gland lipid metabolism. This figure is complementary to figure 6.**
(**A**) Principal Component Analysis (PCA) of adrenal gland bulk RNA-seq from isotype control-treated and α–CD115-treated male and female wild-type mice. (**B**) RNA-seq analysis of myeloid cell-related genes in adrenal glands from isotype control-treated and α–CD115-treated male and female wild-type mice. (**C**) RNA-seq analysis of MHC-II-related genes in adrenal glands from isotype control-treated and α–CD115-treated male and female wild-type mice. (**D**) Transcriptional analysis of metabolic pathways in adrenal glands from α–CD115 or isotype control treated male and female mice. The size of nodes represent the significance of changes in gene expression : bigger size of nodes means lower p-values. A high resolution version of this panel is supplied as supplementary item 3. (**E**) RNA-seq analysis of *Cyp11b2* and *Cybb* expression in adrenal glands from isotype control-treated and α–CD115-treated wild-type mice. (**F)** scRNA-seq analysis of *Cybb* mRNA expression. (**G**) Serum aldosterone and corticosterone levels in α–CD115 (n=5) or isotype control-treated (n=5) female wild-type mice submitted to a 12-hour cold challenge. Data from one experiment. Statistical analysis was performed using two-tailed Mann-Whitney tests (panel G). ns $p>0.05$.
