# Supplementary figures and images for: Unravelling the sex-specific diversity and functions of adrenal gland macrophages
Source: Cell Rep. 2022 Jun 14;39(11):110949. doi: 10.1016/j.celrep.2022.110949 (PMC9210345; doi:10.1016/j.celrep.2022.110949)

## Females

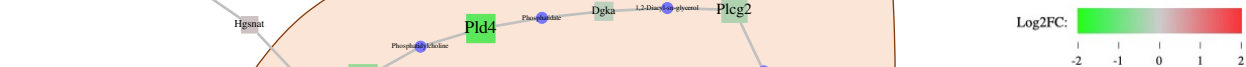

## Lipid metabolism

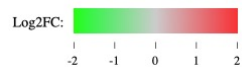

Supplement: Data S3. Higher-resolution version of Figure S6D, related to Figure S6 [file mmc4.pdf]
