# Supplementary material for: Unravelling the sex-specific diversity and functions of adrenal gland macrophages
Source: Cell Rep. 2022 Jun 14;39(11):110949. doi: 10.1016/j.celrep.2022.110949 (PMC9210345; doi:10.1016/j.celrep.2022.110949)
Supplement: Document S2. Article plus supplemental information [file mmc5.pdf]

Cell Reports

Article

Unravelling the sex-specific diversity and functions of adrenal gland macrophages

Graphical abstract

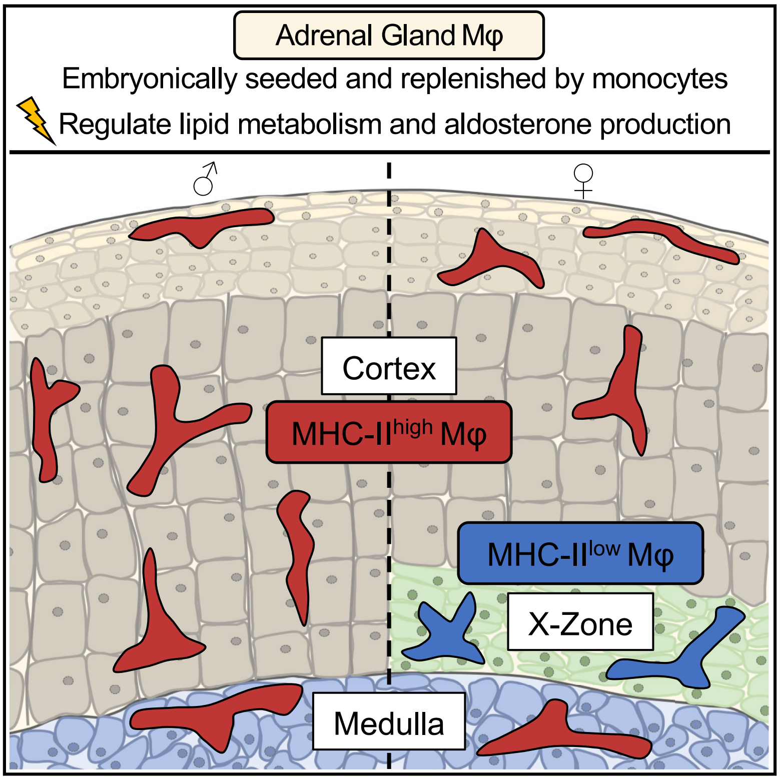

Highlights

- Adrenal glands contain multiple macrophage populations
- Macrophage sex dimorphism depends on the presence of the cortical X zone
- Embryonic and monocyte-derived macrophages co-exist in adrenal glands
- Adrenal gland macrophage depletion alters tissue lipid metabolism

Authors

Bastien Dolfi, Alexandre Gallerand, Maria M. Firulyova, ..., Rodolphe R. Guinamard, Jesse W. Williams, Stoyan Ivanov

Correspondence

jww@umn.edu (J.W.W.), stoyan.ivanov@unice.fr (S.I.)

In brief

Dolfi et al. uncover the sex-specific heterogeneity of adrenal gland macrophages and their ontogeny, maintenance, tissue distribution, and role in local lipid homeostasis.

Dolfi et al., 2022, Cell Reports *39*, 110949
June 14, 2022 © 2022 The Author(s).
https://doi.org/10.1016/j.celrep.2022.110949

CellPress

Cell Reports

CellPress
OPEN ACCESS

Article

Unravelling the sex-specific diversity and functions of adrenal gland macrophages

Bastien Dolfi,[1,9,17] Alexandre Gallerand,[1,9,17] Maria M. Firulyova,[2] Yingzheng Xu,[3] Johanna Merlin,[1] Adélie Dumont,[1] Alexia Castiglione,[1] Nathalie Vaillant,[1] Sandrine Quemener,[4] Heidi Gerke,[5,6] Marion I. Stunault,[1] Patricia R. Schrank,[3] Seung-Hyeon Kim,[7] Alisha Zhu,[3] Jie Ding,[7] Jerome Gilleron,[1] Virginie Magnone,[8] Pascal Barbry,[8] David Dombrowicz,[4] Christophe Duranton,[9] Abdelilah Wakkach,[9] Claudine Blin-Wakkach,[9] Burkhard Becher,[10] Sophie Pagnotta,[11] Rafael J. Argüello,[12] Pia Rantakari,[5,6] Svetoslav Chakarov,[13] Florent Ginhoux,[13,14,15,16] Konstantin Zaitsev,[2] Ki-Wook Kim,[7] Laurent Yvan-Charvet,[1] Rodolphe R. Guinamard,[1,9] Jesse W. Williams,[3,*] and Stoyan Ivanov[1,9,18,*]

[1]Université Côte d'Azur, INSERM, C3M, Nice, France
[2]Computer Technologies Department, ITMO University, Saint Petersburg, Russia
[3]Center for Immunology, Department of Integrative Biology and Physiology, University of Minnesota Medical School, Minneapolis, MN, USA
[4]Univ.Lille, INSERM, CHU Lille, Institut Pasteur de Lille, U1011-EGID, 59000 Lille, France
[5]Turku Bioscience Centre, University of Turku and Åbo Akademi University, Turku, Finland
[6]InFLAMES Research Flagship Center, University of Turku, Turku, Finland
[7]Department of Pharmacology and Regenerative Medicine, University of Illinois College of Medicine, Chicago, IL, USA
[8]Université Côte d'Azur, CNRS, IPMC, Valbonne, France
[9]Université Côte d'Azur, CNRS, LP2M, Nice, France
[10]Institute of Experimental Immunology, University of Zürich, Switzerland
[11]Université Côte d'Azur, Centre Commun de Microscopie Appliquée (CCMA), Parc Valrose, 06108 Nice, France
[12]Aix Marseille Université, CNRS, INSERM, CIML, Centre d'Immunologie de Marseille-Luminy, Marseille, France
[13]Shanghai Institute of Immunology, Shanghai Jiao Tong University School of Medicine, Shanghai 200025, China
[14]Singapore Immunology Network (SIgN), Agency for Science, Technology and Research (A(*)STAR), Singapore 138648, Singapore
[15]Department of Microbiology and Immunology, Immunology Translational Research Program, Yong Loo Lin School of Medicine, Immunology Program, Life Sciences Institute, National University of Singapore, Singapore 117543, Singapore
[16]Translational Immunology Institute, SingHealth Duke-NUS Academic Medical Centre, Singapore 169856, Singapore
[17]These authors contributed equally
[18]Lead contact
*Correspondence: jww@umn.edu (J.W.W.), stoyan.ivanov@unice.fr (S.I.)
https://doi.org/10.1016/j.celrep.2022.110949

SUMMARY

Despite the ubiquitous function of macrophages across the body, the diversity, origin, and function of adrenal gland macrophages remain largely unknown. We define the heterogeneity of adrenal gland immune cells using single-cell RNA sequencing and use genetic models to explore the developmental mechanisms yielding macrophage diversity. We define populations of monocyte-derived and embryonically seeded adrenal gland macrophages and identify a female-specific subset with low major histocompatibility complex (MHC) class II expression. In adulthood, monocyte recruitment dominates adrenal gland macrophage maintenance in female mice. Adrenal gland macrophage sub-tissular distribution follows a sex-dimorphic pattern, with MHC class II^{low} macrophages located at the cortico-medullary junction. Macrophage sex dimorphism depends on the presence of the cortical X-zone. Adrenal gland macrophage depletion results in altered tissue homeostasis, modulated lipid metabolism, and decreased local aldosterone production during stress exposure. Overall, these data reveal the heterogeneity of adrenal gland macrophages and point toward sex-restricted distribution and functions of these cells.

INTRODUCTION

Tissue resident macrophages play a key role in health and disease (Cox et al., 2021). Macrophages are present at diverse frequencies in all tissues, and they are identified by the expression of the surface receptors CD64 and MerTK (Gautier et al., 2012). Their functions can be organ specific and are dictated by the local microenvironment. For example, brown adipose tissue (BAT) macrophages regulate adipocyte heat production, and spleen macrophages capture iron (Kohyama et al., 2009; Wolf et al., 2017). Tissue-resident macrophages originate from embryonic or bone marrow precursors (Epelman et al., 2014). For instance, microglia at steady state are strictly derived from yolk sac progenitors, while intestinal lamina propria macrophages are entirely monocyte derived (Bain et al., 2014; Ginhoux et al., 2010; Gomez Perdiguero et al., 2015; Schulz et al., 2012). In general, populations of embryonically and bone-marrow-derived cells co-exist in adult peripheral tissues (Ensan et al., 2016). A

© 2022 The Author(s).
This is an open access article under the CC BY license (http://creativecommons.org/licenses/by/4.0/).

seminal work in heart established that a self-renewing tissue-resident macrophage subset possesses tissue-remodeling functions during inflammatory events, whereas monocyte-derived cells have a pro-inflammatory phenotype (Dick et al., 2019). During aging, monocyte-derived macrophages progressively replace embryonically derived macrophages (Ginhoux and Guilliams, 2016; Molawi et al., 2014). Sex-specific differences in macrophage maintenance have been documented, suggesting that macrophage populations might display a sex-dependent diversity and function (Bain et al., 2016, 2020). Among other tissues, macrophages have been described in endocrine organs including pancreas, testis, and ovaries (Calderon et al., 2015; Ferris et al., 2017; Jokela et al., 2020; Lokka et al., 2020; Mossadegh-Keller et al., 2017). Yet, the diversity, phenotype, and functions of endocrine organ macrophages remain to be completely elucidated.

The adrenal gland is composed of a medulla and a cortex. In humans, the cortex can be further divided into three zones: zona glomerulosa, zona fasciculata, and zona reticularis. In mice, the zona reticularis is called the X zone and displays sex-dependent regulation. Mouse adrenal glands present sexually dimorphic organization, size, and functions (Lyraki and Schedl, 2021). Female adrenal glands are heavier than their male counterparts, which are characterized by a slower growth rate (Bielohuby et al., 2007). Importantly, adrenal gland pathologies occur earlier in life and with higher incidence in women than in men (Audenet et al., 2013; Lacroix et al., 2015). The presence of F4/80^{+} cells, likely macrophages, was demonstrated in adrenal glands by a pioneering work (Hume et al., 1984). However, recent reports established that F4/80 expression was shared among several myeloid cell types, including macrophages, monocytes, dendritic cells (DCs), and eosinophils (Ginhoux et al., 2009). Whether the sex-specific regulation of adrenal gland tissue homeostasis is paralleled by sex dimorphism in macrophage diversity and function is not yet documented. Furthermore, adrenal gland immune cell sex dimorphism could also contribute to adrenal pathologies initiation and progression.

RESULTS

Adrenal gland macrophage identification and topology

To evaluate immune cell diversity in adrenal glands, we performed a flow-cytometry analysis of adrenal gland CD45^{+} cells from 7-week-old C57BL/6 mice, with a particular focus on macrophage and monocyte subsets. Macrophages were identified as CD64^{+}MerTK^{+} cells and monocytes as CD64^{+}MerTK^{-} cells (Figure S1A) (Jakubzick et al., 2013). Neutrophils were scarce in this tissue, suggesting low blood contamination in our analysis (Figure S1A, bottom panel). The adrenal gland is composed of a cortex and a medulla responsible for selective hormone production. Because adrenal glands display a sex-specific organization in adult mice, we analyzed both female and male animals. Although macrophage numbers were similar in both sexes, higher monocyte counts tended to be detected in females (Figure 1A). DCs, identified as CD64^{-}MerTK^{-}MHC-II^{+}CD11c^{+} cells, were also present in low numbers in adrenal glands without sex-specific differences (Figures 1A and S1A). Neutrophil, B cell, and T cell counts were similar between female and male mice (Figure S1B). To further characterize the phenotype of adrenal gland immune cells in female and male mice, we performed spectral flow cytometry (Figure S1C). Unsupervised analysis confirmed the presence of diverse immune cell clusters in adrenal glands (Figure S1D). These clusters corresponding to populations of macrophages, monocytes, DCs, neutrophils, B cells, and CD4^{+} and CD8^{+} T cells expressed characteristic markers (Figure S1E). Notably, adrenal gland macrophages (AGMs) uniformly expressed F4/80 and the integrins CD11b and CD11c (Figures 1B and S1E).

Next, we investigated macrophage topologic distribution. F4/80^{+} cells were observed both in the adrenal gland cortex and medulla (Figure S1F). To further validate that these cells were macrophages, we used macrophage reporter strains, including Lyz2^{cre} x R26^{TdTomato} and CD115^{creERT2} x R26^{TdTomato} mice. Virtually 100% of macrophages were labeled in Lyz2^{cre} x R26^{TdTomato} mice (Figure S1G). In those models, we found that R26$^{TdTomato+}$ macrophages were present in both the adrenal cortex and the medulla (Figures 1C and 1D). Using CX3CR1$^{GFP/+}$ reporter mice, we found that CX3CR1 was expressed by most AGMs in both female and male mice (Figures 1E and S1F). The number of CX3CR1^{+} macrophages was similar in female and male mice (Figure 1E). The distribution of CX3CR1^{GFP+} cells in adrenal glands followed a sex-specific pattern, with a quasi-uniform distribution in both cortex and medulla in male mice (Figure 1F). By contrast, in females, CX3CR1^{GFP+} cells were preferentially located under the adrenal capsule and at the border between the cortex and the medulla (Figure 1F). These data revealed a sex-specific pattern of AGM distribution.

AGM diversity and mechanisms controlling their homeostatic maintenance

To obtain further insights on the sex-specific myeloid cell diversity in adrenal glands and their specific functions, we performed single-cell RNA sequencing (scRNA-seq) analysis on cell-sorted CD45^{+} cells isolated from 7-week-old C57BL/6 female and male mice (Figure 2A). Doublet contamination was identified and removed from the analysis (Figure S2A). Data from female and male mice were integrated with Seurat and projection maps made using uniform manifold approximation and projection (UMAP). Our data revealed a diversity in immune cell populations residing within the adrenal glands of both sexes (Figures 2A and S2B). In line with our flow-cytometry data, monocytes, DCs, neutrophils, B cells, and T cells were present in adrenal glands (Figure 2A). Natural killer (NK) cells and a population of innate lymphoid cells (ILCs) were also identified (Figure 2A). Flow-cytometry analysis confirmed the presence of NK cells and Klrg1^{+}NK1.1^{-}CD3^{-} ILCs in adrenal glands (Figure S2C). Importantly, and in agreement with our flow-cytometry observations, macrophages were the most abundant immune cell type (Figures 2A and 2B). We observed the presence of 4 separate and well-defined macrophage clusters (clusters 2–5) in both female and male mice (Figure 2A). All macrophage clusters expressed Mertk mRNA (Figure S2D). Our scRNA-seq data revealed that all four macrophage populations also expressed the canonical macrophage markers *Lyz2*, *CD68*, CD11b (*Itgam*), CD64 (*Fcgr1*), and F4/80 (*Adgre1*) in both sexes (Figures S2B and S2E). CD11c (Itgax) was also expressed on AGMs

CellPress
OPEN ACCESS

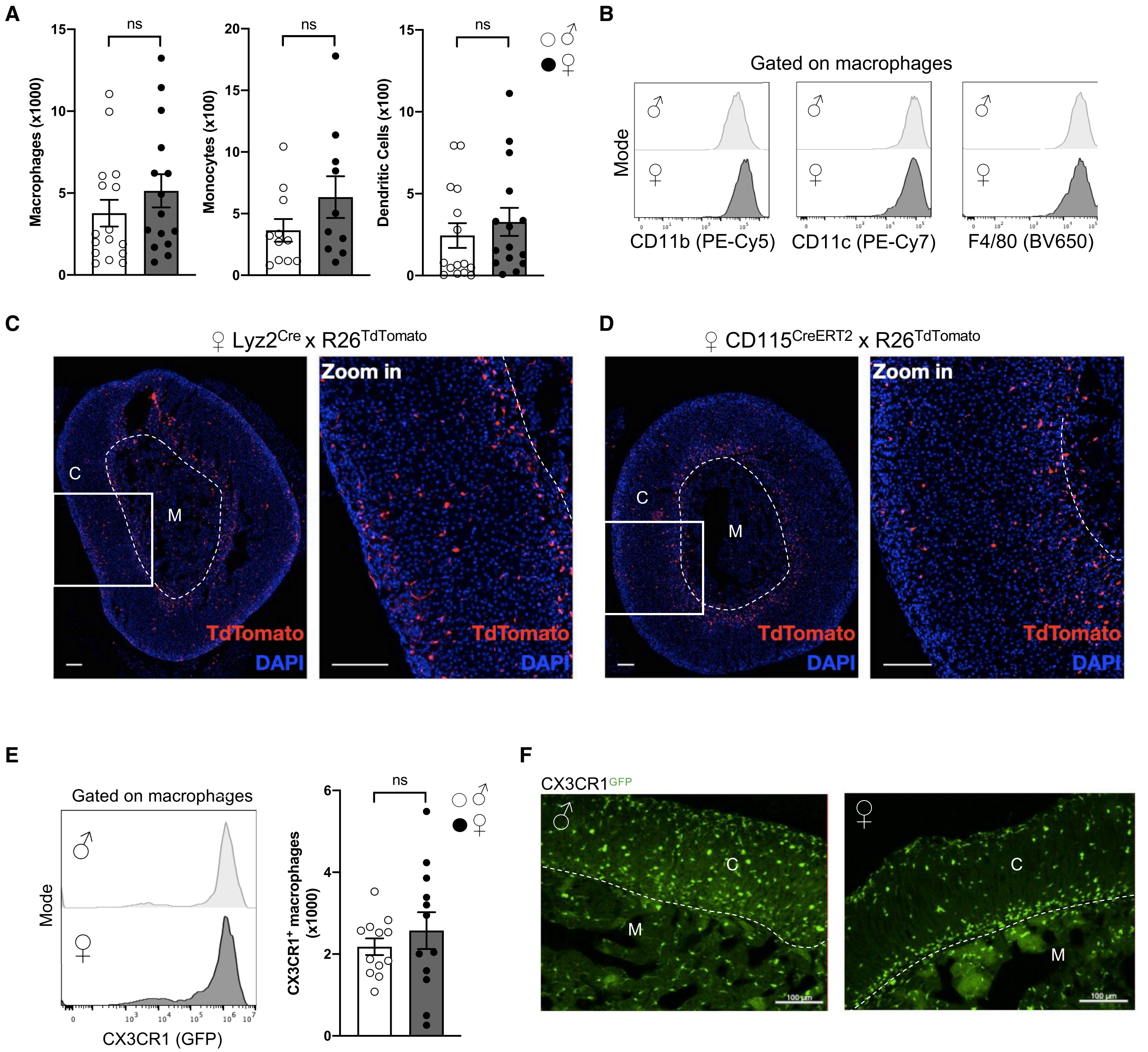

Figure 1. Macrophages are the main adrenal gland immune subset and possess a sex-specific localization

(A) Quantification of macrophages, monocytes, and DCs in the adrenal glands of 7-week-old male and female wild-type mice. Macrophages: ♂ n = 16 and ♀ n = 15. Monocytes: ♂ n = 11 and ♀ n = 10. DCs: ♂ and ♀ n = 15. Data pooled from 2 (monocytes) or 3 (macrophages and DCs) independent experiments.

(B) Histograms representing CD11b, CD11c, and F4/80 expression on male and female AGMs. Data are representative of at least 4 independent experiments.

(C and D) Representative images of R26^{TdTomato} expression in adrenal glands from 10- to 12-week-old female Lyz2^{cre} (C) and CD115^{creERT2} (D) mice 24 h after TAM administration. Scale bars: 100 μm. Data are from one experiment.

(E) Flow-cytometry analysis of CX3CR1^{GFP} expression in male and female AGMs. ♂ n = 12 and ♀ n = 12. Data are pooled from 3 independent experiments.

(F) Microscopy analysis of CX3CR1^{+} cells localization in 8-week-old male and female (nulliparous) CX3CR1$^{GFP/+}$ mice. Scale bar: 100 μm. Data are representative of at least 4 independent experiments. Two-tailed Mann-Whitney tests were used for statistical analysis.

See also Figure S1 and Table S1.

(Figure S2E). We identified two major macrophage clusters that were characterized by high or low MHC class II expression (clusters 2 and 3, respectively). Importantly, the proportion of macrophage subsets was different between female and male mice, with a specific enrichment of MHC class II^{low} macrophages in females, while MHC class II^{high} macrophages were more abundant in males (Figure 2B). Two additional macrophage populations expressing Lyve1 (cluster 4) or Aldh1a2 (cluster 5) were present in both female and male mice (Figure 2A). We observed that myeloid cells presented a highly conserved signature between females and males, with only a few genes being differentially expressed between sexes (Table S1). Among those,

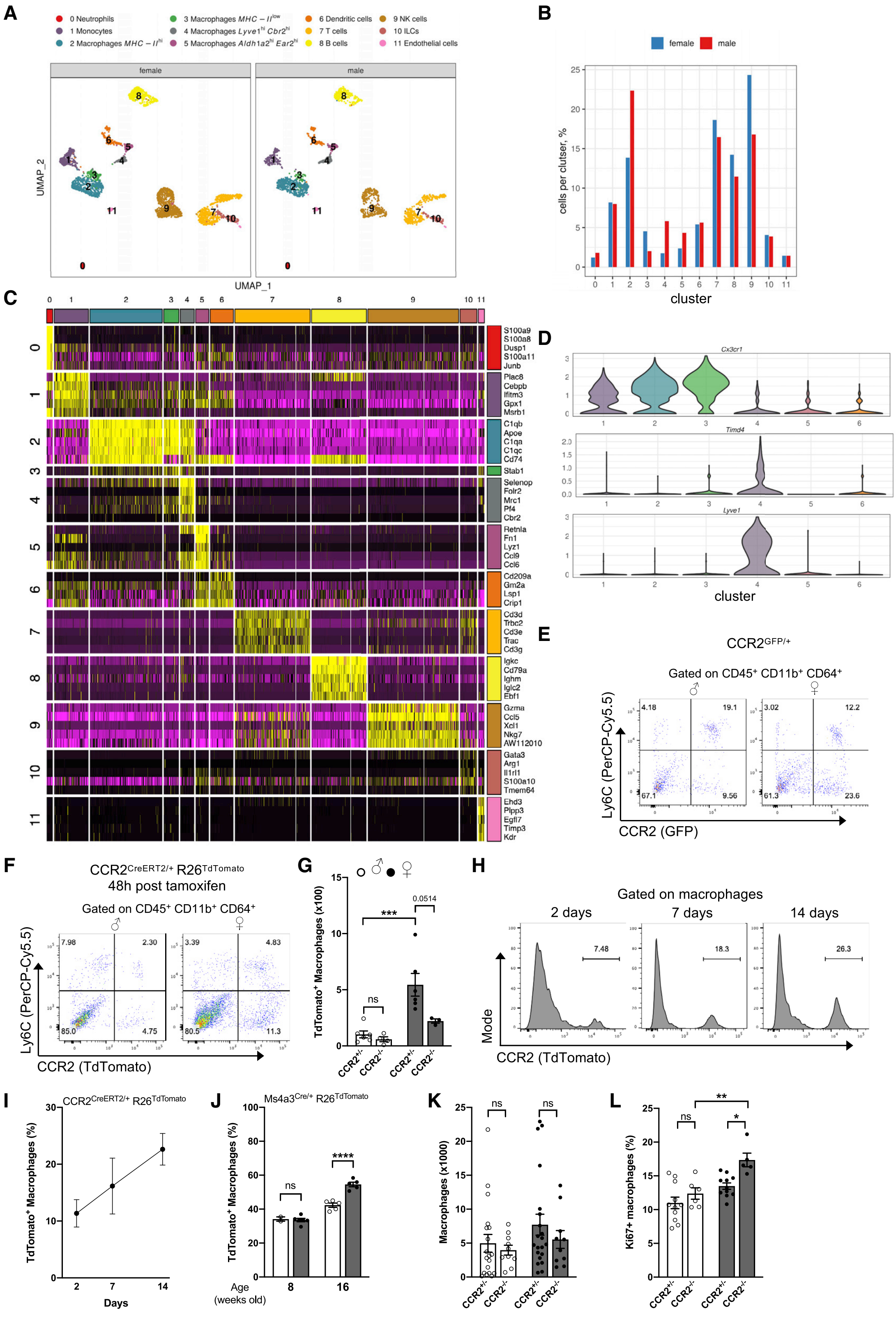

(legend on next page)

Ly6A (Sca1) and ApoC2 mRNAs were highly expressed in female macrophages compared with their male counterparts (Figure S2F). Flow-cytometry analysis of surface Sca1 protein distribution confirmed a higher expression on AGMs isolated from female mice when compared with males (Figure S2G). Among the four macrophage clusters, we found a transcriptional diversity, suggesting a particular developmental origin, localization, or function for each cluster (Figure 2C; Data S1 and S2). The macrophage clusters 2 and 3 expressed *Cx3cr1* mRNA, while clusters 4 and 5 appeared to have lower *Cx3cr1* expression (Figure 2D). Of interest, *Lyve1* and *Timd4* mRNA expression were restricted to macrophage cluster 4 (Figure 2D).

Tissue-resident macrophage numbers are regulated by monocyte recruitment from the blood circulation or local self-proliferation (Ginhoux and Guilliams, 2016). Thus, we next sought to determine the contribution of monocytes to AGM pool size. For this purpose, we took advantage of CCR2^{gfp}-reporter mice. CCR2 is a chemokine receptor highly expressed on monocytes and involved both in their export from bone marrow to blood and in their recruitment to tissues. We found that the population of adrenal gland CD11b^{+}CD64^{+} cells, containing both monocytes and macrophages, comprised numerous CCR2^{+} cells in both sexes (Figure 2E). These cells were detected both in the monocyte (Ly6C^{high}) and the macrophage (Ly6C^{low}) populations (Figure 2E). This observation was further supported by our scRNA-seq data, where *Ccr2* mRNA expression was detected both in monocyte and macrophage clusters (Figure S2H). This result suggested that monocytes might be constantly recruited into adrenal glands, in which they differentiate into macrophages. To evaluate monocyte contribution to the AGM pool size, we performed a short-term fate-mapping experiment. For this purpose, CCR2^{creERT2} x R26^{TdTomato} mice were administered with tamoxifen (TAM), and we analyzed adrenal glands 48 h later. We detected a large fraction of R26$^{TdTomato+}$ cells in adrenal glands, demonstrating that monocyte recruitment plays a central role to maintain macrophage pool homeostasis (Figure 2F). Importantly, monocyte recruitment was decreased in CCR2-deficient female mice (Figure 2G). Furthermore, we found that monocyte recruitment to adrenal glands was more robust in females when compared with age-matched males (Figures 2F and 2G). To determine monocyte fate in adrenal glands, we next performed time course pulse-chase experiments where CCR2^{creERT2} x R26^{TdTomato} mice were administered with TAM and analyzed 2, 7, and 14 days later. Two days post TAM injection, around 10% of AGMs were R26$^{TdTomato+}$ (Figures 2H and 2I). At days 7 and 14, the percentage of R26$^{TdTomato+}$ macrophages slowly and progressively increased to nearly 20% of the total macrophage population (Figures 2H and 2I). To confirm the contribution of monocytes to AGM pool size, we took advantage of Ms4a3^{cre} x R26^{TdTomato} mice (Liu et al., 2019). Ms4a3 is specifically and transiently expressed by monocyte precursors in the bone marrow and absent from tissue macrophages. It therefore could cope with potential direct labeling of tissue macrophages in CCR2 fate mapping. In this genetic model, monocyte-derived cells are labeled, and their numbers can be estimated in tissues. The relative abundance of TdTomato^{+} macrophages was similar in 8-week-old male and female mice (Figure 2J). However, in older 16-week-old animals, the percentage of TdTomato^{+} macrophages was significantly increased in female mice compared with in males (Figure 2J). This result further supports our observation that monocyte recruitment to adrenal glands is more robust in females than males. Finally, in order to determine whether monocyte recruitment is mandatory to sustain AGM populations, we quantified macrophages in CCR2-deficient mice, which have severely reduced blood monocyte counts (Serbina and Pamer, 2006). We observed that macrophage counts remained similar in CCR2-sufficient and -deficient mice (Figure 2K). This suggests that a compensatory mechanism occurs to ensure the maintenance of tissue macrophage density in the absence of optimal monocyte recruitment. These data suggest that monocyte recruitment plays an important role in the maintenance of AGM

Figure 2. scRNA-seq analysis reveals adrenal gland leukocyte diversity and monocyte contribution to the macrophage pool

(A) scRNA-seq analysis of adrenal gland CD45^{+} cells from 7-week-old male and female wild-type mice.

(B) Proportion of each cluster identified in scRNA-seq analysis.

(C) Heatmap showing normalized expression levels of cluster-specific genes.

(D) Violin plots showing *Cx3cr1*, *Timd4*, and *Lyve1* expression by cells from clusters 1–6.

(E) Flow-cytometry plot showing Ly6C and CCR2^{GFP} expression among adrenal gland CD45^{+}CD11b^{+}CD64^{+} cells in male and female CCR2$^{GFP/+}$ mice. Data are representative of three independent experiments.

(F) Flow-cytometry plot showing Ly6C and TdTomato expression among adrenal gland CD45^{+}CD11b^{+}CD64^{+} cells from male and female CCR2^{creERT2} x R26^{TdTomato} mice 48 h after TAM gavage. Data are representative of two independent experiments.

(G) Quantification of TdTomato^{+} macrophages in 16- to 20-week-old male and female heterozygous (CCR2$^{+/-}$, ♂ n = 6 and ♀ n = 6) or double knockin (CCR2$^{-/-}$, ♂ n = 4 and ♀ n = 3) CCR2^{creERT2} x R26^{TdTomato} mice 48 h after TAM gavage. Data are pooled from two independent experiments.

(H) Histograms representing R26^{TdTomato} expression in AGMs from 10-week-old female CCR2$^{creERT2/+}$ x R26^{TdTomato} mice 2, 7, and 14 days after TAM gavage. Data are representative of one (days 7 and 14) or two (day 2) experiments.

(I) Proportions of TdTomato^{+} macrophages from 10-week-old female CCR2$^{creERT2/+}$ x R26^{TdTomato} mice 2 (n = 8), 7 (n = 4), and 14 (n = 3) days after TAM gavage. Data from one (days 7 and 14) or two (day 2) experiments.

(J) Proportions of TdTomato^{+} macrophages from 8 (♂ n = 2, ♀ n = 6) or 16 (♂ n = 5, ♀ n = 5)-week-old male and female Ms4a3$^{cre/+}$ x R26^{TdTomato} mice. Data are from one experiment.

(K) Quantification of AGMs in male and female CCR2$^{+/-}$ (♂ n = 18, ♀ n = 21) and CCR2$^{-/-}$ (♂ n = 10, ♀ n = 10) mice. Data are pooled from four independent experiments.

(L) Proportions of Ki67^{+} AGMs in male and female CCR2$^{+/-}$ (♂ n = 11, ♀ n = 11) and CCR2$^{-/-}$ (♂ n = 6, ♀ n = 5) mice. Data are pooled from two independent experiments.

Statistical analysis was performed using two-way ANOVA with Bonferroni's post-test.

See also Figure S2.

pool size but also sheds light on the existence of alternative mechanism(s) that actively contribute to this process. Embryonically seeded macrophages are renewing through self-proliferation in adults. Our scRNA-seq data indicated that few macrophages were proliferating in adrenal glands as reflected by low *Mki67*, *Ccna2*, and *Top2a* mRNA expression (Figure S2I; data not shown). A recent study reported that peritoneal macrophages show a sex-specific proliferation rate (Bain et al., 2020). Therefore, we assessed the macrophage proliferating rate in male and female CCR2-sufficient and -deficient mice using intracellular Ki67 staining. Flow-cytometry analysis revealed a similar proliferation rate between male and female CCR2-sufficient mice (Figure 2L). However, loss of CCR2 led to increased macrophage proliferation in female mice, while this was not the case in CCR2-deficient males (Figure 2L). Together, these results show that both monocyte recruitment and self-proliferation contribute to the maintenance of AGM pool size in a sex-specific manner.

Embryonic and monocyte-derived AGMs co-exist at homeostasis

Our flow-cytometry data demonstrated that most (around 90%) of AGMs expressed CX3CR1 (Figure 1E). Macrophage *Cx3cr1* expression appeared restricted to clusters 2 and 3 in our scRNA-seq data (Figure 2D). To address the mechanisms governing AGM maintenance, we analyzed CCR2^{creERT2} x R26^{TdTomato} x CX3CR1^{GFP} mice 48 h post TAM administration. CCR2^{+} cells were found to preferentially give rise to CX3CR1^{+} macrophages upon their entry into adrenal glands (Figure 3A). Indeed, 48 h post TAM injection, 80% of R26$^{TdTomato+}$ macrophages were CX3CR1^{GFP} positive (Figure 3A). To determine whether CX3CR1^{+} monocyte-derived macrophages could give rise to CX3CR1^{-} macrophages, we took advantage of double reporter CX3CR1$^{creERT2/GFP}$R26^{TdTomato} mice, allowing us to assess present and past CX3CR1 expression. We found that more than 80% of AGMs were TdTomato^{+}GFP^{+} 2 days after TAM administration (Figure 3B). GFP^{+}R26$^{TdTomato-}$ and GFP^{-}R26$^{TdTomato+}$ cells were virtually absent at this time point (Figure 3B). This set of data indicated that 48 h after TAM administration, CX3CR1^{+} cells were not giving rise to CX3CR1^{low} macrophages (Figure 3B). Furthermore, even 7 days after TAM injection, we still detected approximately 80% of R26$^{TdTomato+}$ GFP^{+} macrophages (Figure 3C). Yet, a population of newly recruited monocyte-derived cells (GFP^{+} R26$^{TdTomato-}$) was observed (Figure 3C). However, we did not observe the appearance and accumulation of GFP^{-}R26$^{TdTomato+}$ cells (Figure 3C). We therefore concluded that CX3CR1^{+} cells are not precursors of CX3CR1^{-} macrophages in adults. We next characterized the phenotype and origin of CXC3R1^{-} AGMs. Our scRNA-seq data pointed out that cells from cluster 4, which are a part of CX3CR1^{-} macrophages, expressed *Timd4* and *Lyve1* mRNA (Figure 2D). We confirmed the presence of Lyve1^{+}Timd4^{+} macrophages in female and male mice using flow cytometry, even though these cells represented a minor subpopulation (Figure S3A). Timd4 expression was higher on Lyve1^{+} macrophages compared with Lyve1^{-} cells in both female and male mice (Figure S3B). Moreover, we confirmed that Timd4^{+} macrophages were CX3CR1^{-} (Figure 3D). Lyve1 and Timd4 are markers associated with an embryonic macrophage origin (Dick et al., 2019). We therefore hypothesized that embryonic and monocyte-derived macrophages could co-exist in adrenal glands.

A previous report showed that populations of embryonically seeded macrophages express CX3CR1 during development but lose this expression in adulthood (Yona et al., 2013). To investigate the developmental origin of AGMs, we performed embryonic pulse-chase experiments. Pregnant CX3CR1^{creERT2} x R26^{TdTomato} mice were injected with TAM at embryonic day 14.5, and their progeny was analyzed at the adult stage (8 weeks old) (Figure 3E). Approximatively 15% of AGMs were labeled, suggesting an embryonic seeding for these cells (Figure 3E). The percentage of R26$^{TdTomato+}$ cells was similar in male and female mice, suggesting that embryonic seeding was not following a sex-specific pattern (Figure 3E). In a similar set of experiments, we observed by fluorescence microscopy R26$^{TdTomato+}$ cells in adult adrenal glands, further demonstrating the presence of embryonically seeded macrophages (Figure 3F). Flow-cytometry analysis identified the presence of macrophages in embryos, and many of these cells were Timd4^{+} (Figure 3G). These data are consistent with recent reports demonstrating that Timd4 is a marker of embryonically derived macrophages (Bain et al., 2020; Dick et al., 2019; Sakai et al., 2019). Interestingly, Lyve1^{+} macrophages from cluster 4 showed high expression of Mrc1 (CD206), a marker associated with macrophage alternative polarization (Figure S3C). Consistent with our observations using Timd4 staining, Lyve1^{+}CD206^{+} cells represented the major macrophage subset in juvenile animals but only a minor population in adults (Figure S3C). We next performed a separate embryonic labeling experiment where mice were sacrificed at postnatal day 7 (P7) following embryonic labeling at embryonic day 14.5 (E14.5) to determine whether embryonic TdTomato^{+} macrophages expressed Lyve1 and Timd4. We observed that >80% of TdTomato^{+} cells expressed both Lyve1 and Timd4, confirming the embryonic origin of cells from cluster 4 (Figure 3H). Importantly, at this early stage, more than 60% of macrophages were TdTomato^{+} (Figure 3H) compared with 15% in 7-week-old mice, suggesting that embryonically seeded AGMs may be replaced by monocyte-derived cells over time.

Low MHC class II expression defines a female-specific AGM subset with restricted sub-tissular distribution

A major difference in macrophage subsets was related to their expression of genes related to the MHC class II. This allowed the identification of MHC class II^{high} and class II^{low} macrophages (clusters 2 and 3, respectively) (Figure 2A). MHC class II^{low} macrophages were characterized by low expression of H2-Aa, CD74, and Ciita mRNA (Figure 4A). Expression of the KEGG "Antigen processing and presentation" pathway appeared higher on MHC class II^{high} macrophages (Figure 4A). The cluster of MHC class II^{low} macrophages was enriched in females when compared with males (Figure 2A). Flow-cytometry analysis confirmed that MHC class II expression on AGMs displayed a well-defined sex-specific pattern. We observed that the MHC class II^{high} population represented about 90% of AGMs in adult males but only 70% of macrophages in females (Figure 4B). While the number of MHC class II^{high} macrophages was comparable between male and female mice, the number of MHC class II^{low} macrophages was about 5-fold higher in females when

CellPress
OPEN ACCESS

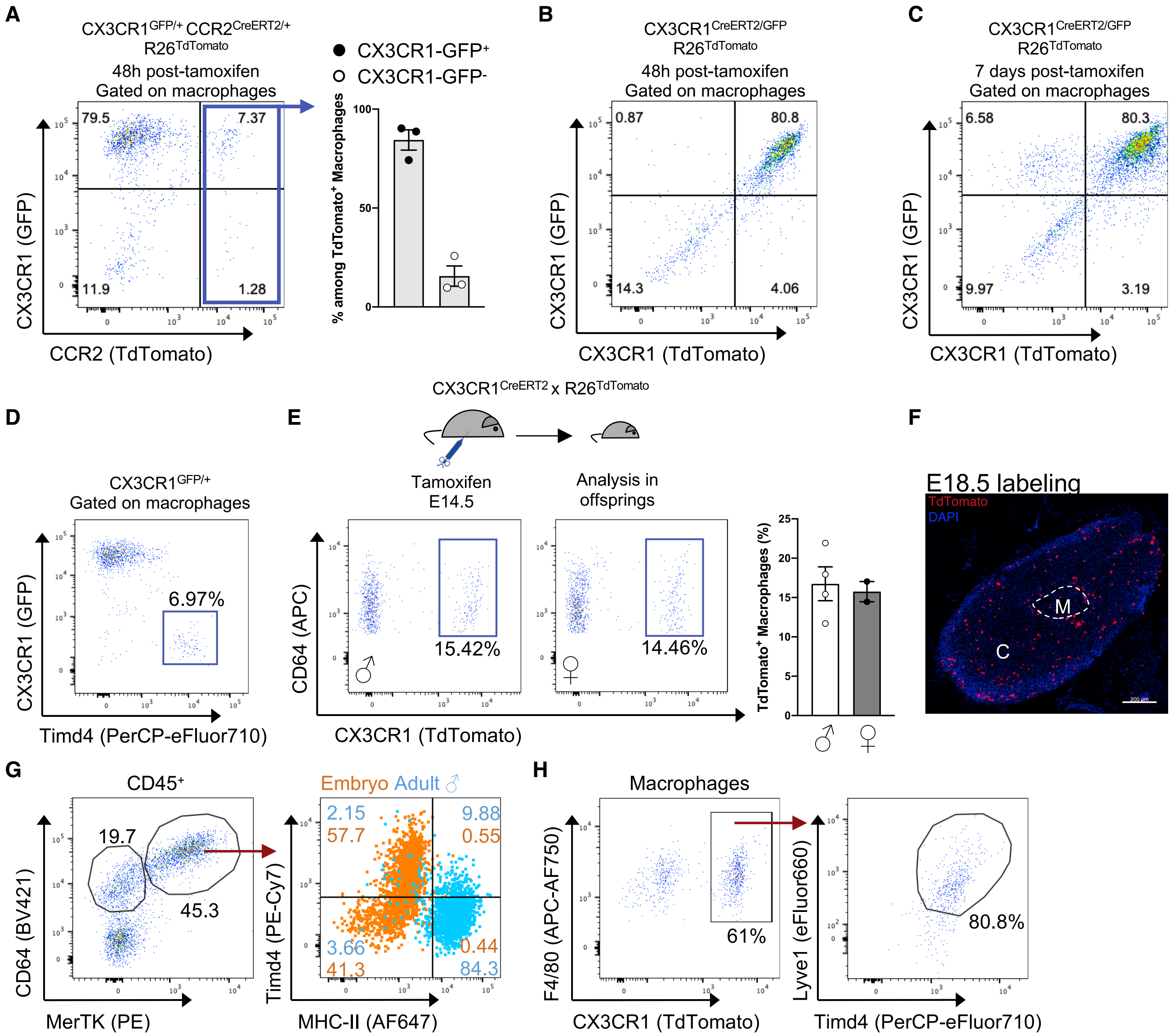

Figure 3. Embryonic and monocyte-derived adrenal gland macrophages are distinct subsets identified through CX3CR1 expression

(A) (Left) Representative plot of macrophage CX3CR1-GFP and R26^{TdTomato} expression and (right) proportions of CX3CR1-GFP^{+} and CX3CR1-GFP^{-} cells among R26$^{TdTomato+}$ AGMs from (n = 3) female CX3CR1$^{GFP/+}$ CCR2$^{CreERT2/+}$ R26^{TdTomato} mice 48 h post TAM gavage. Data are from one experiment.

(B) Representative plot of macrophage CX3CR1 and R26^{TdTomato} expression in double reporter CX3CR1$^{creERT2/GFP}$ R26^{TdTomato} mice 48 h post TAM administration. Data are from one experiment.

(C) Representative plot of macrophage CX3CR1 and R26^{TdTomato} expression in double reporter CX3CR1$^{creERT2/GFP}$ R26^{TdTomato} mice 7 days post tamoxifen administration. Data are from one experiment.

(D) Flow-cytometry plot showing Timd4 and CX3CR1 expression by AGMs. Data are representative of at least 4 independent experiments.

(E) Embryonic labeling of CX3CR1^{creERT2} R26^{TdTomato} mice was performed at E14.5. R26$^{TdTomato+}$ cells were identified in 8-week-old male (n = 4) and female (n = 2) offspring. Data are representative of 2 independent experiments.

(F) Embryonic labeling of CX3CR1^{creERT2} R26^{TdTomato} mice was performed at E18.5. R26$^{TdTomato+}$ cells were identified in 10-week-old female offspring. Scale bar: 200 μm. Data are representative of 2 independent experiments.

(G) Flow-cytometry analysis of Timd4 and MHC class II expression in AGMs from E18–E20 embryos and male adult (9-week-old) mice. Data are from one experiment.

(H) Embryonic labeling of CX3CR1^{creERT2} R26^{TdTomato} mice was performed at E14.5. R26$^{TdTomato+}$ cells were identified in 1-week-old male offspring (left panel), which comprised mainly Tidm4^{+} Lyve1^{+} cells (right panel). Data are representative of n = 2 mice. Data are from one experiment. Two-tailed Mann-Whitney tests were used for statistical analysis.

See also Figure S3.

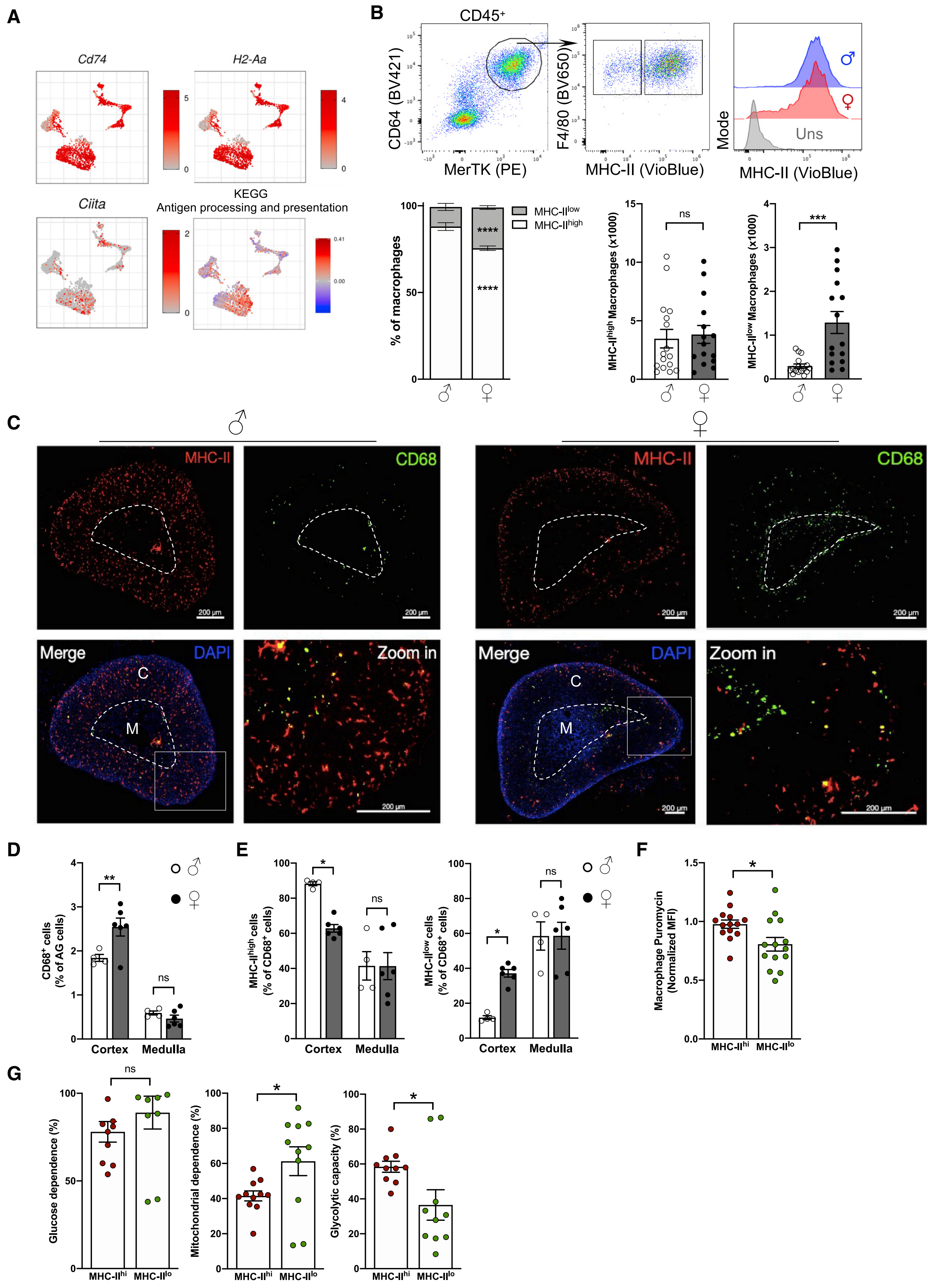

(legend on next page)

compared with age-matched males (Figure 4B). Thus, we next investigated how MHC class II^{high} and class II^{low} macrophages were related to the CX3CR1^{+} and CX3CR1^{-} macrophage subsets. CX3CR1^{+} macrophages were enriched among MHC class II^{high} macrophages compared with the MHC class II^{low} subset (Figure S3D). Moreover, CX3CR1^{+} cells were more frequent among female MHC class II^{low} macrophages compared with their male counterparts (Figure S3D). Timd4 and Lyve1 expressions were higher on MHC class II^{low} macrophages, suggesting that this subset may be enriched in embryonically derived macrophages (Figure S3E). However, MHC class II^{low} macrophages were more numerous in females compared with males, and this was independently of their Timd4 and Lyve1 expression (Figure S3F). Flow-cytometry analysis revealed that CD11c expression was higher in MHC class II^{high} macrophages compared with the MHC class II^{low} subset in both males and females (Figure S3G). Similar to CX3CR1, CD11c expression was higher in female MHC class II^{low} macrophages compared with their male counterparts, further supporting the sex-dimorphic nature of the MHC class II^{low} subset (Figure S3G). Unsupervised spectral flow-cytometry analysis confirmed that AGMs display a sex-specific expression of MHC class II and CX3CR1 (Figures S3H and S3I). Because we observed that the pathway "Antigen processing and presentation" appeared specific for MHC class II^{high} macrophages (Figure 4A), we measured the expression of proteins involved in antigen presentation. CD86 expression was higher on MHC class II^{high} macrophages compared with their MHC class II^{low} counterparts, and CD40 expression tended to follow the same pattern (Figure S3J). However, the markers CD80 and ICOSL appeared highly expressed in female MHC class II^{low} macrophages (Figure S3J). These results suggest that AGM subsets may have a singular antigen presentation potential. Importantly, sexually dimorphic features appear restrained to MHC class II^{low} macrophages, which stand out as a female-enriched population.

To determine whether macrophage subsets displayed a sex-specific distribution across the cortex and medulla, we performed fluorescence microscopy analysis. Pan-macrophage CD68 staining revealed that most macrophages are nested in the cortex rather than the medulla both in female and male mice (Figure 4C). Additionally, we found an enrichment of CD68^{+} cells in the cortex of females compared with in males (Figure 4D). MHC class II staining demonstrated that most cortical macrophages are MHC class II^{high} in male animals (Figure 4E). This percentage was significantly lower in female mice, which had increased proportion of MHC class II^{low} cells in their adrenal gland cortex (Figure 4E). Interestingly, MHC class II^{low} CD68^{+} cells were enriched at the border between the cortex and the medulla in female mice (Figure 4C). This phenotype was also observed using F4/80 staining, further indicating that these cells are macrophages (Figure S3K). Our data did not reveal a sexual dimorphism in the distribution of MHC class II^{high} and class II^{low} macrophages in the adrenal gland medulla (Figure 4E). Thus, the sex dimorphism in macrophage MHC class II expression identified by flow cytometry and fluorescence microscopy appears restricted to the adrenal gland cortex and, in particular, to a zone between the cortex and the medulla. Finally, we assessed the metabolic configuration of adrenal gland MHC class II^{high} and class II^{low} macrophages using the recently described SCENITH method (Arguello et al., 2020). MHC class II^{high} macrophages appeared to have more active metabolism as illustrated by increased puromycin integration when compared with MHC class II^{low} macrophages (Figure 4F). The glucose dependence was similar between both macrophage subsets (Figure 4G). However, MHC class II^{low} macrophages displayed higher mitochondrial dependence, while MHC class II^{high} macrophages were characterized by increased glycolytic capacity (Figure 4G). Thus, MHC class II^{high} and class II^{low} AGMs relied on different metabolic pathways at steady state.

MHC class II^{low} macrophages locate within the X-zone and are tied to its maintenance

Since MHC class II^{low} macrophages appeared to be specifically located at the border between the cortex and the medulla, we asked whether this population may be associated with the X-zone, a cortical zone that regresses along organ maturation in males (Gannon et al., 2019). To address this question, we compared young mice, in which the X-zone is intact, and adults in which the sex-specific organ structure is already established. Flow-cytometry analysis demonstrated that the majority (around 75%) of AGMs in juvenile 1-week-old mice stained positive for Lyve1 but not for MHC class II (Figure 5A). MHC class II^{high} macrophages became the predominant subset in adulthood (Figure 5A). Importantly, we did not observe sex dimorphism in 1- and 3-week-old animals, while it was clearly established in 7-week-old mice (Figure 5B). These results suggested that sex

Figure 4. MHC class II^{low} macrophages are a female-specific subset with restricted localization

(A) scRNA-seq analysis of *CD74*, *H2-Aa*, *Ciita*, and the KEGG pathway "Antigen processing and presentation" expression among myeloid cells.

(B) (Top) Flow-cytometry plots showing F4/80 and MHC class II expression among AGMs from 7-week-old male and female mice. (Bottom) Proportions and numbers of MHC class II^{high} and class II^{low} AGMs from 7-week-old male and female wild-type mice. ♂ n = 16 and ♀ n = 15. Data are pooled from 3 independent experiments.

(C) Fluorescence-microscopy analysis of CD68 and MHC class II expression in adrenal glands from 7-week-old male and female mice. Scale bar: 200 μm. M, medulla; C, cortex. Data are representative of at least 3 independent experiments.

(D) Distribution of CD68^{+} cells between cortex and medulla from adrenal glands of 7-week-old male and female mice. Data are represented as proportion of CD68^{+} cells from each zone among total cells. ♂ n = 4 and ♀ n = 6. Quantification from one experiment.

(E) Proportion of MHC-II^{high} and MHC-II^{low} CD68^{+} cells in the cortex and medulla of adrenal glands from 7-week-old male and female mice. ♂ n = 4 and ♀ n = 6. Quantification from one experiment.

(F) Analysis of macrophage metabolic activity using SCENITH, represented by Puromycin MFI (n = 14). Data pooled from 4 independent experiments.

(G) Measure of glycolytic and mitochondrial metabolism in macrophages using SCENITH (n = 10–11). Data pooled from 3 independent experiments.

Statistical analysis was performed using two-tailed Mann-Whitney tests (panel B, quantifications), two-way ANOVA with Bonferroni's post-test (proportions in panel B, panel D and panel E), or paired Wilcoxon t-tests (panels F and G). See also Figure S4.

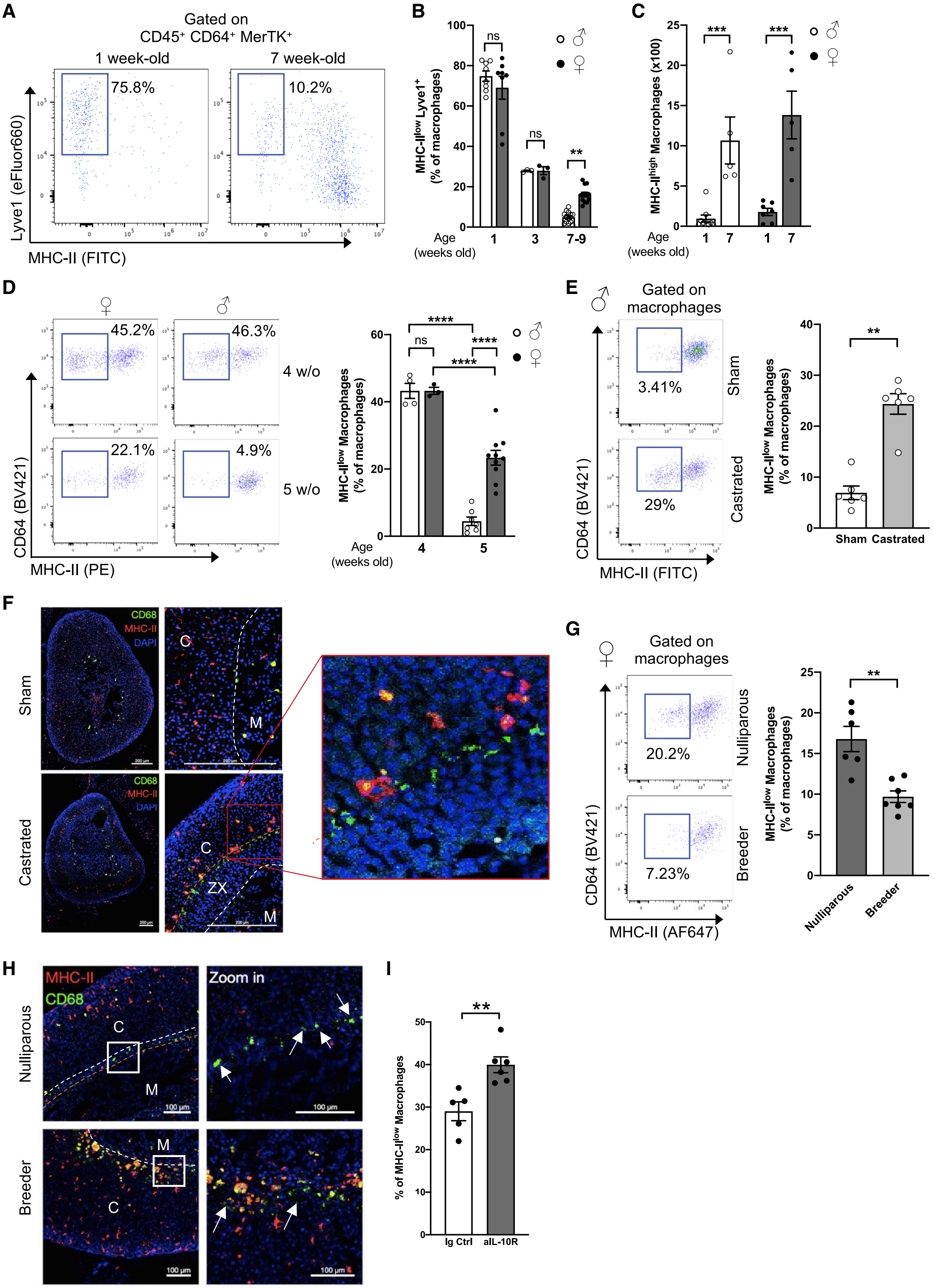

(legend on next page)

dimorphism appears before 7 weeks of age, likely together with puberty onset and X-zone disappearance in males. The high proportions of MHC class II^{low} macrophages in juvenile mice was mirrored by very low numbers of MHC class II^{high} macrophages, while this subset was enriched in 7-week-old mice (Figure 5C). Concomitantly, we measured lower CCL2 levels in adrenal gland homogenates from 3-week-old mice compared with 6- to 7-week-old mice, while their serum CCL2 levels remained similar (Figure S4A). This suggests that adrenal gland maturation is mirrored by increased local CCL2 production, which could trigger accelerated monocyte recruitment.

A recent report demonstrated that sexual hormones, and particularly estrogens, affected the phenotype and functions of peritoneal macrophages (Bain et al., 2020). To determine whether estrogens were responsible for the accumulation of MHC class II^{low} macrophages, 6-week-old female mice were ovariectomized or received a control surgery (sham). As expected, uteri weights were decreased in ovariectomized (OVX) mice compared with sham-operated animals (Figure S4B). We analyzed their adrenal glands 6 weeks post-surgery and found no differences in the numbers of adrenal gland monocytes or macrophages between sham-operated and OVX mice (Figure S4C). The numbers of MHC class II^{high} and class II^{low} macrophages were also unchanged between sham-operated and OVX animals (Figure S4D). Taken together, these data demonstrated that ovaries-derived hormones are unlikely regulators of AGM sex dimorphism.

X-zone regression occurs between P28 and P35 in male mice (Huang and Kang, 2019). Microscopy analysis revealed that the X-zone was present in 4-week-old females but absent in males in our experimental conditions (Figure S4E). We observed that the percentage of MHC class II^{low} macrophages was similar in 4-week-old females and males (Figure 5D). Strikingly, in 5-week-old males, the percentage of MHC class II^{low} macrophages decreased to 5%, while it remained around 20% in females (Figure 5D). These data suggest that X-zone disappearance precedes macrophage sex-dimorphism establishment and correlated with the loss of MHC class II^{low} macrophages in males. To further establish a causal link between X-zone presence and AGM sex dimorphism, males were castrated at 3 weeks of age to prevent X-zone degradation. We analyzed their adrenal glands when the mice were 7-week-old. MHC class II staining demonstrated an increase in the population of MHC class II^{low} macrophages in castrated mice when compared with sham-operated animals (Figure 5E). Interestingly, when we analyzed the localization of AGM, we found that MHC class II^{high} macrophages had a very particular distribution at the border with the X-zone in castrated mice (Figure 5F). In this same location, we found an increased number of MHC class II^{low} macrophages in castrated mice compared with sham-operated controls (Figure 5F). These data demonstrated that X-zone maintenance is sufficient to maintain MHC class II^{low} macrophages in castrated males.

Next, we analyzed adrenal glands from female retired breeders, in which the X-zone was degraded during pregnancy, and age-matched nulliparous controls. MHC class II^{low} macrophage proportions dropped to 10% in retired breeders, similar to what we observed in males, while it was close to 20% in age-matched nulliparous mice (Figure 5G). The distribution pattern of MHC class II^{+} cells in female retired breeders resembled the pattern observed in males (Figure 5H). No sex dimorphism was observed between male and female retired breeders (Figure S4F). Thus, we concluded that X-zone disappearance drives the establishment of sex dimorphism in AGM phenotype and tissue distribution.

We then analyzed the molecular mechanisms controlling MHC class II expression in AGMs. While MHC class II regulation in DCs is well established, relatively little is known about the mechanisms occurring in monocytes and macrophages (Unanue et al., 2016). Interleukin-10 (IL-10) signaling was previously shown to modulate MHC class II expression in monocyte-derived cells (Koppelman et al., 1997). Thus, we injected IL-10R-blocking antibodies (Abs) and analyzed macrophage MHC class II expression 72 h later. Preventing IL-10 receptor signaling in females led to increased proportions of MHC class II^{low} macrophages (Figure 5I). This result suggests that local IL-10

Figure 5. Adrenal gland macrophage sex-dimorphism is established with organ maturation and depends on X-zone presence

(A) Representative plots showing AGM MHC-II and Lyve1 expression in male 1- or 7-week-old mice. Data representative of 2 independent experiments.

(B) Proportions of MHC-II^{low} Lyve1^{+} macrophages in male and female 1-(♂ n = 8, ♀ n = 8), 3-(♂ n = 3, ♀ n = 3) or 7 to 9-week-old (♂ n = 13, ♀ n = 13) mice. Data pooled from 2 independent experiments.

(C) Quantification of MHC-II^{high} AGMs in male and female 1- (♂ n = 9, ♀ n = 7) or 7-week-old (♂ n = 5, ♀ n = 5) mice. Data are pooled from 2 independent experiments.

(D) (Left) Representative plots showing MHC class II expression and (right) proportions of MHC class II^{low} macrophages in male and female 4- (♂ n = 4, ♀ n = 3) and 5-week-old (♂ n = 7, ♀ n = 10) wild-type mice. Data are pooled from 2 independent experiments.

(E) (Left) Representative plots showing MHC class II expression and (right) proportions of MHC class II^{low} AGMs in 7-week-old castrated (n = 6) and sham-operated (n = 6) wild-type male mice. Data are pooled from 2 independent experiments.

(F) Confocal-microscopy analysis of MHC class II and CD68 expression in 7-week-old castrated and sham-operated wild-type mice. The X-zone is comprised between white and orange dots. Scale bar: 200 μm. M, medulla; C, cortex; ZX, X- zone. Data are representative of 2 independent experiments.

(G) (Left) Representative plots showing MHC class II expression and (right) proportions of MHC class II^{low} AGMs in female retired breeders (n = 7) and age-matched nulliparous (n = 6) mice. Data are pooled from 2 independent experiments.

(H) Fluorescence-microscopy analysis of CD68 and MHC class II expression in adrenal glands from 12-week-old female retired breeders and age-matched nulliparous mice. The X zone is comprised between white and orange dots. Scale bar: 100 μm. M, medulla; C, cortex. Data are representative of 2 independent experiments.

(I) Proportions of MHC class II^{low} macrophages in female mice treated with anti-IL-10R-blocking antibody or isotype control. Data are pooled from 3 independent experiments.

Statistical analysis was performed using two-way ANOVA with Bonferroni's post-test (B–D) or two-tailed Mann-Whitney tests (E, G, and I).

See also Figure S5.

production may drive the zone-restricted macrophage phenotype observed on MHC class II expression. Further investigation is required to define the precise mechanisms involved in this process and whether IL-10 controls MHC class II export, recycling, or degradation in AGMs.

AGMs impact local lipid homeostasis

Finally, we decided to inquire about the functions of AGMs. We investigated whether AGMs could sample blood-borne particulate material, a function reported in adipose tissue, intestinal, and lung macrophages (Silva et al., 2019). We injected intravenous (i.v.) tetramethylrhodamine (TRITC)-conjugated dextran (65–85 kDa) and compared perigonadal white adipose tissue (WAT) macrophages, used as a positive control, and AGMs 20 min post-injection. As expected, WAT macrophages stained positive for dextran, confirming their access to blood-derived particles (Figure S5A). However, AGMs remained negative for TRITC-dextran, suggesting that these cells did not access blood-borne particles rapidly (Figure S5A). This was the case both in female and male mice (Figure S5A). Our data does not exclude the possibility that AGMs might access blood material with a slower rate when compared with WAT macrophages. To gain further insight into AGM morphology in their native environment, we performed electron-microscopy analysis of adrenal glands. Macrophage presence in adrenal glands was sparse. Cells with a macrophage morphology were observed, and their surrounding cells contained lipid droplets (Figure S5B). Electron-microscopy analysis of purified macrophages showed that, compared with peritoneal macrophages, AGMs were enriched in structures resembling lipid droplets (Figure S5C, red arrows). This suggests that AGMs might uptake material, likely lipid derivatives, produced and released by neighboring cells and possibly dead cells and thus contribute to tissue lipid homeostasis. We next investigated whether AGMs might be involved in the control of norepinephrine (NE) release, a hormone produced by the adrenal gland medulla. Our scRNA-seq data indicated that AGMs express enzymes involved in NE degradation, namely monoamine oxidase (*Maoa*) and *Comt* (Figure S5D). *Maoa* expression was low in adrenal gland immune cells with only few macrophages possessing detectable *Maoa* mRNA (Figure S5D). *Comt* expression was detected in several populations of adrenal gland immune cells (Figure S5D). In our dataset, we did not detect the expression of enzymes involved in NE synthesis in immune cells (*Th*, *Ddc*, and *Dbh*) (data not shown). To investigate whether AGMs can degrade NE, we sorted $CD64^{+}MerTK^{+}$ cells and incubated them overnight in the presence of NE and clorgyline, a selective Maoa pharmacological inhibitor. Clorgyline addition did not impact on the concentration of NE detected in our experimental setup (Figure S5D). Thus, we concluded that AGMs, at least *ex vivo*, have a limited ability to degrade NE through Maoa (Figure S5D). This might be due to the sorting of the whole population of AGMs, thus diluting the population of medulla-resident macrophages for which a specific marker is yet to be identified and validated.

Next, we decided to investigate the growth-factor dependence of AGMs. Macrophages rely on macrophage colony-stimulating factor (M-CSF) and/or granulocyte M-CSF (GM-CSF) for their survival in peripheral tissues (Cecchini et al., 1994; Dai et al., 2002; Guilliams et al., 2013; Nishinakamura et al., 1996; Pridans et al., 2018). CSF1 is produced by several cell types including fibroblasts and mesothelial cells (Bellomo et al., 2020; Ivanov et al., 2019). In adrenal glands, CSF1 is thought to derive from the zona reticularis (Bellomo et al., 2020; Ryan et al., 2001). To decipher AGM dependence on growth factors, we injected a CD115 (CSF1R)-blocking Ab. Compared with isotype control-treated mice, anti-CD115 Ab administration completely depleted AGMs (Figure 6A). Fluorescence-microscopy analysis in $CX3CR1^{GFP+}$ mice, administered with anti-CD115 Ab, confirmed the complete disappearance of AGMs (Figure 6B). These data demonstrated that AGMs depend on CSF1R for their survival. Tissue analysis did not show a major alteration of adrenal gland morphology in macrophage-depleted mice (Figure S5E). Serum aldosterone and corticosterone levels remained similar between macrophage-depleted and control mice, suggesting that macrophages are not involved in the systemic control of these hormone levels at steady state (Figure S5F). Additionally, serum potassium concentration, a surrogate readout for aldosterone pathway activation, remained similar in control and macrophage-depleted mice (Figure S5G).

To further investigate the contribution of AGMs to tissue homeostasis, we performed RNA-seq analysis of adrenal glands extracted from female and male mice treated with isotype control or with anti-CD115 Ab. We identified a well-defined transcriptomic signature associated with macrophage depletion in both sexes (Figure S6A). Among the top differentially regulated genes, we observed multiple macrophage-associated genes (CX3CR1, CSF1R, Lyz2, Adgre1), confirming the depletion efficiency in this experiment (Figure S6B). In agreement with our observations that AGMs express MHC class II, and in particular in adult males, macrophage depletion was correlated with decreased expression of transcripts encoding for proteins involved in MHC class II synthesis and regulation (Figure S6C). Macrophage depletion was associated with decreased lipid-associated metabolism in adrenal glands (Figure S6D). To determine how this prediction reflected on adrenal gland morphology and lipid-content distribution, we performed Bodipy staining. Microscopy analysis found that the number of small-size (up to 5 μm^2) $Bodipy^{+}$ particles was increased in macrophage-depleted mice compared with isotype-injected controls (Figures 6C and 6D). The distribution of larger $Bodipy^{+}$ particles was not affected by macrophage removal (Figures 6C and 6D). The precise nature of lipids modulated in macrophage-depleted mice remains to be established.

One of the most regulated genes associated with macrophage depletion in female and male mice was Cybb (Figure S6E). Cybb encodes for a subunit of Nox2 and subsequently impacts reactive oxygen species (ROS) generation. Nox2 was implicated in macrophage ROS generation and microbial killing (Bedard and Krause, 2007). Furthermore, Nox2 is also involved in Cyp11b2-dependent aldosterone generation by adrenal gland cortical cells (Rajamohan et al., 2012). Our data revelated that AGM subsets express high Cybb mRNA levels when compared with other adrenal gland immune cells (Figure S6F). Since macrophage depletion was correlated with a loss of adrenal Cybb mRNA expression, we decided to investigate whether local aldosterone levels were affected in macrophage-depleted adrenal glands.

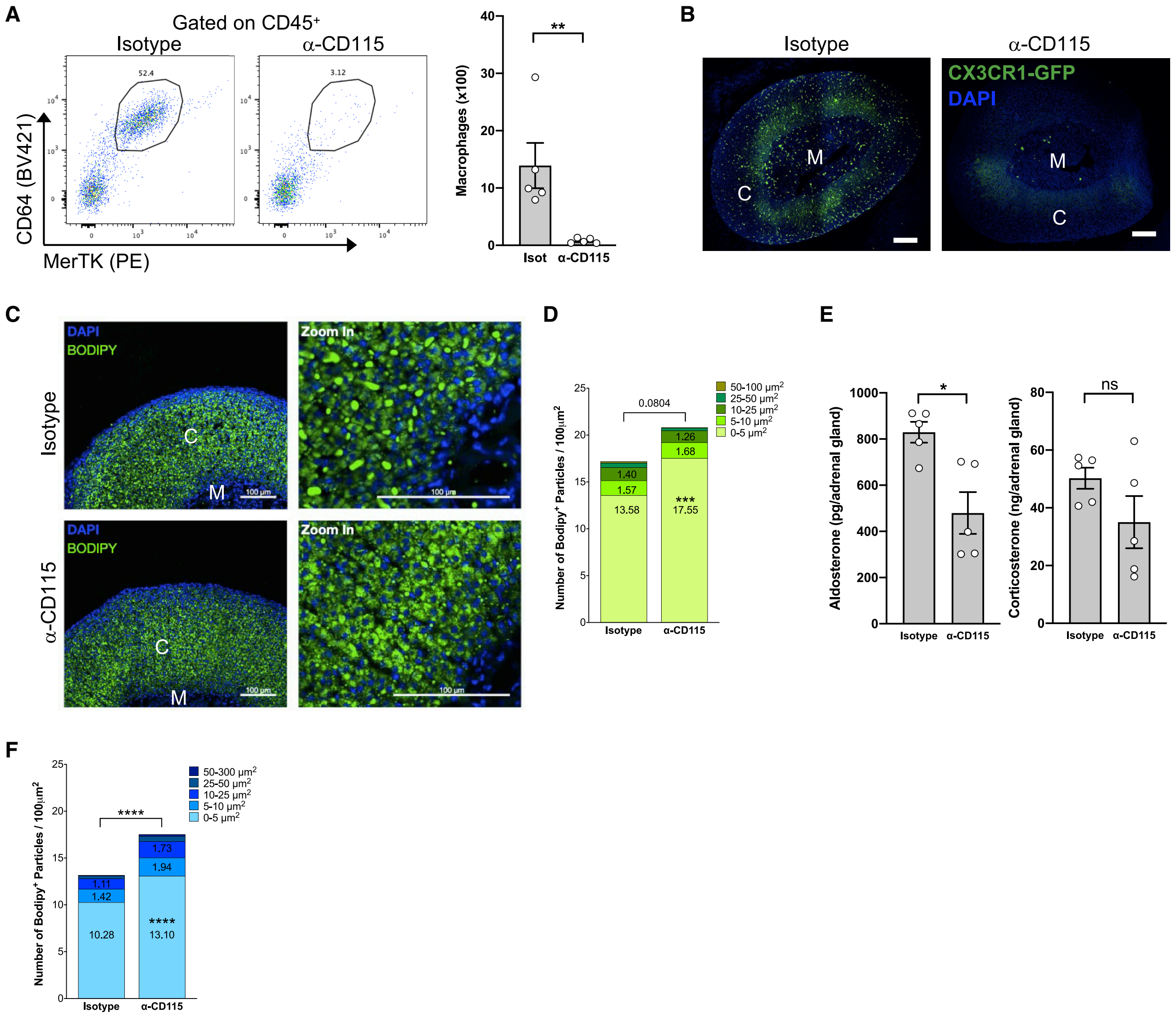

Figure 6. Adrenal gland macrophages control tissue lipid metabolism

(A) (Left) Representative plots and (right) quantification of AGMs in 7- to 8-week-old CX3CR1$^{GFP/+}$ mice after macrophage depletion using α-CD115 (n = 5) or isotype control (n = 5).

(B) Microscopy analysis of adrenal glands from α-CD115- or isotype-control-treated CX3CR1$^{GFP/+}$ mice. One microscopy experiment was performed to confirm depletion efficiency.

(C) Microscopy analysis of adrenal glands from α-CD115- or isotype-control-treated male mice using Bodipy staining. Data are from one experiment.

(D) Quantification of Bodipy$^+$ particles of different sizes in adrenal glands from α-CD115- (n = 3) or isotype-control- (n = 3) treated male mice. Data are from one experiment.

(E) Aldosterone and corticosterone levels in adrenal gland homogenates from 8-week-old α-CD115- (n = 5) or isotype-control- (n = 5) treated female mice submitted to a 12 h cold challenge. Data are from one experiment.

(F) Quantification of Bodipy$^+$ particles of different sizes in adrenal glands from 8-week-old α-CD115- (n = 5) or isotype-control- (n = 5) treated female mice submitted to a 12 h cold challenge.

Statistical analysis was performed using two-way ANOVA with Bonferroni's post-test (D and F) or two-tailed Mann-Whitney tests (A and E).

See also Figure S6.

For this purpose, we housed mice at 4°C for 12 h. Indeed, cold exposure has been shown to trigger a stress response and increased systemic corticosterone concentration (Williams et al., 2017). To assess whether hormone production was modulated by macrophages in cold-challenged mice, we injected isotype control or anti-CD115-blocking Ab, and we evaluated local production and systemic levels of corticosterone and aldosterone. Our data revealed that while corticosterone levels were not altered in the adrenal glands of macrophage-depleted mice, aldosterone concentration was lessened in the absence of

macrophages (Figure 6E). However, this did not translate into decreased systemic aldosterone levels (Figure S6G). Corticosterone serum concentration was also comparable in both experimental groups (Figure S6G). Importantly, Cyp11b2 mRNA expression, a key enzyme in the aldosterone synthesis pathway, was decreased in adrenal glands following macrophage depletion (Figure S6E), further demonstrating macrophage involvement in aldosterone production. Cold challenge further amplified the difference in lipid-particle content between control and macrophage-depleted mice (Figure 6F). Mice treated with anti-CD115 Ab had increased number of Bodipy$^+$ lipid particles compared with isotype-injected animals (Figure 6F). As observed at steady state, the small-size particles were the most affected (Figure 6F). Whether a specific cortical macrophage subset is involved in this mechanism remains to be defined.

DISCUSSION

In the present study, we observed that monocyte recruitment was more important in female mice compared with age-matched male animals. The molecular mechanisms governing this differential monocyte recruitment are yet to be defined. We observed that CCL2 adrenal gland concentration is similar between females and males. Nevertheless, whether additional CCR2 ligands, such as CCL7, are expressed in a sex- and age-dependent manner in adrenal glands and could account for the differential monocyte recruitment remains to be established. Another important question that the current study rose is whether monocyte recruitment occurs in specific adrenal cortex sites and whether these sites differ depending on the sex.

We identified a cluster of embryonically seeded macrophages co-expressing Timd4 and Lyve1. The presence of Lyve1-expressing macrophages was previously documented around large blood vessels (Lim et al., 2018). Perivascular Lyve1$^+$ macrophages contribute to vessel function by interacting with the extracellular matrix (Lim et al., 2018). Lyve1$^+$MHC-II^{low} macrophages have also been identified in lung, adipose tissue, heart, and dermis (Chakarov et al., 2019). Importantly, Lyve1$^+$ Timd4$^+$ macrophages across different tissues have been associated with an embryonic origin, similar to our conclusions in adrenal glands (Dick et al., 2022). These cells were frequently located in close proximity to blood vessels (Chakarov et al., 2019). The tissue localization and precise function of Lyve1$^+$ macrophages in adrenal glands require further investigation. These cells were previously shown to decrease fibrosis development in lung and heart (Chakarov et al., 2019). Whether they modulate fibrosis progression during chronic inflammation or tumor development in adrenal glands is an exciting question that needs to be addressed.

The presence of MHC class II^{low} macrophages was observed in female mice. These cells depended on X-zone maintenance, and its disappearance led to the loss of MHC class II^{low} macrophages. One could speculate that adrenal gland X-zone removal is associated with the generation of peptides that could be loaded and presented on macrophage MHC class II. This could trigger an auto-immune response and generate exacerbated and deleterious inflammation. Thus, a major role of adrenal gland MHC class II^{low} macrophages could be to uptake and degrade antigens avoiding unnecessary local and systemic T cell activation.

AGM depletion was characterized by a dysregulation of tissue lipid metabolism. During stress induction, macrophage depletion resulted in lower aldosterone local production. The very same trend was also observed for corticosterone. Increased aldosterone production leads to hypertension. Thus, controlling adrenal gland aldosterone production and release are critical to prevent hypertension. Our data suggest a role for macrophages in adrenal gland aldosterone generation. However, the administration of anti-CD115 Ab leads to macrophage depletion in multiple tissues (Carrero et al., 2017). Therefore, decreased aldosterone production in adrenal glands might not be a direct consequence of AGM depletion. To test the causal role of AGMs in the control of aldosterone production upon stress, one would need to selectively deplete adrenal macrophages without affecting macrophage populations in other organs. Such models have been established for liver and brain macrophages following extensive analysis of their transcriptome (Buttgereit et al., 2016; Scott et al., 2016). Nevertheless, genetic or pharmacological approaches for selective AGM depletion are yet to be developed and validated. Aldosterone is produced by a specialized layer in the adrenal gland cortex: zona glomerulosa (Zennaro et al., 2020). Among the macrophage subsets present in the adrenal gland cortex, the population involved in aldosterone generation remains to be defined.

Together, our observations define the development and heterogeneity of AGMs in male and female mice. These results lay the foundation for future studies to address the interplay between macrophages and endocrine cells in the adrenal gland and their potential homeostatic or pathological contributions during disease responses and stress-hormone production.

Limitations of the study

The main limitation of the current study is the lack of genetic models allowing to selectively target AGMs for depletion or conditional expression studies. Generating such tools will provide the opportunity to dissect the precise function of AGMs in tissue homeostasis and during stress exposure. Additionally, and due to the very low number of AGM subsets, the analysis of their metabolic configuration in their native microenvironment is challenging.

STAR★METHODS

Detailed methods are provided in the online version of this paper and include the following:

- KEY RESOURCES TABLE
- RESOURCE AVAILABILITY
  - Lead contact
  - Materials availability
  - Data and code availability
- EXPERIMENTAL MODEL AND SUBJECT DETAILS
- METHOD DETAILS
  - Tamoxifen treatments
  - Surgery procedures
  - *In vivo* macrophage depletion
  - *In vivo* IL-10R blockade

- ○ Flow cytometry analysis
- ○ SCENITH
- ○ Dextran uptake assay
- ○ Norepinephrine degradation assay
- ○ ELISA assays
- ○ Bulk RNA-seq
- ○ Quantification of serum cation concentration
- ○ Single-cell RNA-seq data analysis
- ○ Tissue histology
- ○ Immunostaining
- ○ Immunofluorescence quantification method
- ○ Fluorescence microscopy
- ○ Electron microscopy
- QUANTIFICATION AND STATISTICAL ANALYSIS

SUPPLEMENTAL INFORMATION

Supplemental information can be found online at https://doi.org/10.1016/j.celrep.2022.110949.

ACKNOWLEDGMENTS

We would like to thank the C3M animal facility for technical support and the GIS-IBISA multi-sites platform Microscopie Imagerie Côte d'Azur (MICA), and particularly the imaging site of C3M supported by Conseil Régional, Conseil Départemental, and IBISA. We sincerely thank Narges Khedher and Marie Irondelle for their help. We would like to thank Fabien Labbal and Kevin Lebrigand and the UCA Genomix Platform. We acknowledge the flow cytometry facility from the "Institut de Pharmacologie Moléculaire et Cellulaire" part of the MICA GIS IBiSA labeled platform and the UMN flow cytometry core facility. A.G. was supported by the French government through the UCAJedi Investments in the Future projects managed by the National Research Agency (ANR) (ANR-15-IDEX-01). M.M.F. and K.Z. were supported by Government of Russian Federation (grant 08–08). K.-W.K. is supported by NIH (DK126753). R.R.G. is supported by Centre National de la Recherche Scientifique (CNRS). L.Y.-C. is supported by Institut National de la Santé et de la Recherche Médicale (INSERM), Fondation de France (00066474), and the European Research Council (ERC) consolidator program (ERC2016COG724838). J.W.W. was supported by National Institutes of Health (HL138163) and American Heart Association (CDA 855022). S.I. was supported by INSERM and Agence Nationale de la Recherche (ANR-17-CE14-0017-01 and ANR-19-ECVD-0005-01). D.D. was supported by grants from the ANR and the European Union: EGID ANR-10-LABX-46. R.J.A. was supported by ANR-20-CE-CE14-0028-01 and CoPoC MAT-PI-17493-A-04.

AUTHOR CONTRIBUTIONS

S.I., J.W.W., and R.R.G. conceptualized the study. B.D., A.G., Y.X., J.M., A.D., A.C., N.V., S.Q., H.G., M.I.S., P.R.S., S.-H.K., A.Z., J.D., J.G., V.M., P.B., C.D., A.W., C.B.-W., S.P., S.C., K.-W.K., L.Y.-C., R.R.G., J.W.W., and S.I. performed experiments, analyzed the data, and edited the manuscript. B.D., A.G., R.R.G., J.W.W., and S.I. wrote the manuscript. M.M.F. and K.Z. performed the analysis of the scRNA-seq data and bulk RNA-seq data and wrote and edited the manuscript. D.D., B.B., R.J.A., P.R., and F.G. provided tools and expertise and edited the manuscript.

DECLARATION OF INTERESTS

The authors declare no competing interests.

Received: November 9, 2021
Revised: March 10, 2022
Accepted: May 23, 2022
Published: June 14, 2022

REFERENCES

Arguello, R.J., Combes, A.J., Char, R., Gigan, J.P., Baaziz, A.I., Bousiquot, E., Camosseto, V., Samad, B., Tsui, J., Yan, P., et al. (2020). SCENITH: a flow cytometry-based method to functionally profile energy metabolism with single-cell resolution. Cell Metabol. *32*, 1063–1075.e7. https://doi.org/10.1016/j.cmet.2020.11.007.

Audenet, F., Mejean, A., Chartier-Kastler, E., and Roupret, M. (2013). Adrenal tumours are more predominant in females regardless of their histological subtype: a review. World J. Urol. *31*, 1037–1043. https://doi.org/10.1007/s00345-012-1011-1.

Bain, C.C., Bravo-Blas, A., Scott, C.L., Gomez Perdiguero, E., Geissmann, F., Henri, S., Malissen, B., Osborne, L.C., Artis, D., and Mowat, A.M. (2014). Constant replenishment from circulating monocytes maintains the macrophage pool in the intestine of adult mice. Nat. Immunol. *15*, 929–937. https://doi.org/10.1038/ni.2967.

Bain, C.C., Gibson, D.A., Steers, N.J., Boufea, K., Louwe, P.A., Doherty, C., Gonzalez-Huici, V., Gentek, R., Magalhaes-Pinto, M., Shaw, T., et al. (2020). Rate of replenishment and microenvironment contribute to the sexually dimorphic phenotype and function of peritoneal macrophages. Sci. Immunol. *5*, eabc4466. https://doi.org/10.1126/sciimmunol.abc4466.

Bain, C.C., Hawley, C.A., Garner, H., Scott, C.L., Schridde, A., Steers, N.J., Mack, M., Joshi, A., Guilliams, M., Mowat, A.M.I., et al. (2016). Long-lived self-renewing bone marrow-derived macrophages displace embryo-derived cells to inhabit adult serous cavities. Nat. Commun. *7*, ncomms11852. https://doi.org/10.1038/ncomms11852.

Bedard, K., and Krause, K.H. (2007). The NOX family of ROS-generating NADPH oxidases: physiology and pathophysiology. Physiol. Rev. *87*, 245–313. https://doi.org/10.1152/physrev.00044.2005.

Bellomo, A., Mondor, I., Spinelli, L., Lagueyrie, M., Stewart, B.J., Brouilly, N., Malissen, B., Clatworthy, M.R., and Bajenoff, M. (2020). Reticular fibroblasts expressing the transcription factor WT1 define a stromal niche that maintains and replenishes splenic red pulp macrophages. Immunity *53*, 127–142.e7. https://doi.org/10.1016/j.immuni.2020.06.008.

Bielohuby, M., Herbach, N., Wanke, R., Maser-Gluth, C., Beuschlein, F., Wolf, E., and Hoeflich, A. (2007). Growth analysis of the mouse adrenal gland from weaning to adulthood: time- and gender-dependent alterations of cell size and number in the cortical compartment. Am. J. Physiol. Endocrinol. Metab. *293*, E139–E146. https://doi.org/10.1152/ajpendo.00705.2006.

Butler, A., Hoffman, P., Smibert, P., Papalexi, E., and Satija, R. (2018). Integrating single-cell transcriptomic data across different conditions, technologies, and species. Nat. Biotechnol. *36*, 411–420. https://doi.org/10.1038/nbt.4096.

Buttgereit, A., Lelios, I., Yu, X., Vrohlings, M., Krakoski, N.R., Gautier, E.L., Nishinakamura, R., Becher, B., and Greter, M. (2016). Sall1 is a transcriptional regulator defining microglia identity and function. Nat. Immunol. *17*, 1397–1406. https://doi.org/10.1038/ni.3585.

Calderon, B., Carrero, J.A., Ferris, S.T., Sojka, D.K., Moore, L., Epelman, S., Murphy, K.M., Yokoyama, W.M., Randolph, G.J., and Unanue, E.R. (2015). The pancreas anatomy conditions the origin and properties of resident macrophages. J. Exp. Med. *212*, 1497–1512. https://doi.org/10.1084/jem.20150496.

Carrero, J.A., McCarthy, D.P., Ferris, S.T., Wan, X., Hu, H., Zinselmeyer, B.H., Vomund, A.N., and Unanue, E.R. (2017). Resident macrophages of pancreatic islets have a seminal role in the initiation of autoimmune diabetes of NOD mice. Proc. Natl. Acad. Sci. U S A *114*, E10418–E10427. https://doi.org/10.1073/pnas.1713543114.

Cecchini, M.G., Dominguez, M.G., Mocci, S., Wetterwald, A., Felix, R., Fleisch, H., Chisholm, O., Hofstetter, W., Pollard, J.W., and Stanley, E.R. (1994). Role of colony stimulating factor-1 in the establishment and regulation of tissue macrophages during postnatal development of the mouse. Development *120*, 1357–1372. https://doi.org/10.1242/dev.120.6.1357.

Chakarov, S., Lim, H.Y., Tan, L., Lim, S.Y., See, P., Lum, J., Zhang, X.M., Foo, S., Nakamizo, S., Duan, K., et al. (2019). Two distinct interstitial macrophage

populations coexist across tissues in specific subtissular niches. Science *363*, eaau0964. https://doi.org/10.1126/science.aau0964.

Cox, N., Pokrovskii, M., Vicario, R., and Geissmann, F. (2021). Origins, biology, and diseases of tissue macrophages. Annu. Rev. Immunol. *39*, 313–344. https://doi.org/10.1146/annurev-immunol-093019-111748.

Croxford, A.L., Lanzinger, M., Hartmann, F.J., Schreiner, B., Mair, F., Pelczar, P., Clausen, B.E., Jung, S., Greter, M., and Becher, B. (2015). The cytokine GM-CSF drives the inflammatory signature of CCR2+ monocytes and licenses autoimmunity. Immunity *43*, 502–514. https://doi.org/10.1016/j.immuni.2015.08.010.

Dai, X.M., Ryan, G.R., Hapel, A.J., Dominguez, M.G., Russell, R.G., Kapp, S., Sylvestre, V., and Stanley, E.R. (2002). Targeted disruption of the mouse colony-stimulating factor 1 receptor gene results in osteopetrosis, mononuclear phagocyte deficiency, increased primitive progenitor cell frequencies, and reproductive defects. Blood *99*, 111–120. https://doi.org/10.1182/blood.v99.1.111.

Dick, S.A., Macklin, J.A., Nejat, S., Momen, A., Clemente-Casares, X., Althagafi, M.G., Chen, J., Kantores, C., Hosseinzadeh, S., Aronoff, L., et al. (2019). Self-renewing resident cardiac macrophages limit adverse remodeling following myocardial infarction. Nat. Immunol. *20*, 29–39. https://doi.org/10.1038/s41590-018-0272-2.

Dick, S.A., Wong, A., Hamidzada, H., Nejat, S., Nechanitzky, R., Vohra, S., Mueller, B., Zaman, R., Kantores, C., Aronoff, L., et al. (2022). Three tissue resident macrophage subsets coexist across organs with conserved origins and life cycles. Sci. Immunol. *7*, eabf7777. https://doi.org/10.1126/sciimmunol.abf7777.

Ensan, S., Li, A., Besla, R., Degousee, N., Cosme, J., Roufaiel, M., Shikatani, E.A., El-Maklizi, M., Williams, J.W., Robins, L., et al. (2016). Self-renewing resident arterial macrophages arise from embryonic CX3CR1(+) precursors and circulating monocytes immediately after birth. Nat. Immunol. *17*, 159–168. https://doi.org/10.1038/ni.3343.

Epelman, S., Lavine, K.J., and Randolph, G.J. (2014). Origin and functions of tissue macrophages. Immunity *41*, 21–35. https://doi.org/10.1016/j.immuni.2014.06.013.

Ferris, S.T., Zakharov, P.N., Wan, X., Calderon, B., Artyomov, M.N., Unanue, E.R., and Carrero, J.A. (2017). The islet-resident macrophage is in an inflammatory state and senses microbial products in blood. J. Exp. Med. *214*, 2369–2385. https://doi.org/10.1084/jem.20170074.

Gannon, A.L., O'Hara, L., Mason, J.I., Jorgensen, A., Frederiksen, H., Milne, L., Smith, S., Mitchell, R.T., and Smith, L.B. (2019). Androgen receptor signalling in the male adrenal facilitates X-zone regression, cell turnover and protects against adrenal degeneration during ageing. Sci. Rep. *9*, 10457. https://doi.org/10.1038/s41598-019-46049-3.

Gautier, E.L., Shay, T., Miller, J., Greter, M., Jakubzick, C., Ivanov, S., Helft, J., Chow, A., Elpek, K.G., Gordonov, S., et al. (2012). Gene-expression profiles and transcriptional regulatory pathways that underlie the identity and diversity of mouse tissue macrophages. Nat. Immunol. *13*, 1118–1128. https://doi.org/10.1038/ni.2419.

Ginhoux, F., and Guilliams, M. (2016). Tissue-resident macrophage ontogeny and homeostasis. Immunity *44*, 439–449. https://doi.org/10.1016/j.immuni.2016.02.024.

Ginhoux, F., Greter, M., Leboeuf, M., Nandi, S., See, P., Gokhan, S., Mehler, M.F., Conway, S.J., Ng, L.G., Stanley, E.R., et al. (2010). Fate mapping analysis reveals that adult microglia derive from primitive macrophages. Science *330*, 841–845. https://doi.org/10.1126/science.1194637.

Ginhoux, F., Liu, K., Helft, J., Bogunovic, M., Greter, M., Hashimoto, D., Price, J., Yin, N., Bromberg, J., Lira, S.A., et al. (2009). The origin and development of nonlymphoid tissue CD103+ DCs. J. Exp. Med. *206*, 3115–3130. https://doi.org/10.1084/jem.20091756.

Perdiguero, E.G., Klapproth, K., Schulz, C., Busch, K., Azzoni, E., Crozet, L., Garner, H., Trouillet, C., de Bruijn, M.F., Geissmann, F., and Rodewald, H.R. (2015). Tissue-resident macrophages originate from yolk-sac-derived erythro-myeloid progenitors. Nature *43*, S64–S551. https://doi.org/10.1016/j.exphem.2015.06.130.

Guilliams, M., De Kleer, I., Henri, S., Post, S., Vanhoutte, L., De Prijck, S., Deswarte, K., Malissen, B., Hammad, H., and Lambrecht, B.N. (2013). Alveolar macrophages develop from fetal monocytes that differentiate into long-lived cells in the first week of life via GM-CSF. J. Exp. Med. *210*, 1977–1992. https://doi.org/10.1084/jem.20131199.

Hafemeister, C., and Satija, R. (2019). Normalization and variance stabilization of single-cell RNA-seq data using regularized negative binomial regression. Genome Biol. *20*, 296. https://doi.org/10.1186/s13059-019-1874-1.

Huang, C.C.J., and Kang, Y. (2019). The transient cortical zone in the adrenal gland: the mystery of the adrenal X-zone. J. Endocrinol. *241*, R51–R63. https://doi.org/10.1530/joe-18-0632.

Hume, D.A., Halpin, D., Charlton, H., and Gordon, S. (1984). The mononuclear phagocyte system of the mouse defined by immunohistochemical localization of antigen F4/80: macrophages of endocrine organs. Proc. Natl. Acad. Sci. U S A *81*, 4174–4177. https://doi.org/10.1073/pnas.81.13.4174.

Ivanov, S., Gallerand, A., Gros, M., Stunault, M.I., Merlin, J., Vaillant, N., Yvan-Charvet, L., and Guinamard, R.R. (2019). Mesothelial cell CSF1 sustains peritoneal macrophage proliferation. Eur. J. Immunol. *49*, 2012–2018. https://doi.org/10.1002/eji.201948164.

Jakubzick, C., Gautier, E.L., Gibbings, S.L., Sojka, D.K., Schlitzer, A., Johnson, T.E., Ivanov, S., Duan, Q., Bala, S., Condon, T., et al. (2013). Minimal differentiation of classical monocytes as they survey steady-state tissues and transport antigen to lymph nodes. Immunity *39*, 599–610. https://doi.org/10.1016/j.immuni.2013.08.007.

Jokela, H., Lokka, E., Kiviranta, M., Tyystjarvi, S., Gerke, H., Elima, K., Salmi, M., and Rantakari, P. (2020). Fetal-derived macrophages persist and sequentially maturate in ovaries after birth in mice. Eur. J. Immunol. *50*, 1500–1514. https://doi.org/10.1002/eji.202048531.

Kim, D., Paggi, J.M., Park, C., Bennett, C., and Salzberg, S.L. (2019). Graph-based genome alignment and genotyping with HISAT2 and HISAT-genotype. Nat. Biotechnol. *37*, 907–915. https://doi.org/10.1038/s41587-019-0201-4.

Kohyama, M., Ise, W., Edelson, B.T., Wilker, P.R., Hildner, K., Mejia, C., Frazier, W.A., Murphy, T.L., and Murphy, K.M. (2009). Role for Spi-C in the development of red pulp macrophages and splenic iron homeostasis. Nature *457*, 318–321. https://doi.org/10.1038/nature07472.

Koppelman, B., Neefjes, J.J., de Vries, J.E., and de Waal Malefyt, R. (1997). Interleukin-10 down-regulates MHC class II $\alpha\beta$ peptide complexes at the plasma membrane of monocytes by affecting arrival and recycling. Immunity *7*, 861–871. https://doi.org/10.1016/s1074-7613(00)80404-5.

Lacroix, A., Feelders, R.A., Stratakis, C.A., and Nieman, L.K. (2015). Cushing's syndrome. Lancet *386*, 913–927. https://doi.org/10.1016/s0140-6736(14)61375-1.

Liao, Y., Smyth, G.K., and Shi, W. (2014). featureCounts: an efficient general purpose program for assigning sequence reads to genomic features. Bioinformatics *30*, 923–930. https://doi.org/10.1093/bioinformatics/btt656.

Lim, H.Y., Lim, S.Y., Tan, C.K., Thiam, C.H., Goh, C.C., Carbajo, D., Chew, S.H.S., See, P., Chakarov, S., Wang, X.N., et al. (2018). Hyaluronan receptor LYVE-1-expressing macrophages maintain arterial tone through hyaluronan-mediated regulation of smooth muscle cell collagen. Immunity *49*, 326–341.e7. https://doi.org/10.1016/j.immuni.2018.06.008.

Liu, Z., Gu, Y., Chakarov, S., Bleriot, C., Kwok, I., Chen, X., Shin, A., Huang, W., Dress, R.J., Dutertre, C.A., et al. (2019). Fate mapping via ms4a3-expression history traces monocyte-derived cells. Cell *178*, 1509–1525.e19. https://doi.org/10.1016/j.cell.2019.08.009.

Lokka, E., Lintukorpi, L., Cisneros-Montalvo, S., Makela, J.A., Tyystjarvi, S., Ojasalo, V., Gerke, H., Toppari, J., Rantakari, P., and Salmi, M. (2020). Generation, localization and functions of macrophages during the development of testis. Nat. Commun. *11*, 4375. https://doi.org/10.1038/s41467-020-18206-0.

Love, M.I., Huber, W., and Anders, S. (2014). Moderated estimation of fold change and dispersion for RNA-seq data with DESeq2. Genome Biol. *15*, 550. https://doi.org/10.1186/s13059-014-0550-8.

Lyraki, R., and Schedl, A. (2021). Adrenal cortex renewal in health and disease. Nat. Rev. Endocrinol. *17*, 421–434. https://doi.org/10.1038/s41574-021-00491-4.

McGinnis, C.S., Murrow, L.M., and Gartner, Z.J. (2019). DoubletFinder: doublet detection in single-cell RNA sequencing data using artificial nearest neighbors. Cell Syst. *8*, 329–337.e4. https://doi.org/10.1016/j.cels.2019.03.003.

Molawi, K., Wolf, Y., Kandalla, P.K., Favret, J., Hagemeyer, N., Frenzel, K., Pinto, A.R., Klapproth, K., Henri, S., Malissen, B., et al. (2014). Progressive replacement of embryo-derived cardiac macrophages with age. J. Exp. Med. *211*, 2151–2158. https://doi.org/10.1084/jem.20140639.

Mossadegh-Keller, N., Gentek, R., Gimenez, G., Bigot, S., Mailfert, S., and Sieweke, M.H. (2017). Developmental origin and maintenance of distinct testicular macrophage populations. J. Exp. Med. *214*, 2829–2841. https://doi.org/10.1084/jem.20170829.

Nishinakamura, R., Wiler, R., Dirksen, U., Morikawa, Y., Arai, K., Miyajima, A., Burdach, S., and Murray, R. (1996). The pulmonary alveolar proteinosis in granulocyte macrophage colony-stimulating factor/interleukins 3/5 beta c receptor-deficient mice is reversed by bone marrow transplantation. J. Exp. Med. *183*, 2657–2662. https://doi.org/10.1084/jem.183.6.2657.

Pridans, C., Raper, A., Davis, G.M., Alves, J., Sauter, K.A., Lefevre, L., Regan, T., Meek, S., Sutherland, L., Thomson, A.J., et al. (2018). Pleiotropic impacts of macrophage and microglial deficiency on development in rats with targeted mutation of the Csf1r locus. J. Immunol. *201*, 2683–2699. https://doi.org/10.4049/jimmunol.1701783.

Rajamohan, S.B., Raghuraman, G., Prabhakar, N.R., and Kumar, G.K. (2012). NADPH oxidase-derived H(2)O(2) contributes to angiotensin II-induced aldosterone synthesis in human and rat adrenal cortical cells. Antioxidants Redox Signal. *17*, 445–459. https://doi.org/10.1089/ars.2011.4176.

Ryan, G.R., Dai, X.M., Dominguez, M.G., Tong, W., Chuan, F., Chisholm, O., Russell, R.G., Pollard, J.W., and Stanley, E.R. (2001). Rescue of the colony-stimulating factor 1 (CSF-1)-nullizygous mouse (Csf1(op)/Csf1(op)) phenotype with a CSF-1 transgene and identification of sites of local CSF-1 synthesis. Blood *98*, 74–84. https://doi.org/10.1182/blood.v98.1.74.

Saelens, W., Cannoodt, R., Todorov, H., and Saeys, Y. (2019). A comparison of single-cell trajectory inference methods. Nat. Biotechnol. *37*, 547–554. https://doi.org/10.1038/s41587-019-0071-9.

Sakai, M., Troutman, T.D., Seidman, J.S., Ouyang, Z., Spann, N.J., Abe, Y., Ego, K.M., Bruni, C.M., Deng, Z., Schlachetzki, J.C.M., et al. (2019). Liver-derived signals sequentially reprogram myeloid enhancers to initiate and maintain kupffer cell identity. Immunity *51*, 655–670.e8. https://doi.org/10.1016/j.immuni.2019.09.002.

Satpathy, A.T., Briseno, C.G., Lee, J.S., Ng, D., Manieri, N.A., Kc, W., Wu, X., Thomas, S.R., Lee, W.L., Turkoz, M., et al. (2013). Notch2-dependent classical dendritic cells orchestrate intestinal immunity to attaching-and-effacing bacterial pathogens. Nat. Immunol. *14*, 937–948. https://doi.org/10.1038/ni.2679.

Schulz, C., Gomez Perdiguero, E., Chorro, L., Szabo-Rogers, H., Cagnard, N., Kierdorf, K., Prinz, M., Wu, B., Jacobsen, S.E.W., Pollard, J.W., et al. (2012). A lineage of myeloid cells independent of Myb and hematopoietic stem cells. Science *336*, 86–90. https://doi.org/10.1126/science.1219179.

Scott, C.L., Zheng, F., De Baetselier, P., Martens, L., Saeys, Y., De Prijck, S., Lippens, S., Abels, C., Schoonooghe, S., Raes, G., et al. (2016). Bone marrow-derived monocytes give rise to self-renewing and fully differentiated Kupffer cells. Nat. Commun. *7*, 10321. https://doi.org/10.1038/ncomms10321.

Serbina, N.V., and Pamer, E.G. (2006). Monocyte emigration from bone marrow during bacterial infection requires signals mediated by chemokine receptor CCR2. Nat. Immunol. *7*, 311–317. https://doi.org/10.1038/ni1309.

Sergushichev, A.A., Loboda, A.A., Jha, A.K., Vincent, E.E., Driggers, E.M., Jones, R.G., Pearce, E.J., and Artyomov, M.N. (2016). GAM: a web-service for integrated transcriptional and metabolic network analysis. Nucleic Acids Res. *44*, W194–W200. https://doi.org/10.1093/nar/gkw266.

Silva, H.M., Bafica, A., Rodrigues-Luiz, G.F., Chi, J., Santos, P.D.A., Reis, B.S., Hoytema van Konijnenburg, D.P., Crane, A., Arifa, R.D.N., Martin, P., et al. (2019). Vasculature-associated fat macrophages readily adapt to inflammatory and metabolic challenges. J. Exp. Med. *216*, 786–806. https://doi.org/10.1084/jem.20181049.

Street, K., Risso, D., Fletcher, R.B., Das, D., Ngai, J., Yosef, N., Purdom, E., and Dudoit, S. (2018). Slingshot: cell lineage and pseudotime inference for single-cell transcriptomics. BMC Genom. *19*, 477. https://doi.org/10.1186/s12864-018-4772-0.

Stuart, T., Butler, A., Hoffman, P., Hafemeister, C., Papalexi, E., Mauck, W.M., 3rd, Hao, Y., Stoeckius, M., Smibert, P., and Satija, R. (2019). Comprehensive integration of single-cell data. Cell *177*, 1888–1902.e21. https://doi.org/10.1016/j.cell.2019.05.031.

Unanue, E.R., Turk, V., and Neefjes, J. (2016). Variations in MHC class II antigen processing and presentation in health and disease. Annu. Rev. Immunol. *34*, 265–297. https://doi.org/10.1146/annurev-immunol-041015-055420.

Williams, J.W., Elvington, A., Ivanov, S., Kessler, S., Luehmann, H., Baba, O., Saunders, B.T., Kim, K.W., Johnson, M.W., Craft, C.S., et al. (2017). Thermoneutrality but not UCP1 deficiency suppresses monocyte mobilization into blood. Circ. Res. *121*, 662–676. https://doi.org/10.1161/circresaha.117.311519.

Wolf, Y., Boura-Halfon, S., Cortese, N., Haimon, Z., Sar Shalom, H., Kuperman, Y., Kalchenko, V., Brandis, A., David, E., Segal-Hayoun, Y., et al. (2017). Brown-adipose-tissue macrophages control tissue innervation and homeostatic energy expenditure. Nat. Immunol. *18*, 665–674. https://doi.org/10.1038/ni.3746.

Yona, S., Kim, K.W., Wolf, Y., Mildner, A., Varol, D., Breker, M., Strauss-Ayali, D., Viukov, S., Guilliams, M., Misharin, A., et al. (2013). Fate mapping reveals origins and dynamics of monocytes and tissue macrophages under homeostasis. Immunity *38*, 79–91. https://doi.org/10.1016/j.immuni.2012.12.001.

Zennaro, M.C., Boulkroun, S., and Fernandes-Rosa, F.L. (2020). Pathogenesis and treatment of primary aldosteronism. Nat. Rev. Endocrinol. *16*, 578–589. https://doi.org/10.1038/s41574-020-0382-4.

STAR★METHODS

KEY RESOURCES TABLE

REAGENT or RESOURCE	SOURCE	IDENTIFIER
Antibodies		
CD11b PE-Cy5 (Clone M1/70)	Biolegend	Cat# 101209, RRID:AB_312792
CD11b Brilliant Violet 510 (Clone M1/70)	Biolegend	Cat# 101263, RRID:AB_2629529
CD11b APC-Cy7 (Clone M1/70)	Biolegend	Cat# 101226, RRID:AB_830642
F4/80 PE-Cy7 (Clone BM8)	Biolegend	Cat# 123114, RRID:AB_893478
F4/80 Alexa Fluor 488 (Clone BM8)	Biolegend	Cat# 123120, RRID:AB_893479
F4/80 Brilliant Violet 650 (Clone BM8)	Biolegend	Cat# 123149, RRID:AB_2564589
F4/80 APC (Clone REA126)	Miltenyi	Cat# 130-116-525 RRID:AB_2733417
CD45 APC-Cy7 (Clone 30-F11)	BD Biosciences	Cat# 557659, RRID:AB_396774
CD45 Pacific Blue (Clone 30-F11)	Biolegend	Cat# 103126, RRID:AB_493535
CD45 VioGreen (Clone REA737)	Miltenyi	Cat# 130-110-803, RRID: AB_2658224
CD45 Brilliant Violet 570 (Clone 30-F11)	Biolegend	Cat# 103136, RRID:AB_10898325
CD45 APC-Vio 770 (Clone REA737)	Miltenyi	Cat# 130-110-662, RRID:AB_2658231
CD64 Brillant Violet 421 (Clone ×54-5/7.1)	Biolegend	Cat# 139309, RRID: AB_2562694
CD64 Brillant Violet 711 (Clone ×54-5/7.1)	Biolegend	Cat# 139311, RRID: AB_2563846
CD64 PE/Dazzle 594 (Clone ×54-5/7.1)	Biolegend	Cat# 139320, RRID:AB_2566559
Ly6C APC (Clone HK1.4)	Biolegend	Cat# 128015, RRID:AB_1732087
Ly6C PerCP-Cy5.5 (Clone HK1.4)	Biolegend	Cat# 128012, RRID: AB_1659241
Ly6G PerCP-Cy5.5 (Clone 1A8)	Biolegend	Cat# 127615, RRID:AB_1877272
Ly6G Brilliant Violet 510 (Clone 1A8)	Biolegend	Cat#127633, RRID: AB_2562937
Ly6G Brilliant Violet 785 (Clone 1A8)	Biolegend	Cat# 127645, RRID:AB_2566317
Klrg1 PE-Cy7 (Clone 2F1/KLRG1)	Biolegend	Cat# 138416, RRID:AB_2561736
CD115 PE-Cy7 (AFS98)	Biolegend	Cat# 135524, RRID: AB_2566460
CD115 PE (Clone AFS98)	Biolegend	Cat# 135506, RRID:AB_1937253
CD115 PE (Clone REA827)	Miltenyi	Cat# 130-112-639, RRID:AB_2654553
Gr-1 PerCP-Cy5.5 (Clone RB6-8C5)	Biolegend	Cat# 108426, RRID:AB_893557
NK1.1 FITC (Clone PK136)	Biolegend	Cat# 108706, RRID:AB_313393
Sca1 Pacific Blue (Clone D7)	Biolegend	Cat# 108120, RRID:AB_493273
CD19 PE (Clone REA749)	Miltenyi	Cat# 130-112-035, RRID:AB_2655822
CD19 BUV737 (Clone 1D3)	BD Biosciences	Cat# 612782, RRID:AB_2870111
CD3 APC (Clone 17A2)	Biolegend	Cat# 100236, RRID:AB_2561456
CD8a Brilliant Violet 510 (Clone 53-6.7)	Biolegend	Cat# 100752, RRID:AB_2563057
CD4 Alexa Fluor 700 (Clone RM4-5)	Biolegend	Cat# 100536, RRID:AB_493701
MHC-II IA/IE PE (Clone 2G9)	BD Biosciences	Cat# 558593, RRID:AB_647221
MHC-II IA/IE FITC (Clone 2G9)	BD Biosciences	Cat# 553623, RRID:AB_394958
MHC-II IA/IE Brilliant Violet 510 (Clone M5/ 114.15.2)	Biolegend	Cat# 107636, RRID : AB_2734168
MHC-II IA/IE Alexa Fluor 647 (Clone M5/ 114.15.2)	Biolegend	Cat# 107618, RRID:AB_493525
MHC-II VioBlue (Clone REA813)	Miltenyi	Cat# 130-112-394, RRID:AB_2652908
Timd4 PE-Cy7 (Clone RMT4-54)	Biolegend	Cat# 130010, RRID:AB_2565719
Timd4 PerCP-eFluor710 (Clone RMT4-54)	Invitrogen	Cat# 46-5866-80, RRID:AB_2573780
Lyve1 eFluor660 (Clone ALY7)	Invitrogen	Cat# 50-0443-80, RRID:AB_10598060
CD206 PerCP-Cy5.5 (Clone C068C2)	Biolegend	Cat# 141716, RRID:AB_2561992

(*Continued on next page*)

Continued

REAGENT or RESOURCE	SOURCE	IDENTIFIER
MerTK PE (Clone 2B10C42)	Biolegend	Cat# 151506, RRID:AB_2617037
MerTK APC (Clone 2B10C42)	Biolegend	Cat# 151508, RRID: AB_2650739
CD11c PE-Cy7 (Clone HL3)	BD Biosciences	Cat# 558079, RRID:AB_647251
CD24 BUV496 (Clone M1/69)	BD Biosciences	Cat# 612953, RRID:AB_2870229
NKp46 Brilliant Violet 421 (Clone 29A1.4)	Biolegend	Cat# 137611, RRID:AB_10915472
CCR2 APC-Fire750 (Clone SA203T11)	Biolegend	Cat# 150629, RRID :AB_2810416
CD40 BUV395 (Clone 3/23)	BD Biosciences	Cat# 745697, RRID: AB_2743179
CD40 APC-Vio770 (Clone REA965)	Miltenyi	Cat# 130-116-113, RRID:AB_2727355
CD80 BUV615 (Clone 16-10A1)	BD Biosciences	Cat# 751328, RRID:AB_2875337
CD80 PerCP-Vio770 (Clone REA983)	Miltenyi	Cat# 130-116-464, RRID:AB_2727561
CD86 BUV805 (Clone GL1)	BD Biosciences	Cat# 741946, RRID:AB_2871258
CD86 PE (Clone REA1190)	Miltenyi	Cat# 130-122-129, RRID:AB_2819412
ICOSL (CD275) PE-Vio770 (Clone REA990)	Miltenyi	Cat# 130-116-448, RRID:AB_2727549
CD68 Alexa Fluor 647 (Clone FA-11)	Biolegend	Cat# 137004, RRID:AB_2044002
*InVivo*MAb anti-mouse CSF1R (Clone AFS98)	BioXCell	Cat# BE0213, RRID:AB_2687699
*InVivo*MAb rat IgG2a isotype control, anti-trinitrophenol (Clone 2A3)	BioXCell	Cat# BE0089, RRID:AB_1107769
InVivoMAb anti-mouse IL10R (Clone BE0050)	BioXCell	Cat# BE0050, RRID:AB_1107611
InVivoMAb anti-mouse CD16/CD32 (Clone 2.4G2)	BioXCell	Cat# BE0307, RRID:AB_2736987
Chemicals, peptides, and recombinant Proteins		
DAPI	Sigma	Cat# D9542
LIVE/DEAD^{TM} Fixable Violet Dead Cell Stain Kit	ThermoFisher	Cat# L34955
PFA 4%	VWR International	Cat# 9713.1000
Bovine serum Albumin (BSA)	Sigma	Cat# A7030
Tamoxifen	Sigma	Cat# T5648
Collagenase A	Sigma	Cat# 11088793001
IHC Antigen retrieval solution	eBiosciences	Cat# 00-4955-58
Antifade mounting medium with DAPI	Vectashield	Cat# H-1500
Fetal bovine serum	Fisher Scientific	Cat# 12350273
Lysing buffer	BD Biosciences	Cat# 555899
Liberase	Roche	Cat# 05401054001
DNAse I	Roche	Cat# 10104159001
Penicillin Streptomycin	Life Technologies	Cat# 15070063
L-Glutamine	Life Technologies	Cat# 25030024
Mouse M-CSF	Miltenyi Biotec	Cat# 130-094-129
RPMI medium	Life Technologies	Cat# 21875091
Clorgyline	Abcam	Cat# ab145646
Norepinephrine	Sigma	Cat# A7257
TRITC-Dextran 65–85 kDa	Sigma	Cat# T1162
Bodipy	Thermofisher	Cat# D3922
Critical commercial assays		
Mouse CCL2 DuoSet ELISA	R&D Systems	Cat# DY479-05
Norepinephrine ELISA Kit	Tebu-bio	Cat# 157KA1891
Aldosterone Parameter Assay Kit	R&D Systems	Cat# KGE016
Corticosterone Parameter Assay Kit	R&D Systems	Cat# KGE009

(*Continued on next page*)

Continued		
REAGENT or RESOURCE	SOURCE	IDENTIFIER
Ki67 Staining Kit PE	BD Biosciences	Cat# 51-36525×
FoxP3 Staining Buffer Set	Miltenyi	Cat# 130-093-142
RNA extraction kit	QIAGEN	Cat# 74136
Software and algorithms		
Prism 8	GraphPad	N/A
Chromeleon software	Thermo Scientific	N/A
FlowJo	Tree Star	N/A
Seurat package version 3.1.0	https://satijalab.org/seurat/	N/A
BD FACSDiva	BD Biosciences	N/A
ImageJ	NIH	N/A
SpectroFlo	Cytek	N/A
Phantasus	Artyomov Lab	N/A

RESOURCE AVAILABILITY

Lead contact

Further information and requests for resources and reagents should be directed to and will be fulfilled by the lead contact Dr. Stoyan Ivanov (Stoyan.ivanov@unice.fr).

Materials availability

This study did not generate new unique reagents.

Data and code availability

All sequencing datasets in this article are deposited in an international public repository, Gene Expression Omnibus (GEO), under accession ID GSE203096 for bulk RNA sequencing and GSE203095 for single cell RNA sequencing data. This paper does not report original code. Any additional information required to reanalyze the data reported in this paper is available from the lead contact upon request.

EXPERIMENTAL MODEL AND SUBJECT DETAILS

Wild-type C57BL/6J mice were purchased from Janvier Labs. CX3CR1^{gfp} (B6.Cg-Ptprca Cx3cr1$^{tm1Litt/LittJ}$), Lyz2^{cre} (B6.129P2-*Lyz2*$^{tm1(cre)Ifo}$/J), CD115^{creERT2} (FVB-Tg(Csf1r-cre/Esr1*)1Jwp/J), R26^{TdTomato} (B6.Cg-Gt(ROSA)26Sor$^{tm9(CAG-tdTomato)Hze}$/J) and CX3CR1^{creERT2} (B6.129P2(C)-*Cx3cr1*$^{tm2.1(cre/ERT2)Jung}$/J) were on B6 background. CCR2$^{cre/ERT2}$ (C57BL/6NTac-Ccr2$^{tm2982(T2A-Cre7ESR1-T2A-mKate2)}$) (Croxford et al., 2015) mice were kindly provided by Dr. Burkhard Becher and crossed with R26^{TdTomato} and CCR2^{GFP} mice (B6(C)-Ccr2$^{tm1.1Cln}$/J) provided by Dr. Marco Colonna (Satpathy et al., 2013). Ms4a3^{cre} (C57BL/6J-Ms4a3$^{em2(cre)Fgnx/J}$) mice, initially described in (Liu et al., 2019), were kindly provided by Dr. Florent Ginhoux. When possible, co-housed littermate controls were used. Because age appeared as a relevant parameter, mice of different ages were used as indicated in each figure legend. Age-matched animals were used for male and female comparisons. All mice were bred and housed in specific pathogen-free conditions maintained by animal facilities in either the Mediterranean Center of Molecular Medicine (INSERM U1065, Université Côte d'Azur), the University of Minnesota Medical School Research Animal Resources, the University of Illinois at Chicago, or the EOPS2 facility, Lille University Hospital Campus. Facilities were maintained at ambient temperature of ~20–23°C, with 12/12-h light/dark cycle and food available ad libitum. Animal protocols were authorized by the French Ministry of Higher Education and Research upon approval of the local ethical committees, by the Institutional Animal Care and Use Committee (IACUC) at University of Minnesota Medical School, by the IACUC at University of Illinois College of Medicine.

METHOD DETAILS

Tamoxifen treatments

CD115^{creERT2} x R26^{TdTomato} reporter mice were treated with tamoxifen dissolved in corn oil (20 mg/mL) by oral gavage (200 μL/mouse) on three consecutive days and were sacrificed 24 h after the last dose was administered.

CCR2^{creERT2} x R26^{TdTomato} reporter mice were treated with tamoxifen dissolved in corn oil (20 mg/mL) by a single oral gavage (250 μL/mouse). Animals were sacrificed 2, 7, or 14 days later and assessed for labeling efficiency in blood and adrenal glands by flow cytometry.

Pregnant CX3CR1^{creERT2} x R26^{TdTomato} mice were given 4 mg of TAM dissolved in corn oil. Mice were treated by oral gavage at the indicated embryonic day. Pups were obtained by cesarean delivery at E19.5 and taken care of by foster mothers until weaning periods. Brain microglia labeling was >97% and blood monocytes labeling was below 0.3% in all mice that received embryonic labeling. For pulse-chase experiments in adults, CX3CR1^{creERT2} x R26^{TdTomato} mice were injected intra-peritoneally with 10 mg/mL TAM (200 μL per mouse in 10% EtOH and sunflower oil) daily for 5 days. Labelling efficiency was assessed in brain, blood and adrenal glands for each experiment involving this strain.

Surgery procedures

Castration or control procedure (Sham) were performed on 3-week-old C57BL/6 male mice, which were sacrificed 4 weeks later for analysis of adrenal glands. Ovariectomy (OVX) and sham surgery were performed on 6-week-old C57BL/6 female mice, in accordance with the Institutional Ethics Committee on Laboratory Animals (CIEPAL-Azur, Nice Sophia-Antipolis, France). Mice were sacrificed 6 weeks after surgery.

In vivo macrophage depletion

C57BL/6 mice received an intraperitoneal injection of 500 μg *InVivo*MAb anti-mouse CSF1R (CD115) antibody (Clone AFS98, BioXCell cat No. BE0213) or isotype control (Clone 2A3, BioXCell cat No. BE0089). The injection was repeated 2 days later. Mice were sacrificed 16 h after the second injection. For cold exposure, macrophage depleted, and control mice were housed for 12 h at 4°C. Mice were single housed with identical light/dark cycle and food available ad libitum.

In vivo IL-10R blockade

5- to 12-week-old C57BL/6 mice received an intraperitoneal injection of 250 μg *InVivo*MAb anti-mouse IL-10R antibody (clone 1B1.3A, BioXCell cat no. BE0050) or PBS. The injection was repeated 2 days later. Mice were sacrificed 16 h after the second injection.

Flow cytometry analysis

Tissues were harvested and washed in PBS. Adipose depots surrounding the adrenal glands were carefully removed before shredding the tissue with scissors. Then the samples were incubated for 30 min at 37°C in PBS containing 300 μg/mL Liberase and 100 μg/mL DNAse I. The resulting suspension was homogenized using a syringe and a 20G needle, passed through a 100 μm sieve and centrifuged at 400g for 5 min. Cells were then resuspended in FACS buffer (PBS containing 0.3 mM EDTA and 0.06% BSA), stained for 30 min at 4°C in the dark and then washed. A viability stain (DAPI or Live/Dead fixable viability dye) was used whenever possible. Conventional cytometry data were acquired on a BD FACS Canto II or BD LSRFortessa X-20. Spectral cytometry data were acquired on a Cytek Aurora cytometer. All analysis, including unsupervised t-SNE analysis, was performed using FlowJo software (Tree Star).

SCENITH

The method was performed as described in (Arguello et al., 2020). SCENITH reagents kit (inhibitors, puromycin and antibodies) were obtained from www.scenith.com/try-it and used according to the provided protocol for ex-vivo analysis of myeloid cells. Briefly, adrenal glands were harvested, added to 50 μL DMEM (4.5g/L glucose) containing either Control, 2-Deoxy-D-Glucose (100mM), Oligomycin (1μM), 2-Deoxy-D-Glucose + Oligomycin or Harringtonine (2μg/mL), minced and incubated at 37°C for 30 min. Puromycin (10μg/mL), Liberase (300μg/mL) and DNAse I (100μg/mL) were then added to each tube. Adrenal glands were further incubated at 37°C for 30 min, and were then washed with 1mL cold PBS and put on ice. The suspension was homogenized using a syringe and a 20G needle, passed through a 100 μm sieve and centrifuged at 400g for 5 min. Cells were first stained with Live/Dead fixable viability dye, washed, and incubated with Fc Block (2.4G2, BioXcell) before surface staining. Cells were fixed and permeabilized using Foxp3 fixation/permeabilization buffer (Miltenyi) and then stained with anti-Puromycin (Alexa Fluor 647). Cells that were not incubated with Puromycin were used as a negative control for anti-Puromycin signal. Cells that received surface staining but no anti-Puromycin staining (full minus one) were used to measure subset-specific autofluorescence in the AF647 channel, and this background signal was subtracted. Glucose dependence, Mitochondrial dependence and Glycolytic capacity were calculated as previously described (Arguello et al., 2020).

Dextran uptake assay

Mice were injected intravenously with 100 μL TRITC-Dextran solution (65–85 kDa, 10 mg/mL diluted in PBS) and sacrificed twenty minutes later. Adrenal glands and epididymal adipose tissue were then processed and analyzed by flow cytometry. PBS-injected mice were used as controls to determine autofluorescence.

Norepinephrine degradation assay

Adrenal glands were processed as previously described. CD45$^+$CD64$^+$MerTK$^+$ cells were sorted using a BD FACSAria II Cell Sorter. Macrophages were cultured overnight in RPMI medium (10% SVF, 2 mM L-Glutamine, 50 U/mL Penicillin, 50 μg/mL Streptomycin) containing 50 ng/mL recombinant M-CSF, as well as norepinephrine (NE) (5 μM) and/or clorgyline (100 μM). Supernatants were collected at the end of the incubation period and macrophage viability was assessed using Live/Dead staining.

ELISA assays

Blood was collected by submandibular bleeding. One adrenal gland was crushed in 200μL PBS using 0.1 mm glass beads and a precellys homogenizer (Biospec cat No. 11079101). Corticosterone and aldosterone levels were measured in serum and adrenal gland homogenates as instructed (R&D Systems catalog No.KGE009 and No.KGE016 respectively). Norepinephrine was measured in AGM culture supernatant using Norepinephrine ELISA Kit (Abnova cat No.KA1891).

Bulk RNA-seq

RNA was extracted from adrenal glands using RNA extraction kit (QIAGEN cat No. 7413). Both adrenals were crushed using 0.1 mm glass beads in a precellys homogenizer (Biospec cat No. 11079101) containing RLT buffer from the RNA extraction kit. Total RNAs were extracted in RNase-free water from adrenals and were analyzed in DNBseq™ sequencing plateform using DNBSEQ stranded mRNA library. Paired RNA-seq reads were aligned to the Ensembl 84 Mus musculus reference genome with hisat2 (version 2.2.1) (Kim et al., 2019). Count matrixes for each sample were obtained by featureCounts (version 2.0.1) in reversely stranded mode (Liao et al., 2014). Differential expression analysis was implemented by DESeq2 (version 1.30.1) (Love et al., 2014). Principal component analysis (PCA) was performed based on variance stabilized transformation output, and the heatmaps were drawn by Phantasus based on raw counts under the regularized log transformation (https://genome.ifmo.ru/phantasus, https://artyomovlab.wustl.edu/phantasus). Analysis of metabolic pathways was performed with Shiny GAM (Sergushichev et al., 2016).

Quantification of serum cation concentration

K^+ concentrations in serum was evaluated by ion chromatography analysis. Serum samples and ion standard solutions were previously diluted (1/100), deproteinized by addition of acetonitrile (dilution 1:1 volume) mixed and centrifuged at 12,000g (10 min at 4°C). Ion concentrations of the supernatants were determined using an ion chromatography Dionex ICS-5000 plus system (Thermo Scientific). The system included an autosampler, pumps, eluent generator and conductivity detectors. The system is equipped with an eluent generator cartridge (Dionex EGC500MSA) and a cation column (IonPac AS-11 HC, 2 mm). Ion concentrations were determined using Chromeleon software (Thermo Scientific) by measuring surface area of the peaks and were compared to the corresponding ion standard profiles.

Single-cell RNA-seq data analysis

Cells were loaded on a Chromium Controller (10× Genomics) with a target output of 5000 cells per sample. Reverse transcription, cDNA synthesis/amplification and library preparation were performed according to the 10× Genomics protocol (Chromium™ Single Cell 3′ Reagent Kit, v3.1 Chemistry). scRNA libraries were sequenced on an Illumina NextSeq 500/550 High Output flowcell: the forward read had a length of 28 bases that included the cell barcode and the UMI; the reverse read had a length of 55 bases that contained the cDNA insert.

Alignment, barcode assignment and UMI counting with Cell Ranger v4.0.0 was used to perform sample demultiplexing, barcode processing and single-cell 3' counting. Cell Ranger's mkfastq function was used to demultiplex raw base call files from the HiSeq4000 sequencer into sample specific FASTQ files.

Barcodes in both samples that were considered to represent noise and low-quality cells were filtered out using knee-inflection approach available in DropletUtils package (version 1.4.3). For analysis, Seurat package (version 3.1.0) was used, genes which express in less than 2 cells and cells which have non-zero counts in less than 200 genes were additionally filtered from both barcode expression matrices, and the result matrices were used as analysis inputs (Butler et al., 2018). The fraction of mitochondrial genes was calculated for every cell, and cells with a mitochondrial fraction >1.2% were filtered out. After all filtering procedures, 3,636 cells were left in the scRNA-seq data of the female sample, and 2,240 cells were left in scRNA-seq of the male sample.

Both samples were normalized using SCTransform function with mitochondrial content as variable to regress out in a second non-regularized linear regression (Hafemeister and Satija, 2019). For integration purpose, variable features across the samples were selected by SelectIntegrationFeatures function with the number of features equal to 2000. Then the object was prepared for integration (PrepSCTIntegration function), the anchors were found (FindIntegrationAnchors function) and the samples were merged into the whole object (IntegrateData function) (Stuart et al., 2019). The dimensionality of the object was reduced by principal component analysis (PCA), and the first 20 principal components (PCs) were used further to obtain uniform manifold approximation and projection (UMAP) dimensionality reduction by RunUMAP function. Graph-based clustering was run using FindNeighbors and FindClusters with a resolution of 1.0 and the first 20 PCs as input, and the 21 clusters were identified. Differential expression analysis was implemented using MAST package.

During manual cluster annotation using canonical gene markers, a cluster corresponding to doublets of NK cells and macrophages was identified and excluded from the main figures. We provide this suggestion using DoubletFinder package (version 2.0.3) using

doubletFinder_v3 function (with parameters PCs = 10, pN = 0.25, pK = 0.005) for each sample separately (Figure S2A) (McGinnis et al., 2019).

For visualization purposes, the custom labels were assigned to several clusters by merging several clusters for simplification (e.g., clusters 1, 6, 20 were merged as T cells, clusters 2, 12 were merged as B cells and clusters 0, 3, 19 were merged as NK cells). The gene signature heatmap was drawn using the scaled data slot of the integrated assay.

For trajectory analysis, clusters assigned as monocytes and macrophages were used, and infer_trajectory function from the dyno package (version 0.1.2) was used with the available slingshot singularity container (version 1.0.3) (Saelens et al., 2019; Street et al., 2018). In order to exclude the technical bias across the samples, the data slot from the integrated assay was used as an input expression for trajectory inference. Monocytes cluster was used as a root cluster in terms of given priors to slingshot algorithm. Trajectory visualization was implemented after dimensionality reduction by UMAP using dimred_umap function.

Tissue histology

Adrenal glands were fixed in 4% paraformaldehyde containing 30% sucrose for a minimum of 24 h, and then embedded in OCT. Sections of 16μm were cut using cryostat CM350 between −20°C and −26°C. Sections were mounted on histological slides (Thermo, Superfrost Plus) and preserved at −20°C until further analysis. For paraffin histology analysis, adrenal glands were fixed as aforementioned. Samples were then dehydrated in ethanol using STP120 tissue processor and included in paraffin. Sections of 8μm were cut using Microtome Microme HM340E. Sections were mounted on histological slides and preserved at 4°C until further analysis.

Immunostaining

Sections were blocked for 1 h with 3% BSA and 0.1% Tween 20. Antibodies were diluted in PBS with 3% BSA, 0,1%Tween 20. The next day, sections were washed 3 times for 10 min with PBS and mounted in Antifade mounting medium with DAPI. For Bodipy staining, slides were incubated for 15 min with 400μM of Bodipy and then washed 3 times for 10 min and mounted in Antifade mounting medium with DAPI.

Immunofluorescence quantification method

IHC analyses and quantifications were performed manually using ImageJ. For counting of DAPI-positive cells, nuclei were automatically counted after setting a threshold and counted using the plugin “analyze particles” with a range of 1–20μm. Bodipy analysis was performed by cropping multiple areas in the sample and then setting a threshold. Bodipy-positive cells were counted using the plugin “analyze particles” with a range located between 0 and 300μm.

Fluorescence microscopy

The widefield microscope was a DM5500B upright stand (Leica, Germany). Acquisitions were performed using an Orca-ER camera (Hamamatsu, Japan). Mosaics were realized using a widefield/TIRF DMI6000 inverted stand microscope (Leica, Germany). Acquisitions were obtained using a DFC360 FX camera (Leica, Germany). The confocal microscope was a Nikon A1R confocal. Image acquisition was done with Leica AF suite software and analysed with image J.

Electron microscopy

The adrenal glands were fixed in 2.5% glutaraldehyde in 0.1 M cacodylate buffer (pH 7.4), rinsed with the same buffer and then post-fixed in osmium tetroxide (1% in cacodylate buffer). After rinsing with water, the specimens were dehydrated with acetone and embedded in Epon resin. The 80-nm ultrathin sections were contrasted with uranyl acetate and lead citrate for observation on a JEM 1400 JEOL Transmission Electron Microscope operating at 100 kV and equipped with an SIS Morada camera.

QUANTIFICATION AND STATISTICAL ANALYSIS

All data are represented in means ± SEM. Statistical analysis was performed with GraphPad Prism 8 as indicated in each figure legend. Details of the specific statistical test used are described in figure legends. The number of replicates used in the experiments are noted in figures, figure legends, or by graphs represented as dot plots, where n represents number of biological replicates. ns $p > 0.05$; *$p < 0.05$; **$p < 0.01$; ***$p < 0.001$; ****$p < 0.0001$.

Cell Reports, Volume *39*

Supplemental information

Unravelling the sex-specific diversity

and functions of adrenal gland macrophages

Bastien Dolfi, Alexandre Gallerand, Maria M. Firulyova, Yingzheng Xu, Johanna Merlin, Adélie Dumont, Alexia Castiglione, Nathalie Vaillant, Sandrine Quemener, Heidi Gerke, Marion I. Stunault, Patricia R. Schrank, Seung-Hyeon Kim, Alisha Zhu, Jie Ding, Jerome Gilleron, Virginie Magnone, Pascal Barbry, David Dombrowicz, Christophe Duranton, Abdelilah Wakkach, Claudine Blin-Wakkach, Burkhard Becher, Sophie Pagnotta, Rafael J. Argüello, Pia Rantakari, Svetoslav Chakarov, Florent Ginhoux, Konstantin Zaitsev, Ki-Wook Kim, Laurent Yvan-Charvet, Rodolphe R. Guinamard, Jesse W. Williams, and Stoyan Ivanov

Sup. Table 1

gene	p_val	avg_logFC (female over male)	pct.1	pct.2	p_val_adj
Xist	0	1.54951258733711	0.828	0	0
Rpl35	7.62774369759271e-40	0.332795131151709	0.975	0.916	1.1416443992187e-35
Apoc2	3.69158130258448e-26	0.332692544859788	0.379	0.167	5.52518973557819e-22
AA467197	6.22673290146039e-23	0.369648525877762	0.16	0.037	9.31955113361576e-19
Lyz2	2.17567047442043e-22	0.386902156024932	0.999	0.999	3.25632599906506e-18
Tsc22d3	6.49050153643698e-22	0.329842436397367	0.611	0.429	9.71433364958522e-18
Fxyd5	8.32816037140203e-22	0.27680960279782	0.944	0.892	1.24647576278774e-17
Ap1s2	1.73034399696515e-21	0.294384138791506	0.516	0.367	2.58980586025774e-17
Sla	3.91862476256913e-20	0.266959924479275	0.497	0.332	5.86500568213722e-16
Ly6a	1.0545988034916e-15	0.366701350118315	0.206	0.097	1.57841802918587e-11
Cebpb	8.00698976030681e-15	0.250943124974004	0.712	0.549	1.19840615742512e-10
Msrb1	5.40275053801241e-14	0.275219903461956	0.558	0.398	8.08629673024318e-10
Tmsb10	4.60848480745743e-12	0.380808925495099	0.801	0.725	6.89751921132154e-08
Ms4a4c	8.66898423345473e-11	0.25134064025716	0.4	0.286	1.29748687022117e-06
Chil3	4.8237747978503e-10	0.394563532638321	0.186	0.087	7.21974373994254e-06
Ifitm3	3.73254227181447e-08	0.266876571843615	0.88	0.835	0.000558649601822472
Ly6c2	3.5363257330096e-07	0.29637517528339	0.18	0.097	0.00529281872459546
Plac8	4.98692265535371e-07	0.460921076326878	0.294	0.193	0.0074639271382679
Apoe	7.50291402455461e-07	0.48830922785863	0.969	0.955	0.0112296114205509
S100a6	1.08763499763158e-06	0.276562829675311	0.591	0.514	0.0162786330095518

Table S1.
List of genes differentially expressed among myeloid cells in female and male mice. This table is related to figure 2.

Sup. Fig. 1

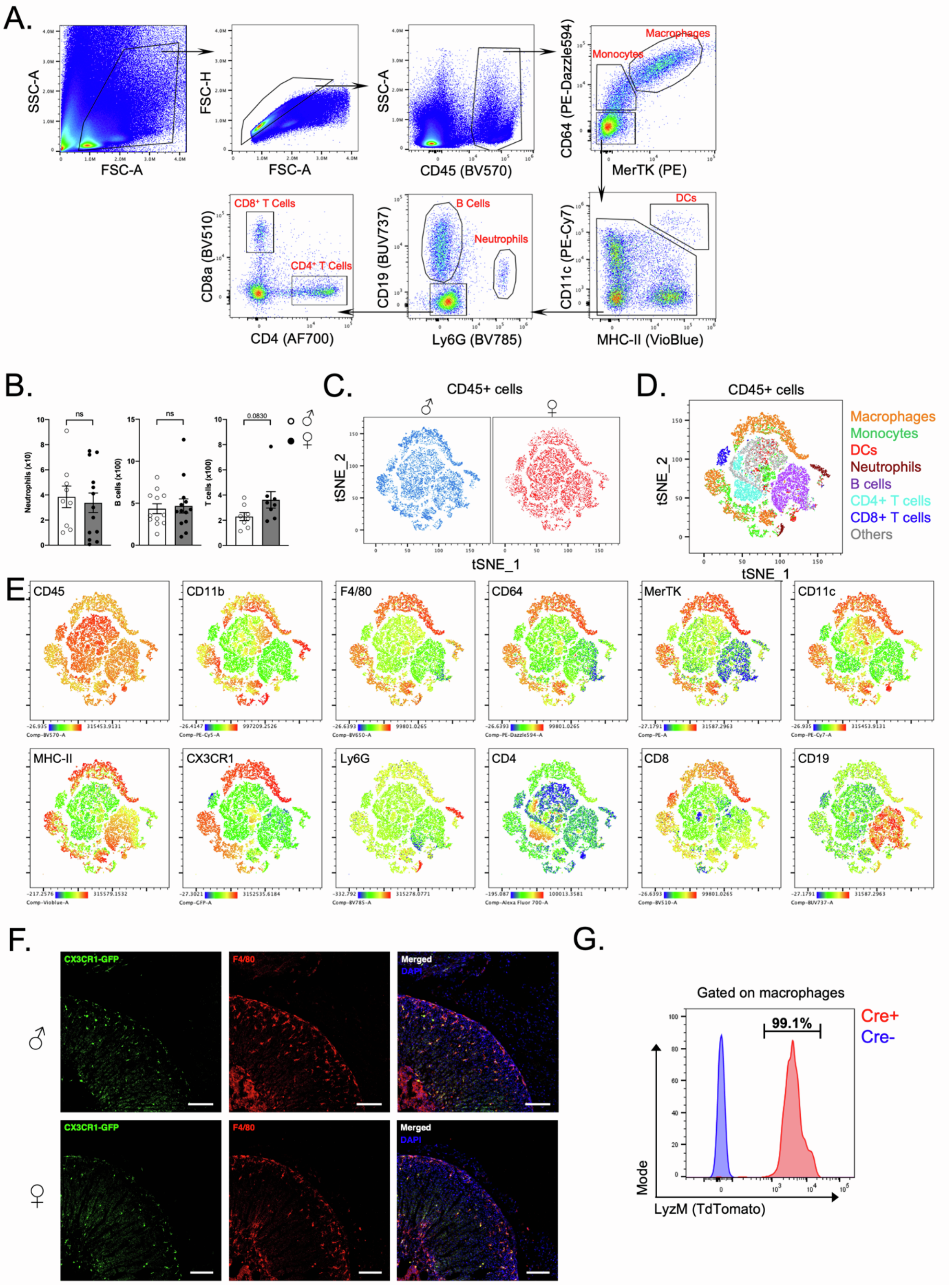

A.
SSC-A
FSC-A
FSC-H
FSC-A
SSC-A
CD45 (BV570)
CD64 (PE-Dazzle594)
MerTK (PE)
Macrophages
Monocytes
CD8a (BV510)
CD4 (AF700)
CD8+ T Cells
CD4+ T Cells
CD19 (BUV737)
Ly6G (BV785)
B Cells
Neutrophils
CD11c (PE-Cy7)
MHC-II (VioBlue)
DCs
B.
Neutrophils (x10)
B cells (x100)
T cells (x100)
ns
ns
0.0830
♂
♀
C.
CD45+ cells
♂
♀
tSNE_2
tSNE_1
D.
CD45+ cells
tSNE_2
tSNE_1
Macrophages
Monocytes
DCs
Neutrophils
B cells
CD4+ T cells
CD8+ T cells
Others
E.
CD45
CD11b
F4/80
CD64
MerTK
CD11c
MHC-II
CX3CR1
Ly6G
CD4
CD8
CD19
F.
CX3CR1-GFP
F4/80
Merged
DAPI
♂
♀
G.
Gated on macrophages
99.1%
Cre+
Cre-
Mode
LyzM (TdTomato)

Figure S1. Flow cytometry analysis of immune cell diversity in male and female adrenal glands. This figure in complementary to figure 1.

(**A**) Gating strategy used to identify adrenal gland immune cells. Concatenated data from 8 7-week-old female mice. (**B**) Quantification of neutrophils (♂ n=9 and ♀ n=13), B cells (♂ n=12 and ♀ n=13) and T cells (♂ n=8 and ♀ n=8) in male and female adrenal glands. Data pooled from one (T cells) or two (neutrophils and B cells) independent experiments. (**C**) Clustering of $CD45^+$ adrenal gland cells from male and female $CX3CR1^{GFP}$ mice using unsupervised t-distributed stochastic neighbor embedding (tSNE) analysis of flow cytometry data. Panel : CD40 (BUV395) ; CD24 (BUV496) ; CD80 (BUV615) ; CD19 (BUV737) ; F4/80 (BV650) ; Ly6G (BV785) ; CX3CR1 (GFP reporter) ; MerTK (PE) ; CD64 (PE-Dazzle594) ; CD11b (PE-Cy5) ; CD206 (PerCP-Cy5.5) ; Timd4 (PerCP-eFluor710) ; CD11c (PE-Cy7) ; Lyve1 (eFluor660) ; CD4 (AF700) ; CCR2 (APC-Fire750). (**D**) Identification of adrenal gland immune subsets. Cells were gated manually and overlaid on tSNE plot to identify clusters. Macrophages : $CD45^+$ $CD64^+$ $MerTK^+$. Monocytes : $CD45^+$ $MerTK^-$ $CD11b^+$ $CX3CR1^+$. Dendritic Cells (DCs) : $CD45^+$ $MerTK^-$ $CD11c^+$ $MHC\text{-}II^+$. Neutrophils : $CD45^+$ $CD11b^+$ $Ly6G^+$. B cells : $CD45^+$ $MerTK^-$ $CD11c^-$ $MHC\text{-}II^+$ $CD19^+$. CD4+ T cells : $CD45^+$ $MerTK^-$ $MHC\text{-}II^-$ $CD4^+$. CD8+ T cells : $CD45^+$ $MerTK^-$ $MHC\text{-}II^-$ $CD8^+$. (**E**) tSNE representation of the expression levels of CD45, CD11b, F4/80, CD64, MerTK, CD11c, MHC-II, CX3CR1, Ly6G, CD4, CD8 and CD19 among adrenal gland $CD45^+$ cells. (**F**) Microscopy analysis of F4/80 staining in adrenal glands of $CX3CR1^{GFP/+}$ mice. Scale bar : 100μm. (**G**) Flow cytometry analysis of TdTomato expression in adrenal gland macrophages from 8-week-old male $Lyz2^{cre}$ x $R26^{TdTomato}$ mice. Data from one experiment. All data are represented in means ± SEM. Statistical analysis was performed using two-tailed Mann-Whitney tests. ns p>0.05 ; * p<0.05 ; ** p<0.01 ; *** p<0.001 ; **** p<0.0001.

Sup. Fig. 2

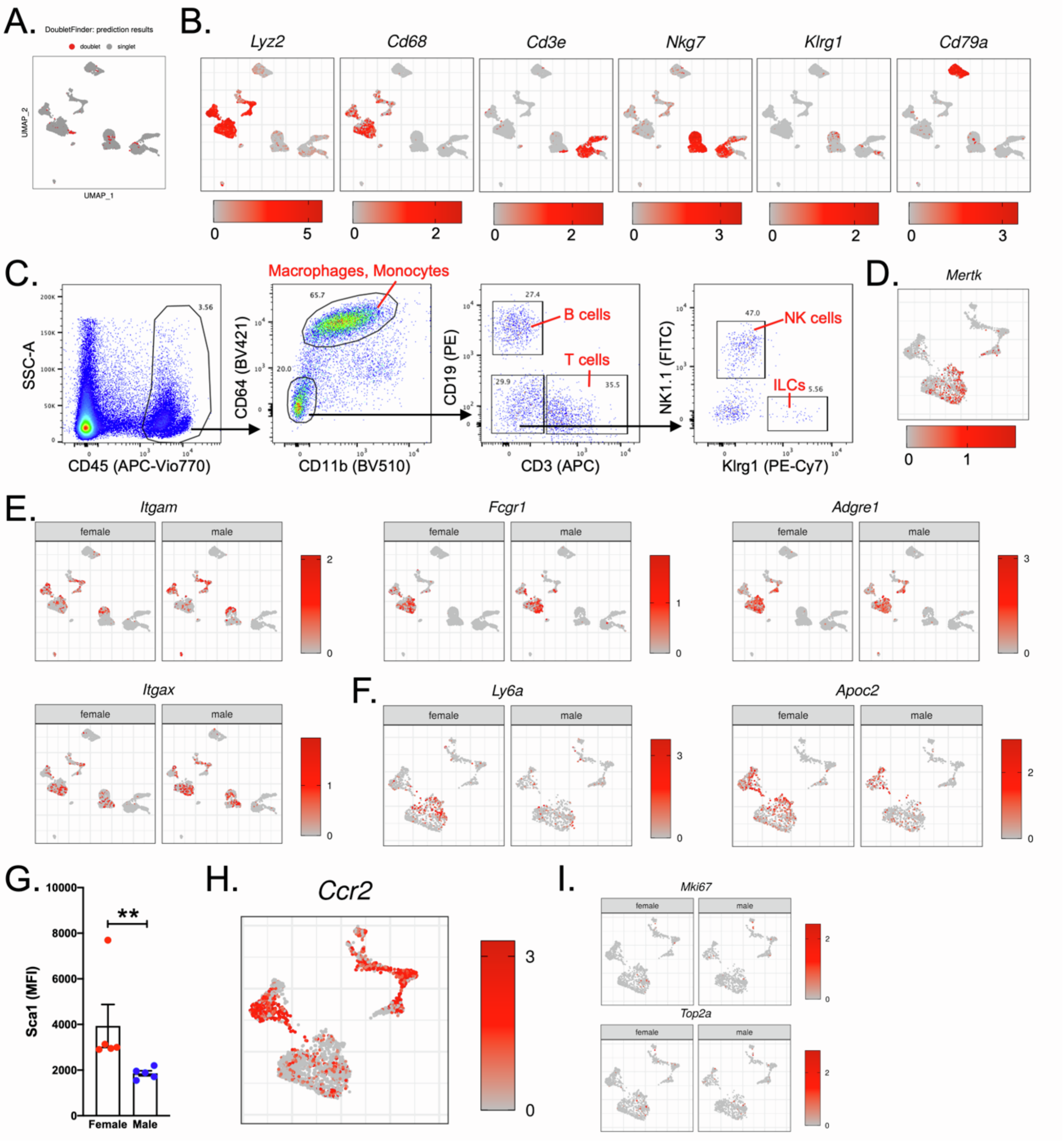

A.
DoubletFinder: prediction results
doublet
singlet
UMAP_2
UMAP_1
B.
Lyz2
Cd68
Cd3e
Nkg7
Klrg1
Cd79a
C.
Macrophages, Monocytes
B cells
T cells
NK cells
ILCs
SSC-A
CD45 (APC-Vio770)
CD64 (BV421)
CD11b (BV510)
CD19 (PE)
CD3 (APC)
NK1.1 (FITC)
Klrg1 (PE-Cy7)
3.56
65.7
20.0
27.4
29.9
35.5
47.0
5.56
D.
Mertk
E.
Itgam
Fcgr1
Adgre1
Itgax
female
male
F.
Ly6a
Apoc2
G.
**
Sca1 (MFI)
Female
Male
H.
Ccr2
I.
Mki67
Top2a

Figure S2. Single cell RNA-seq analysis of adrenal gland immune cell diversity. This figure is complementary to figure 2.

(**A**) Identification of doublets in scRNA-seq data using the DoubletFinder package. Doublets were removed from analysis. (**B**) scRNA-seq analysis of genes used to identify cell types (*Lyz2*, *CD68*, *CD3e*, *Nkg7*, *Klrg1* and *CD79a*). (**C**) Flow cytometry plots validating the presence of immune cell types corresponding to clusters identified in the scRNA-seq analysis. (**D**) scRNA-seq analysis of *Mertk* expression among adrenal gland myeloid cells. (**E**) scRNA-seq analysis of myeloid cell canonical markers by adrenal gland $CD45^+$ cells from 7-week-old male and female mice. (**F**) scRNA-seq analysis of sex-enriched genes in adrenal gland myeloid cells. (**G**) Flow cytometry analysis of Sca1 expression in female and male mice (n=5 mice per group). Data pooled from two independent experiments. (**H**) scRNA-seq analysis of *CCR2* expression among adrenal gland myeloid cells. (**I**) scRNA-seq analysis of *Mki67* and *Top2a* mRNA expression. All data are represented in means ± SEM. Statistical analysis was performed using two-tailed Mann-Whitney tests. ns p>0.05 ; * p<0.05 ; ** p<0.01 ; *** p<0.001 ; **** p<0.0001.

Sup. Fig. 3

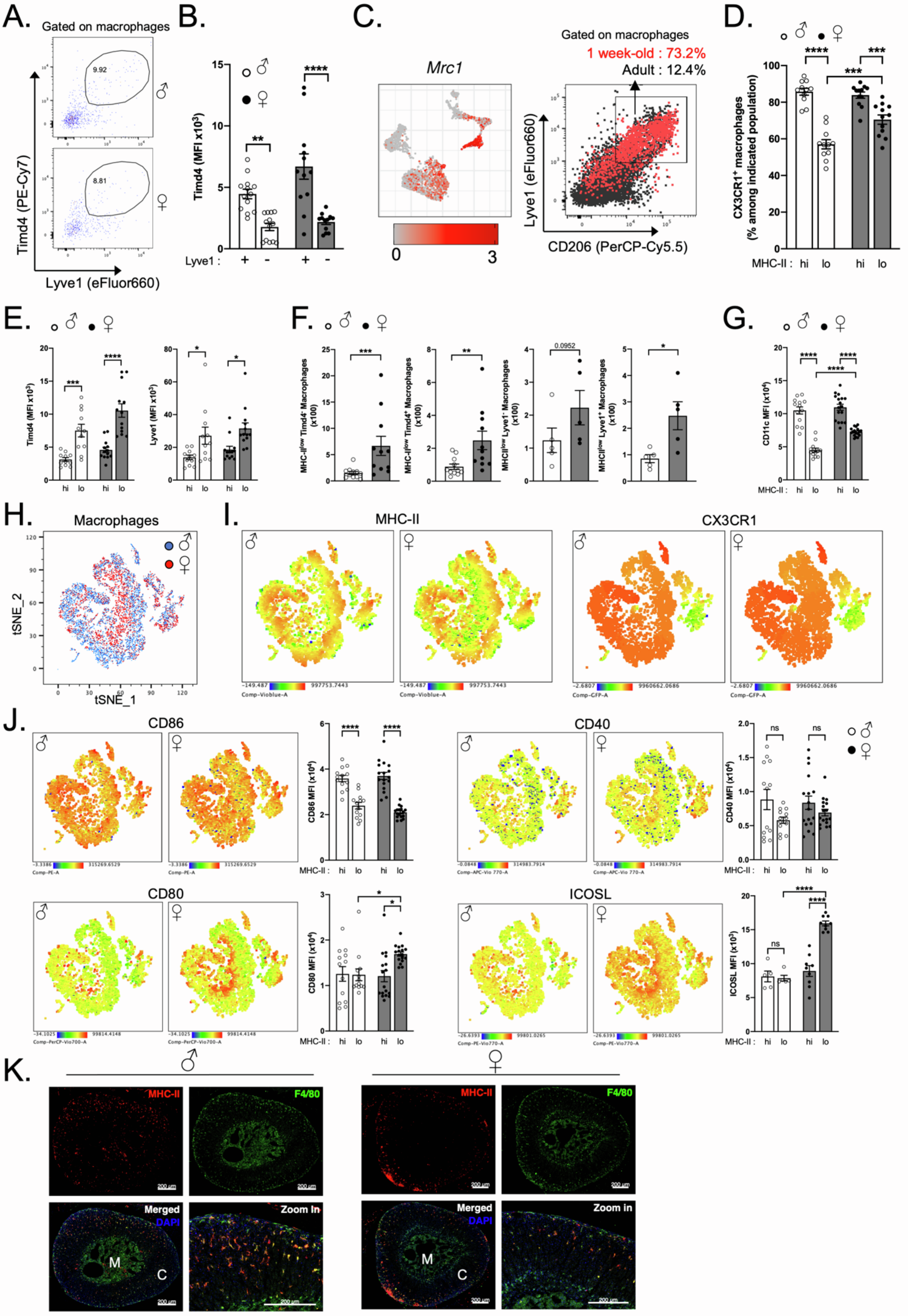
A.
Gated on macrophages
Timd4 (PE-Cy7)
Lyve1 (eFluor660)
B.
Timd4 (MFI x10³)
Lyve1 :
C.
Mrc1
Gated on macrophages
1 week-old : 73.2%
Adult : 12.4%
Lyve1 (eFluor660)
CD206 (PerCP-Cy5.5)
D.
CX3CR1⁺ macrophages (% among indicated population)
MHC-II :
E.
Timd4 (MFI x10³)
Lyve1 (MFI x10³)
F.
0.0952
G.
CD11c MFI (x10⁴)
H.
Macrophages
tSNE_2
tSNE_1
I.
MHC-II
CX3CR1
J.
CD86
CD40
CD80
ICOSL
CD86 MFI (x10⁴)
CD40 MFI (x10⁴)
CD80 MFI (x10⁴)
ICOSL MFI (x10³)
K.
MHC-II
F4/80
Merged
DAPI
Zoom In
Zoom in
M
C

Figure S3. MHC-II expression identifies two macrophage populations with sex-specific abundance and distribution. This figure is complementary to figures 3 and 4.

(**A**) Representative plots showing adrenal gland macrophage Timd4 and Lyve1 expression in male and female mice. Data representative of at least 3 independent experiments. (**B**) Mean Timd4 fluorescence intensity among Lyve1$^+$ and Lyve1$^-$ macrophage subsets in male and female mice. ♂ n=13 and ♀ n=12. Data pooled from 2 independent experiments. (**C**) (Left) scRNA-seq analysis of *Mrc1* mRNA expression and (Right) representative plot showing adrenal gland macrophage Lyve1 and CD206 expression in 1- and 9-week-old male mice. Data from one experiment. (**D**) Proportions of CX3CR1$^+$ cells among MHC-II^{high} and MHC-II^{low} adrenal gland macrophages from male and female CX3CR1^{GFP} mice. ♂ n=11 and ♀ n=12. Data pooled from 2 independent experiments. (**E**) Mean Timd4 and Lyve1 fluorescence intensity among MHC-II^{high} and MHC-II^{low} adrenal gland macrophages from male and female mice. ♂ n=12 and ♀ n=13. Data pooled from 2 independent experiments. (**F**) Quantification of MHC-II^{low} adrenal gland macrophage subsets according to Lyve1 and Timd4 expression in 7-week-old male and female mice. Timd4 subsets : ♂ n=11 and ♀ n=11. Data pooled from 2 independent experiments. Lyve1 subsets : ♂ n=5 and ♀ n=5. Data from one experiment. (**G**) Mean CD11c fluorescence intensity among MHC-II^{high} and MHC-II^{low} adrenal gland macrophages from male and female mice. ♂ n=13 and ♀ n=17. Data pooled from 3 independent experiments. (**H**) Clustering of adrenal gland macrophages from male and female CX3CR1^{GFP} mice using unsupervised t-distributed stochastic neighbor embedding (tSNE) analysis of flow cytometry data. Panel : CD24 (BUV496) ; CD19 (BUV737) ; MHC-II (VioBlue) ; CD11b (BV510) ; CD45 (BV570) ; F4/80 (BV650) ; CX3CR1 (GFP reporter) ; CD86 (PE) ; CD64 (PE-Dazzle594) ; CD11c (PE-Cy5) ; CD80 (PerCP-Vio700) ; ICOSL (PE-Vio770) ; Ly6C (APC) ; CD40 (APC-Vio770). Data representative of 3 independent experiments. (**I**) tSNE representation of MHC-II and CX3CR1 expression levels in adrenal gland macrophages from male and female CX3CR1^{GFP} mice. Data representative of 3 independent experiments. (**J**) tSNE representation and quantification of CD86, CD80, CD40 and ICOSL expression levels in adrenal gland macrophages from male and female CX3CR1^{GFP} mice. ♂ n=13 and ♀ n=17. Pooled data from 2 (ICOSL) or 3 (CD86, CD80, CD40) independent experiments. (**K**) Fluorescence microscopy analysis of F4/80 and MHC-II expression in adrenal glands from 7-week-old male and female wild-type mice. Scale bar : 200μm. M (medulla); C (cortex). Data representative of at least 3 independent experiments. All data are represented in means ± SEM. Statistical analysis was performed using two-way ANOVA with Bonferroni's post-test (panels B, D, E, F, G and J) or two-tailed Mann-Whitney tests (panel G). ns p>0.05 ; * p<0.05 ; ** p<0.01 ; *** p<0.001 ; **** p<0.0001.

Sup. Fig. 4

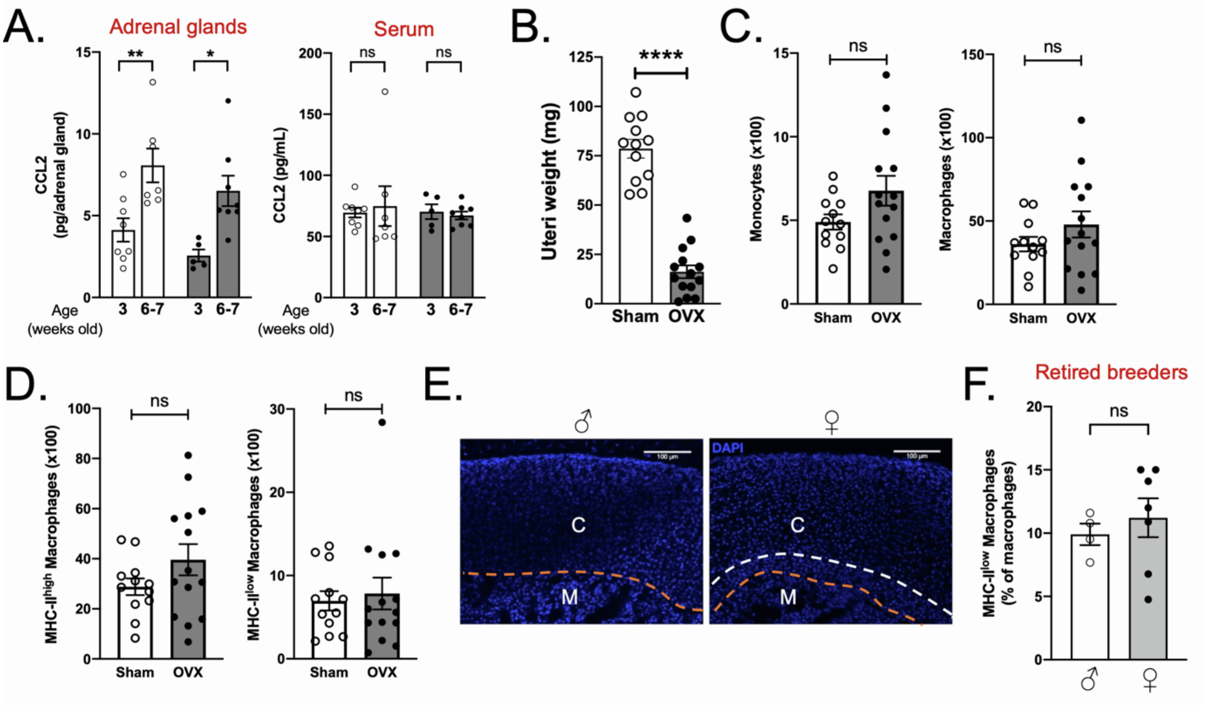

Figure S4. Ovariectomy does not affect adrenal gland macrophage content. This figure is complementary to figure 5.

(**A**) CCL2 levels in adrenal gland homogenates and serum from 3 (♂ n=8 and ♀ n=5) or 6 to 7 (♂ n=7 and ♀ n=8) week-old male and female wild-type mice. Data pooled from 4 independent experiments. (**B**) Weight of uteri from sham-operated (n=12) and OVX (n=14) mice. Data pooled from 2 independent experiments. (**C**) Quantification of adrenal gland monocytes and macrophages in ovariectomized (n=14) and sham-operated (n=12) wild-type mice. Data pooled from 2 independent experiments. (**D**) Quantification of adrenal gland MHC-II^{low} and MHC-II^{high} macrophages in ovariectomized (n=14) and sham-operated (n=12) wild-type mice. Data pooled from 2 independent experiments. (**E**) Confocal microscopy analysis of adrenal glands from male and female 4-week-old wild-type mice. Orange dots indicate the border between the cortex (C) and the medulla (M). The X-Zone is comprised between white and orange dots. Scale bar : 100um. (**F**) Proportions of MHC-II^{low} adrenal gland macrophages in male and female retired breeders (♂ n=4 and ♀ n=7). Data pooled from 2 independent experiments. All data are represented in means ± SEM. Statistical analysis was performed using two-way ANOVA with Bonferroni's post-test (panel A) or two-tailed Mann-Whitney tests (panels B, C, D and F). ns p>0.05 ; * p<0.05 ; ** p<0.01 ; *** p<0.001 ; **** p<0.0001.

Sup. Fig. 5

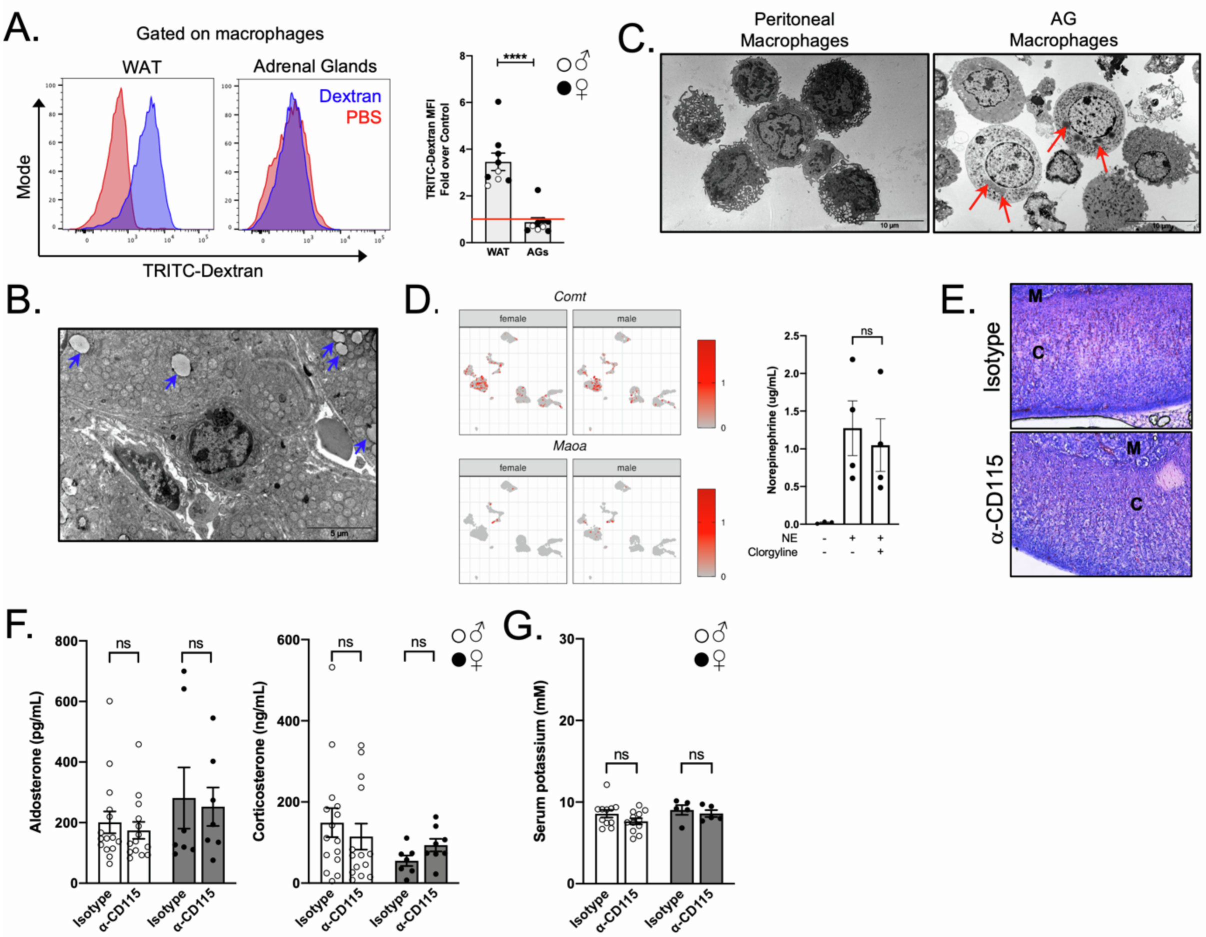

A.
Gated on macrophages
WAT
Adrenal Glands
Dextran
PBS
Mode
TRITC-Dextran
TRITC-Dextran MFI Fold over Control
WAT
AGs
****
C.
Peritoneal Macrophages
AG Macrophages
B.
D.
Comt
female
male
Maoa
Norepinephrine (ug/mL)
ns
NE
Clorgyline
E.
Isotype
α-CD115
M
C
F.
Aldosterone (pg/mL)
Corticosterone (ng/mL)
Isotype
α-CD115
G.
Serum potassium (mM)

Figure S5. Macrophage depletion does not alter adrenal gland architecture and hormone production at steady state. This figure is complementary to figure 6.
(**A**) Representative flow cytometry plots (left) and quantification (right) of TRITC-Dextran uptake by adipose tissue and adrenal gland macrophages 20 minutes after intravenous injection of 1mg 65-85kDa TRITC-Dextran. (♂ n=3 and ♀ n=6). Data from one experiment. (**B**) Electron microscopy analysis of adrenal gland macrophages. Blue arrows indicate the presence of lipid droplets in stroma. Scale bar : 5μm. (**C**) Electron microscopy analysis of purified peritoneal and adrenal gland macrophages. Red arrows indicate the presence of lipid droplets in macrophages. Scale bar : 10μm. Data from one experiment. (**D**) (Left) scRNA-seq analysis of *Maoa* and *Comt* mRNA expression and (Right) norepinephrine levels after overnight culture of cell-sorted adrenal gland macrophages in the presence/absence of norepinephrine and/or clorgyline. n=3 (baseline) or 4 (+NE) biological replicates. Data from 3 pooled experiments. (**E**) Microscopy analysis of adrenal gland morphology in isotype control-treated and α–CD115-treated wild-type mice using H&E staining. Data from one experiment. (**F**) Serum aldosterone and corticosterone levels in isotype control-treated (♂ n=15 and ♀ n=7) and α–CD115-treated (♂ n=14 and ♀ n=7-8) male and female wild-type mice. (**G**) Serum K^+ levels in isotype control-treated (♂ n=11 and ♀ n=5) and α–CD115-treated (♂ n=12 and ♀ n=5) male and female wild-type mice. Pooled data from 3 independent experiments. Statistical analysis was performed using two-way ANOVA with Bonferroni's post-test (panels F and G), one-way ANOVA with Bonferroni's post-test (panel D) or two-tailed Mann-Whitney tests (panel A). ns $p>0.05$; * $p<0.05$; ** $p<0.01$; *** $p<0.001$; **** $p<0.0001$.

Sup. Fig. 6

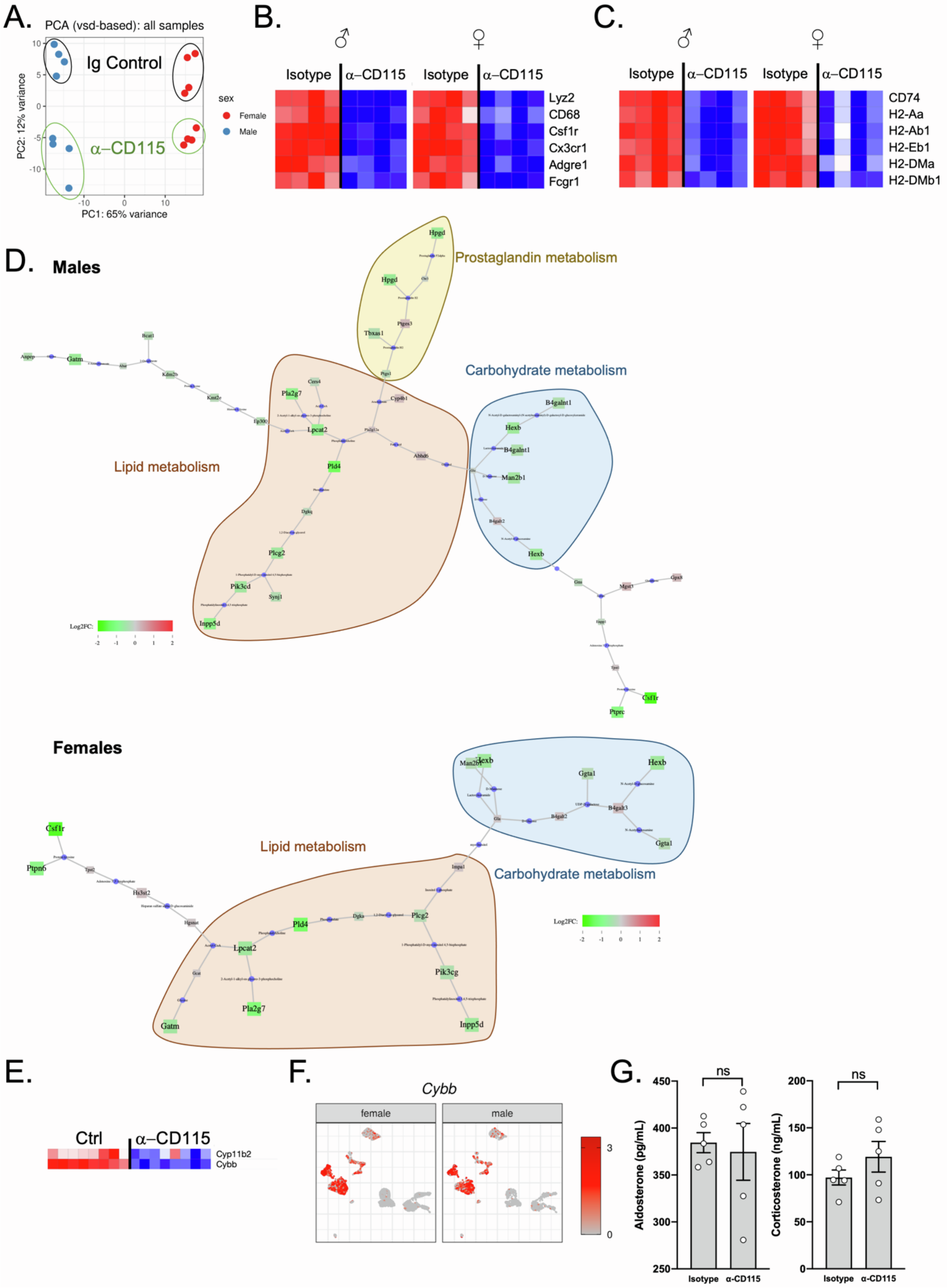
A.
PCA (vsd-based): all samples
Ig Control
α–CD115
sex
Female
Male
PC2: 12% variance
PC1: 65% variance
B.
♂
♀
Isotype
α–CD115
Lyz2
CD68
Csf1r
Cx3cr1
Adgre1
Fcgr1
C.
CD74
H2-Aa
H2-Ab1
H2-Eb1
H2-DMa
H2-DMb1
D.
Males
Prostaglandin metabolism
Carbohydrate metabolism
Lipid metabolism
Log2FC:
Females
Lipid metabolism
Carbohydrate metabolism
E.
Ctrl
α–CD115
Cyp11b2
Cybb
F.
Cybb
female
male
G.
ns
Aldosterone (pg/mL)
Corticosterone (ng/mL)
Isotype
α-CD115

Figure S6. Macrophage depletion affects adrenal gland lipid metabolism. This figure is complementary to figure 6.
(**A**) Principal Component Analysis (PCA) of adrenal gland bulk RNA-seq from isotype control-treated and α–CD115-treated male and female wild-type mice. (**B**) RNA-seq analysis of myeloid cell-related genes in adrenal glands from isotype control-treated and α–CD115-treated male and female wild-type mice. (**C**) RNA-seq analysis of MHC-II-related genes in adrenal glands from isotype control-treated and α–CD115-treated male and female wild-type mice. (**D**) Transcriptional analysis of metabolic pathways in adrenal glands from α–CD115 or isotype control treated male and female mice. The size of nodes represent the significance of changes in gene expression : bigger size of nodes means lower p-values. A high resolution version of this panel is supplied as supplementary item 3. (**E**) RNA-seq analysis of *Cyp11b2* and *Cybb* expression in adrenal glands from isotype control-treated and α–CD115-treated wild-type mice. (**F)** scRNA-seq analysis of *Cybb* mRNA expression. (**G**) Serum aldosterone and corticosterone levels in α–CD115 (n=5) or isotype control-treated (n=5) female wild-type mice submitted to a 12-hour cold challenge. Data from one experiment. Statistical analysis was performed using two-tailed Mann-Whitney tests (panel G). ns $p>0.05$.
